# Supplementary material for: Benchmarking of the Oxford Nanopore MinION sequencing for quantitative and qualitative assessment of cDNA populations
Source: Sci Rep. 2016 Aug 24;6:31602. doi: 10.1038/srep31602 (PMC4995519; doi:10.1038/srep31602)
Supplement: Supplementary Information [file srep31602-s1.pdf]

Supplementary Information

Benchmarking of the Oxford Nanopore MinION sequencing for quantitative and qualitative assessment of cDNA populations

Spyros Oikonomopoulos, Yu Chang Wang, Haig Djambazian, Dunarel Badescu, Jiannis Ragoussis*

Department of Human Genetics, McGill University and Genome Quebec Innovation Centre, McGill University, Montréal, Québec, Canada

*corresponding author (ioannis.ragoussis@mcgill.ca)

Table of Contents

Supplementary Figures **1**

Supplementary Figure S1. Basecalling accuracy for the sequenced reads from the different ONT MinION experiments..1

Supplementary Figure S2. Comparison of the percentage of complement, 2D reads and high quality reads reported in this manuscript relative to other published studies. 3

Supplementary Figure S3. Number of MinION reads accumulated over the sequencing time for the ERCC experiment number 4. 5

Supplementary Figure S4. Estimation of ERCC transcript abundance with the Illumina HiSeq 2500 or MiSeq platform. 7

Supplementary Figure S5. Comparison of the ERCC cDNA abundance between the PacBio RS II platform against the expected number of RNA molecules as provided from the manufacturer (Ambion). 8

Supplementary Figure S6. cDNA abundance of the 92 ERCC transcripts from the ONT MinION sequencing (barplots)..10

Supplementary Figure S7. Similar with Supplementary Fig. S6 but the values are log10 transformed..12

Supplementary Figure S8. A-C) cDNA abundance of the 92 ERCC transcripts from the ONT MinION sequencing (barplots). 13

Supplementary Figure S9 (previous page). Consistency of comparisons between the low quality template reads against either the high quality template reads (A-C), the low quality complement reads (D-F) and the low quality 2D reads (G-I) for the ERCC experiments number 2 (B, E, H), number 3 (C, F, I) and number 4 (A, D, G). 18

Supplementary Figure S10. Consistency of comparisons between the template reads against either the complement reads (A) or the 2D reads (B) for the ERCC experiment number 1..20

Supplementary Figure S11. cDNA abundance of the 92 ERCC transcripts from the ONT MinION sequencing (barplots) using different aligners and different alignment parameters (Supplementary Table S2). 23

Supplementary Figure S12. Similar with Supplementary Fig. S11 but the values are log10 transformed..25

Supplementary Figure S13. Comparison of different aligners and alignment parameters 26

Supplementary Figure S14. Positional distribution on the ERCC reference transcripts of the Illumina HiSeq 2500 or MiSeq fragments. 27
Supplementary Figure S15. Full length processivity of the ERCC cDNA molecules from the ONT MinION platform. 28
Supplementary Figure S16. Length of the ONT MinION reads over the sequencing time. 30
Supplementary Figure S17. Read length frequency distribution of the cDNA molecules sequenced on the ONT MinION platform from the ERCC experiments 1, 2, 3, 4. 32
Supplementary Figure S18. Effect of the GC content and ERCC length on the estimation of the ERCC cDNA abundance with the PacBio RS II platform. 34
Supplementary Figure S19. Deviation of the ERCC expression level estimates with the Illumina HiSeq 2500 or MiSeq platforms from the expected Ambion RNA molecular counts as a function of the GC content (A, C, E) and the ERCC length (B, D, F). 37
Supplementary Figure S20. Fragment size distribution of the Illumina libraries. 38
Supplementary Figure S21. Effect of GC content and length on the number of high quality reads acquired from each ERCC transcript. 39
Supplementary Figure S22. Limit of detection of the ONT MinION platform as calculated from the ERCC experiments 1, 2, 3, 4. 40
Supplementary Figure S23. Full length sequencing of the RNA Spike-1 cDNA molecules from the complex HEK-293 cDNA population. 42
Supplementary Figure S24. Read length frequency distribution of the HEK-293 cDNA molecules sequenced on the ONT MinION and PacBio RS II platforms. 44
Supplementary Figure S25. Similarity between the isoforms detected on the ONT MinION, PacBio RS II and Illumina HiSeq 2500 platfoms. 46
Supplementary Figure S26 (previous page). Effect of different quantification methods in the agreement between the Illumina and the ONT MinION or the PacBio RS II estimated cDNA abundance for the HEK-293 isoforms. 49
Supplementary Figure S27 . Effect of different quantification methods in the agreement between the Illumina and the ONT MinION or the PacBio RS II estimated cDNA abundance for the HEK-293 genes. 50
Supplementary Figure S28. Estimation of the HEK-293 cDNA gene abundance with three sequencing platforms. 51
Supplementary Figure S29. Estimation of the HEK-293 cDNA gene abundance with three sequencing platforms after removing a set of problematic genes. ..53

Supplementary Figure S30. DNA sequence of the template switch primer, the oligo-dT30V primer and the cDNA amplification primer used during the ERCC cDNA synthesis. 55

Supplementary Figure S31 (previous page). Electropherogram profile of the ERCC RNA (A) and cDNA (B-E) populations. 57

Supplementary Figure S32. Performance of the five ONT MinION flow cells used in this study. 58

Supplementary Tables 60

Supplementary Table S1 (previous page). Summary results of the comparisons presented in Supplementary Fig. S9 and Supplementary Fig. S10. 62

Supplementary Table S2 (previous page). Alignment parameters for the indicated aligners as are retrieved from the literature. 64

Supplementary Table S3. Amount of cDNA available at each step of the ONT library preparation procedure from the different MinION experiments 65

Supplementary Table S4 (previous page). Cases where the different platforms can be used either exclusively or interchangeably. 67

Supplementary Table S5. Number of ERCC cDNA fragments sequenced on the Illumina platforms. 68

Supplementary Table S6. Amount of cDNA loaded on the flow cell during the different MinION experiments. 70

Supplementary Table S7. Number of sequenced and aligned PacBio RS II reads from the HEK-293 cDNA and the ERCC cDNA 71

Supplementary text 73

I. Variability in the ONT MinION flowcell performance 73

II. Estimation of ERCC transcript abundance with the Illumina HiSeq 2500 or MiSeq platforms. 74

III. Assessment of the quality of the ERCC cDNA library loaded on the MinION flowcells 77

IV. Effect of the ERCC transcript length and GC content in the ERCC cDNA abundance estimation with the PacBio RS II platform 78

V. Limit of detection of the ERCC transcripts for the variable sequencing depth of the ONT MinION experiments. 80

VI. The Illumina short read cDNA abundance estimation is not considerably altered with different short read alignment methods and gene/isoform expression estimation methods. 81

VII. Fragment multi-mapping as a potential confounding factor of Illumina abundance estimation. 85

Supplementary methods **87**
I. Tagmentation of the ERCC cDNA for Illumina HiSeq 2500 or MiSeq sequencing 87
II. Alignment of the ERCC cDNA fragments sequenced on the Illumina platform... 88
III. Simulating fragments from the ERCC transcript database..................... 89
IV. Barcoding the ERCC cDNA population 90
V. Bioinformatic processing of the UMI barcoded ERCC reads sequenced on the Illumina platform in order to isolate fragments from the 5' or the 3' end of the cDNA molecules 94
VI. Considerations during the ONT MinION sequencing of the ERCC cDNA population 97
VII. Alignment of the ONT MinION reads 99
VIII. Subsampling the ONT MinION low quality template reads 101
IX. HEK-293 cDNA production 107
X. ONT MinION sequencing of the HEK-293 cDNA population................... 109
XI. Identification of the similarity in the TSS and TES positions between the PacBio RS II and ONT MinION platforms for the HEK-293 cDNA library. 111
XII. Alignment of the HEK-293 Illumina HiSeq 2500 cDNA reads 112
XIII. PacBio RS II processing of raw read files... 114
XIV. Bias quantification.. 116

Supplementary Figures

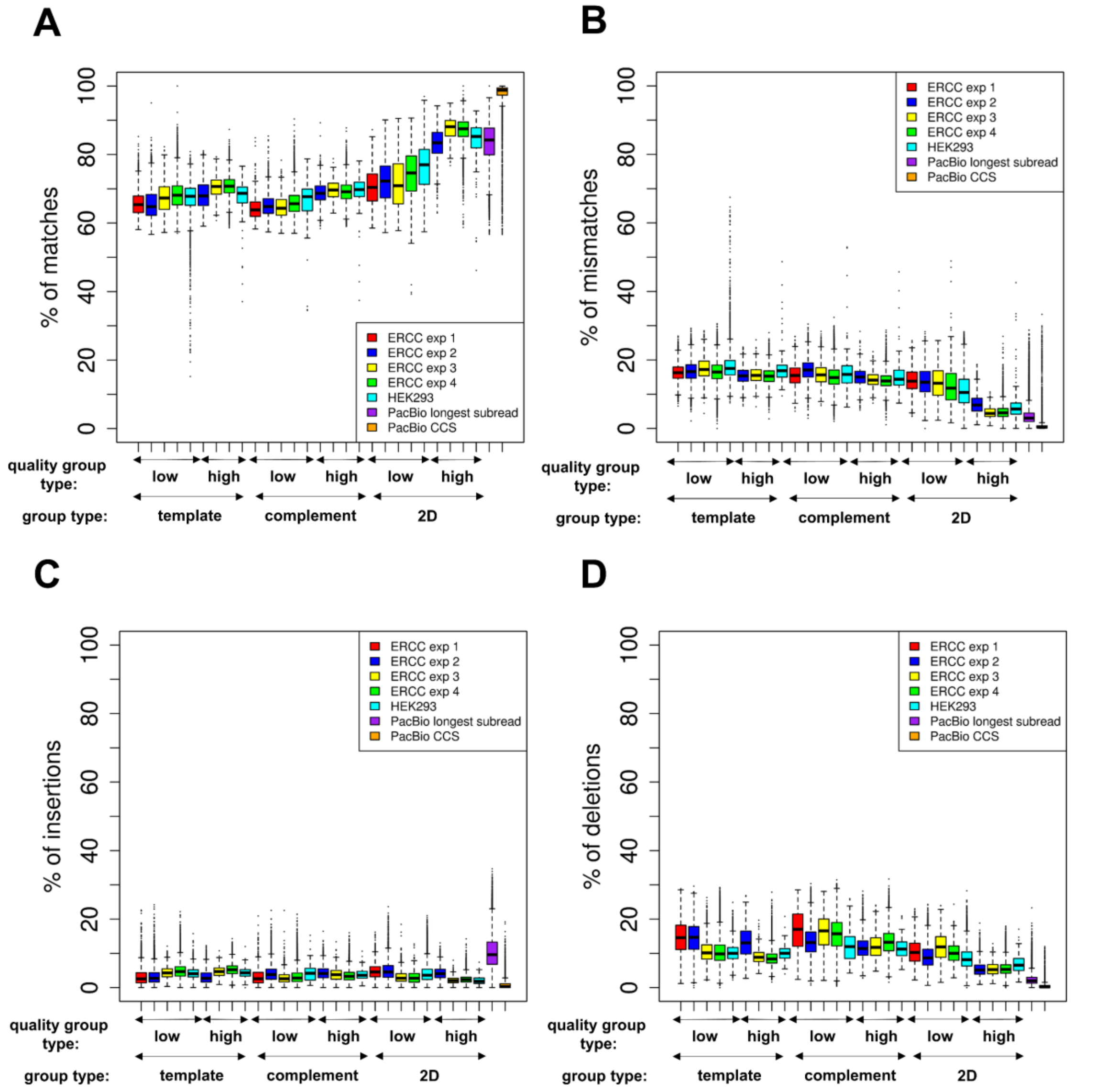

Supplementary Figure S1. Basecalling accuracy for the sequenced reads from the different ONT MinION experiments. The sequenced reads are separated in the template, complement and 2D reads group for both the high and low basecalling quality categories. The PacBio RS II derived longest subreads and

the CCS reads of the HEK-293 cDNA library are also presented. For comparison the PacBio RS II reads are aligned against the reference database with the same aligner, the same alignment parameters and the same filtering options as the ONT MinION reads.

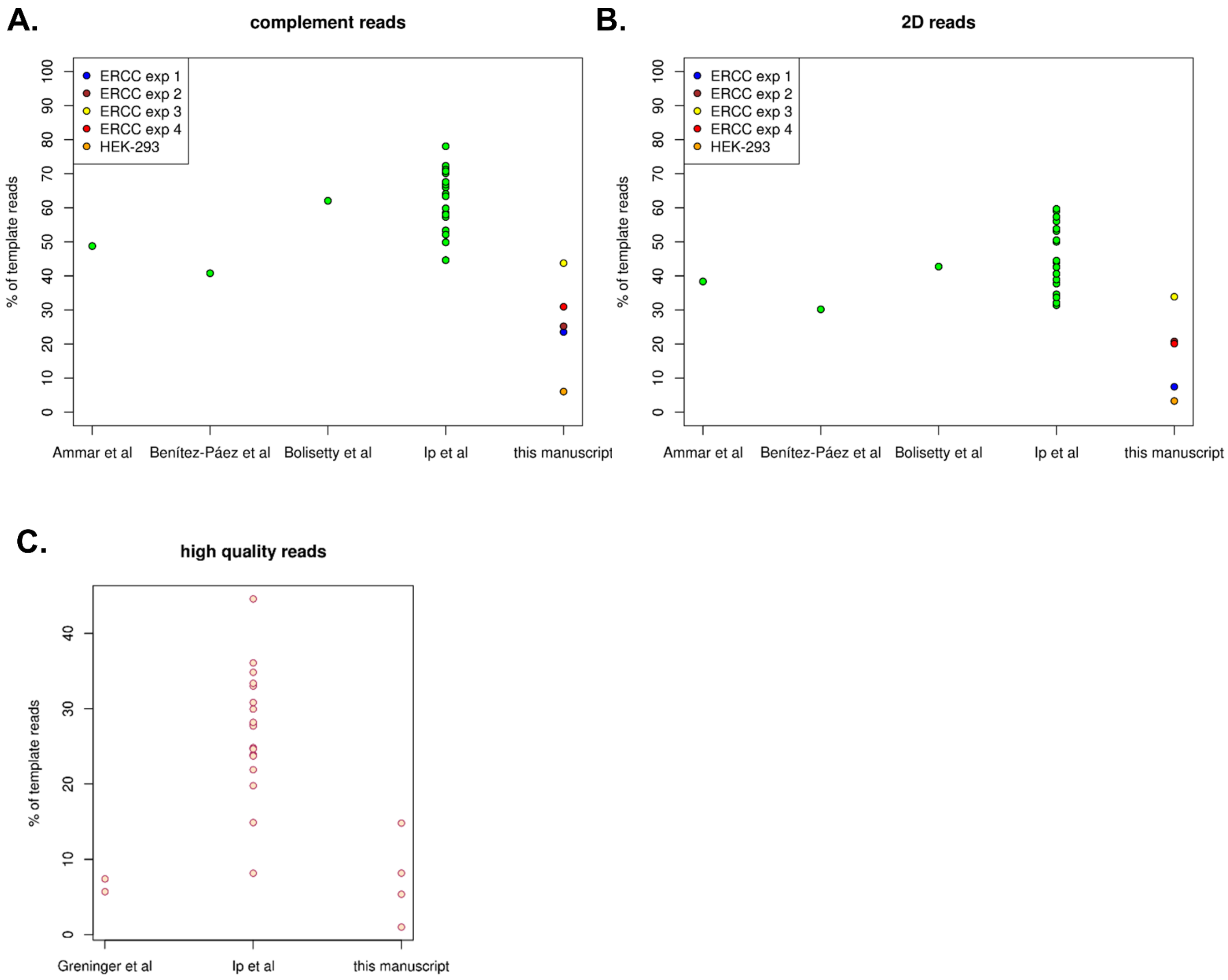

Supplementary Figure S2. Comparison of the percentage of complement, 2D reads and high quality reads reported in this manuscript relative to other published studies. The studies presented here are the ones where the number of complement, 2D and high quality reads is reported in their respective manuscript. In all the studies presented here the version r7.3 of the ONT MinION platform was used to produce the data. The studies sequenced material from the following sources: Ip et al[1] sequenced genomic DNA (average length: 6 kb). Bolisetty et al[2] sequenced cDNA amplicons (variable length : ~0.6 kb, ~1.8 kb, ~4

kb). Ammar et al[3], Benítez-Páez et al[4] and Greninger er al[5] sequenced DNA amplicons (~4.5 kb, ~1 kb, ~600 bp in length respectively). As far as it concerns the library preparation kit version Ammar et al[3] and Bolisetty et al[2] used the SQK-MAP003 genomic DNA sequencing kit, Ip et al[1] and Benítez-Páez et al[4] used the SQK-MAP005 genomic DNA sequencing kit and Greninger er al[5] used the SQK-MAP004 genomic DNA sequencing kit. The data from this manuscript are also presented as separate column.

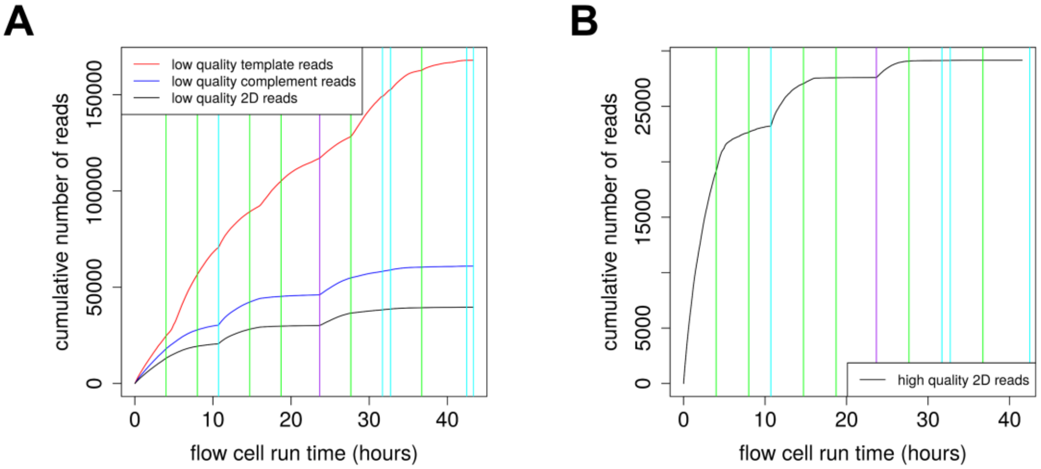

Supplementary Figure S3. Number of MinION reads accumulated over the sequencing time for the ERCC experiment number 4. The number of low quality template, complement and 2D reads is presented (A). For the high quality reads only the 2D read accumulation over time is presented (B) as the graphs for the template and complement are exactly the same.

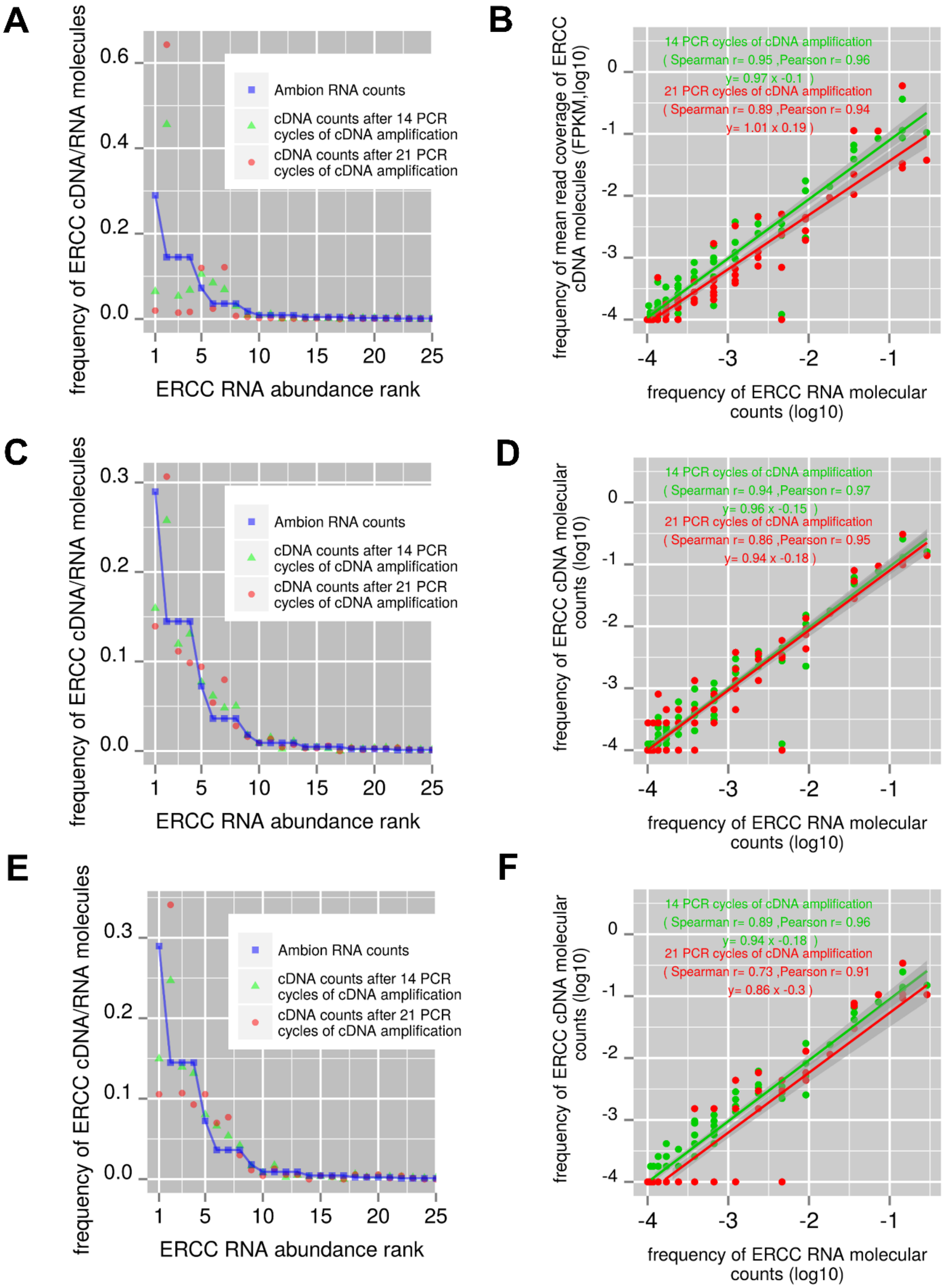
A
B
C
D
E
F
Ambion RNA counts
cDNA counts after 14 PCR cycles of cDNA amplification
cDNA counts after 21 PCR cycles of cDNA amplification
frequency of ERCC cDNA/RNA molecules
ERCC RNA abundance rank
14 PCR cycles of cDNA amplification
(Spearman r= 0.95 ,Pearson r= 0.96
y= 0.97 x -0.1)
21 PCR cycles of cDNA amplification
(Spearman r= 0.89 ,Pearson r= 0.94
y= 1.01 x 0.19)
frequency of mean read coverage of ERCC cDNA molecules (FPKM,log10)
frequency of ERCC RNA molecular counts (log10)
14 PCR cycles of cDNA amplification
(Spearman r= 0.94 ,Pearson r= 0.97
y= 0.96 x -0.15)
21 PCR cycles of cDNA amplification
(Spearman r= 0.86 ,Pearson r= 0.95
y= 0.94 x -0.18)
frequency of ERCC cDNA molecular counts (log10)
14 PCR cycles of cDNA amplification
(Spearman r= 0.89 ,Pearson r= 0.96
y= 0.94 x -0.18)
21 PCR cycles of cDNA amplification
(Spearman r= 0.73 ,Pearson r= 0.91
y= 0.86 x -0.3)

Supplementary Figure S4. Estimation of ERCC transcript abundance with the Illumina HiSeq 2500 or MiSeq platform. The FPKM method (A, B) or molecular counting of the 5’ end fragments (C, D) or the 3’ end fragments (E, F) of the cDNA molecules were used to estimate the ERCC cDNA abundance. Two cDNA amplification conditions are shown that involve either 14 or 21 cycles of PCR. Both the raw values (A, C, E) and the log10 transformed values are presented (B, D, F). The correlation coefficients for the raw values are as follows: In Picture (A) for the cDNA counts from the 14 PCR cycles against the Ambion RNA counts ($r_{pearson}$=0.72 , $r_{spearman}$=0.94) and for the cDNA counts from the 21 PCR cycles against the Ambion RNA counts ($r_{pearson}$=0.46 , $r_{spearman}$=0.89). In Picture (C) for the cDNA counts from the 14 PCR cycles against the Ambion RNA counts ($r_{pearson}$=0.89 , $r_{spearman}$=0.94) and for the cDNA counts from the 21 PCR cycles against the Ambion RNA counts ($r_{pearson}$=0.81 , $r_{spearman}$=0.86). In Picture (E) for the cDNA counts from the 14 PCR cycles against the Ambion RNA counts ($r_{pearson}$=0.88 , $r_{spearman}$=0.89) and for the cDNA counts from the 21 PCR cycles against the Ambion RNA counts ($r_{pearson}$=0.74 , $r_{spearman}$=0.73).

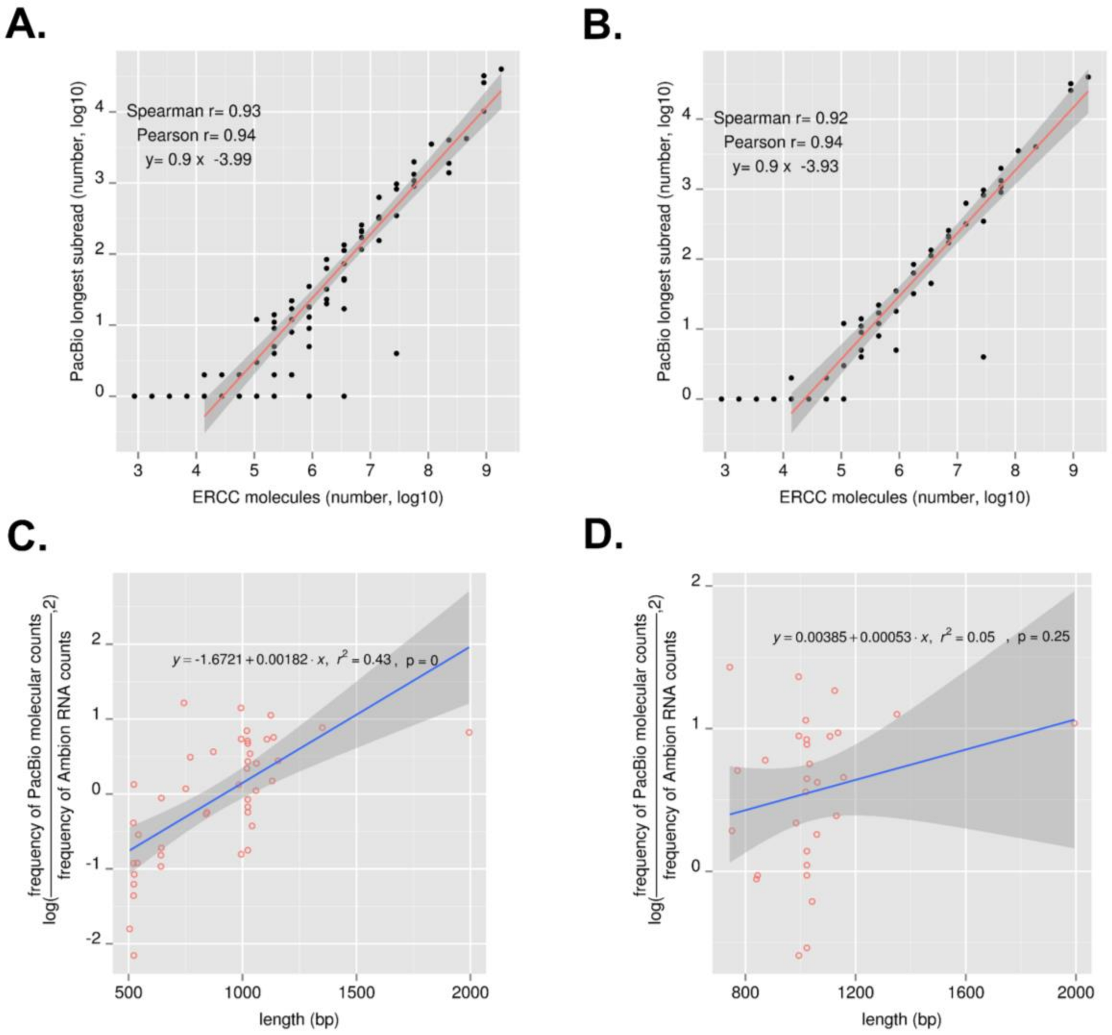

Supplementary Figure S5. Comparison of the ERCC cDNA abundance between the PacBio RS II platform against the expected number of RNA molecules as provided from the manufacturer (Ambion). In (A) all the ERCC transcripts are presented whereas in (B) only ERCC transcripts with more than 700 bp in length are presented. The total number of molecules presented on the x-axis of (A) and (B) figures corresponds to 3.5 pgs of ERCC RNA. C, D) Effect of the ERCC length on the estimation of the ERCC cDNA abundance with the PacBio RS II platform. The figures present deviations of the ERCC expression level estimates

with the PacBio RS II platform from the Ambion RNA molecular counts as a function of the ERCC length. In (C) all the ERCC transcripts are presented whereas in (D) only ERCC transcripts with more than 700 bp in length are presented. We plot the log2 ratio of observed (PacBio RS II) to expected (Ambion) read counts for the ERCC spike-ins (y-axis, log) for each of the samples relative to their length (x-axis). Each point has at least 5 sequenced reads in the PacBio RS II run.

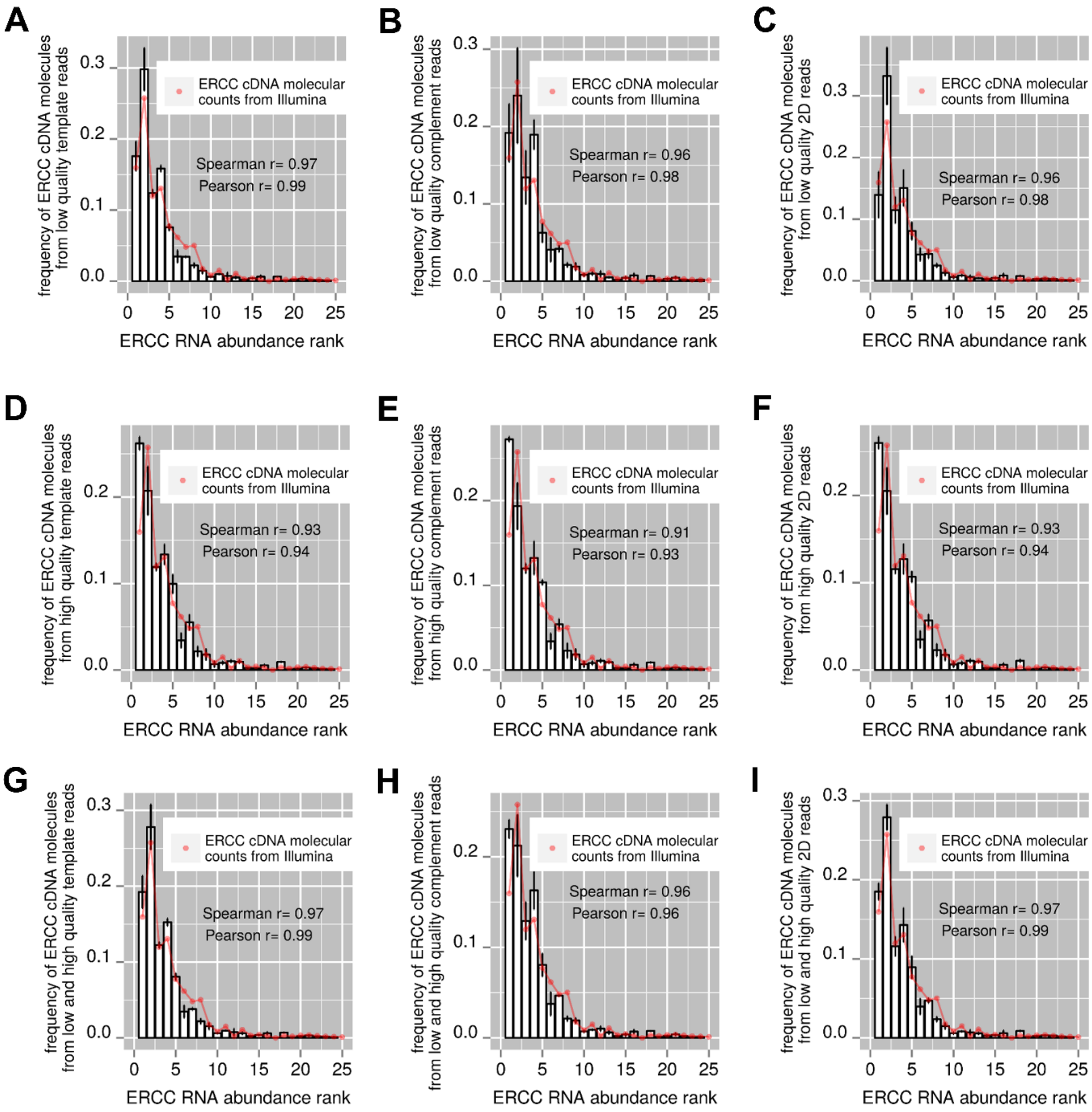

Supplementary Figure S6. cDNA abundance of the 92 ERCC transcripts from the ONT MinION sequencing (barplots). The expected cDNA abundance of the ERCC transcripts as calculated with the Illumina 5’ end molecular counts is also presented (red lines). The data shown are derived from the ERCC sample with the 14 PCR cycles of cDNA amplification reaction. A different plot is presented for each one of the three MinION read groups template (A, D, G), complement (B, E,

H), 2D reads (C, F, I) for both the low (A-C) and the high (D-F) quality categories as well as both low and high quality reads together (G-I). The height of the vertical bars represent the average value from the MinION experiments number 2, 3, 4. The standard deviation has been calculated accordingly. The correlation between the observed cDNA abundance from the ONT MinION and the expected cDNA abundance from Illumina is also shown on the graph. The ERCC transcripts are sorted from the most abundant (on the left of the x axis) to the least abundant (on the right of the x axis) based on the RNA concentration as provided from the manufacturer (Ambion). The 25 most abundant ERCC transcripts are presented.

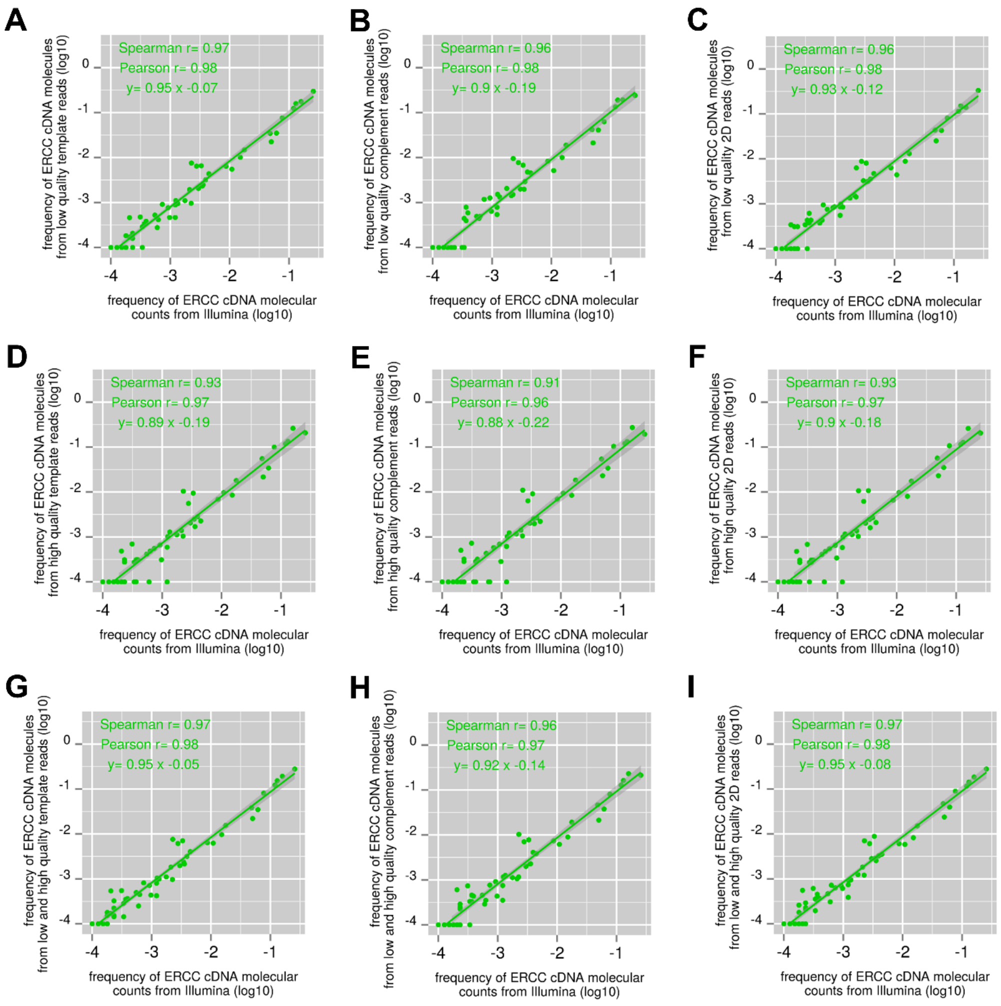

Supplementary Figure S7. Similar with Supplementary Fig. S6 but the values are log10 transformed. For the y-axis values only the average value from the MinION experiments number 2, 3, 4 for the corresponding ERCC transcript is used. For the x-axis the corresponding Illumina 5' end molecular counts value is used. All the ERCC transcripts with sequenced Illumina 5' end fragments or MinION reads or both, are presented.

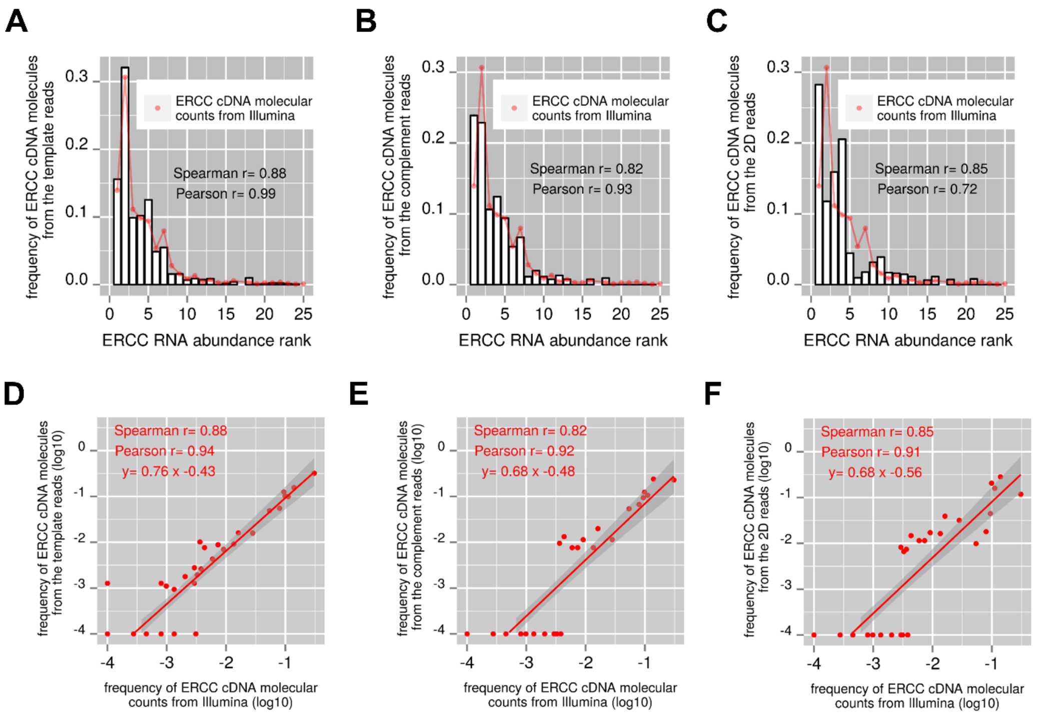

Supplementary Figure S8. A-C) cDNA abundance of the 92 ERCC transcripts from the ONT MinION sequencing (barplots). The expected cDNA abundance of the ERCC transcripts as calculated with the Illumina 5' end molecular counts is also presented (red lines). The data shown are derived from the ERCC sample with the 21 PCR cycles of cDNA amplification reaction. The correlation between the observed cDNA abundance from the ONT MinION and the expected cDNA abundance from Illumina is shown on the graph. The ERCC transcripts are sorted from the most abundant (on the left of the x axis) to the least abundant (on the right of the x axis) based on the RNA concentration as provided from the manufacturer (Ambion). The 25 most abundant ERCC transcripts are presented. D-F) Similar with (A-C) but the values are log10 transformed. For the y-axis values the value from the MinION experiment is used. For the x-axis values the corresponding

Illumina 5’ end molecular counts value is used. A different plot is presented for each one of the three MinION read groups template (A, D), complement (B, E), 2D reads (C, F). All the ERCC transcripts with sequenced Illumina 5’ end fragments or MinION reads or both, are presented.

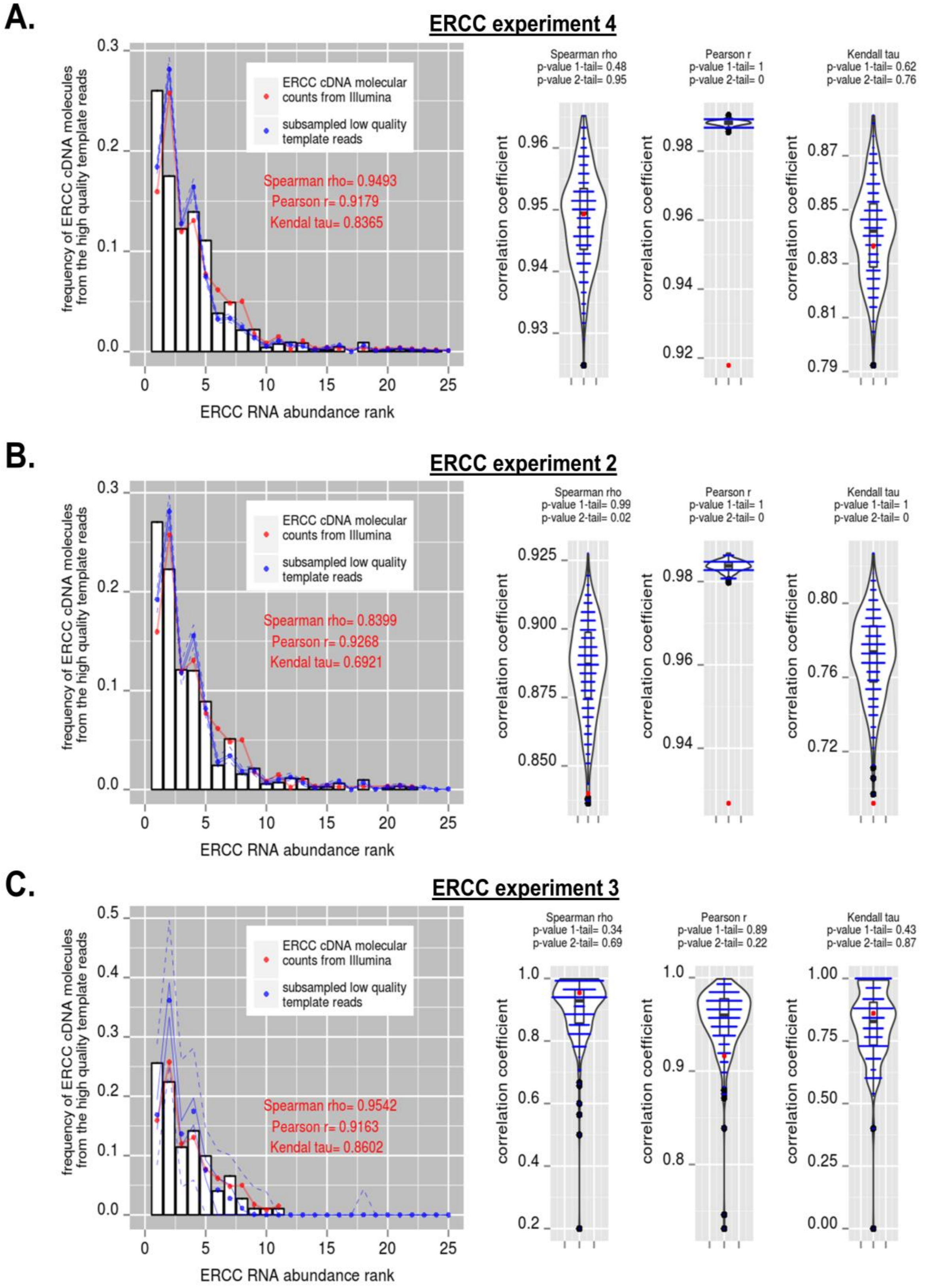

(the figure continues on the next page)

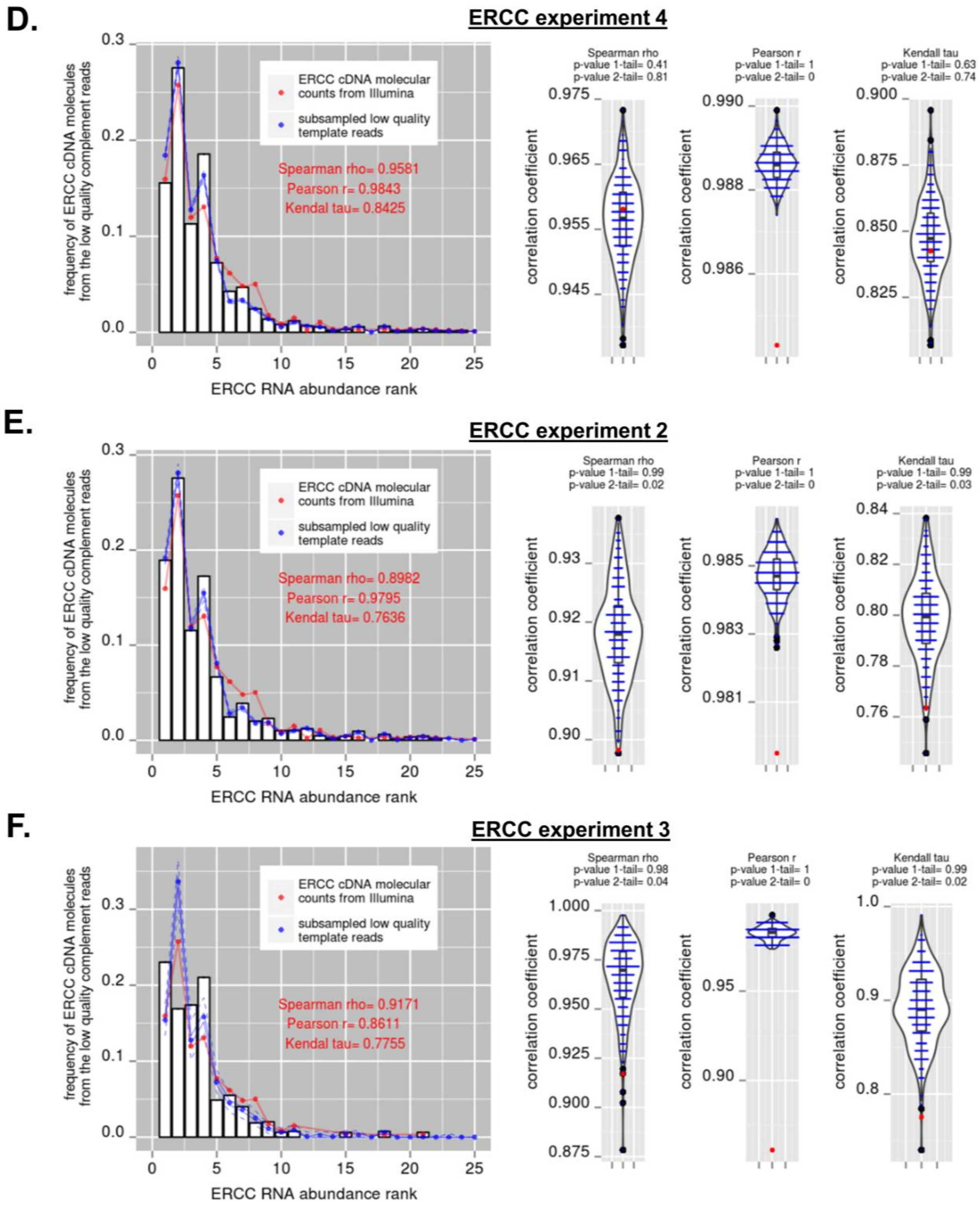

(the figure continues on the next page)

G.

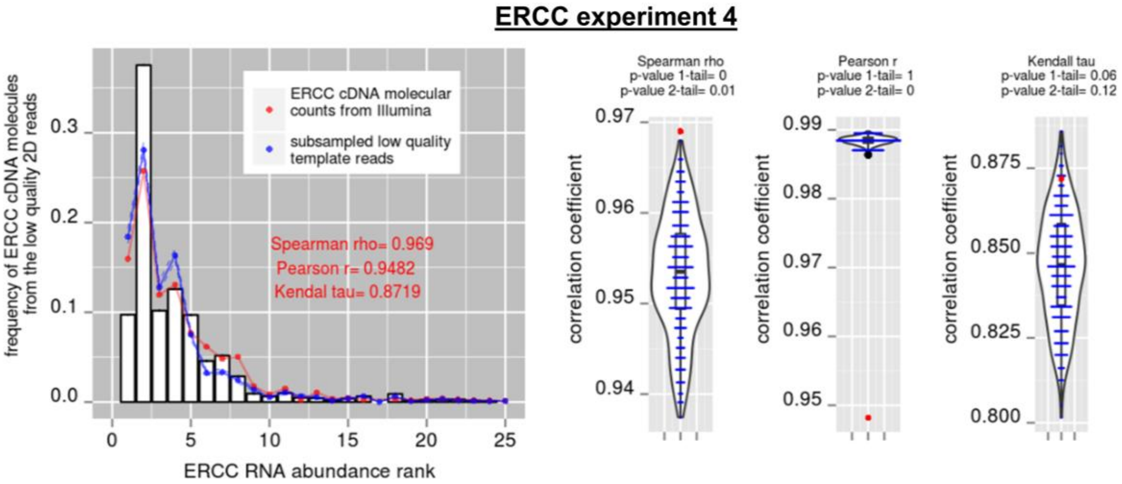
ERCC experiment 4
frequency of ERCC cDNA molecules from the low quality 2D reads
ERCC cDNA molecular counts from Illumina
subsampled low quality template reads
Spearman rho= 0.969
Pearson r= 0.9482
Kendal tau= 0.8719
ERCC RNA abundance rank
Spearman rho
p-value 1-tail= 0
p-value 2-tail= 0.01
Pearson r
p-value 1-tail= 1
p-value 2-tail= 0
Kendall tau
p-value 1-tail= 0.06
p-value 2-tail= 0.12
correlation coefficient

H.

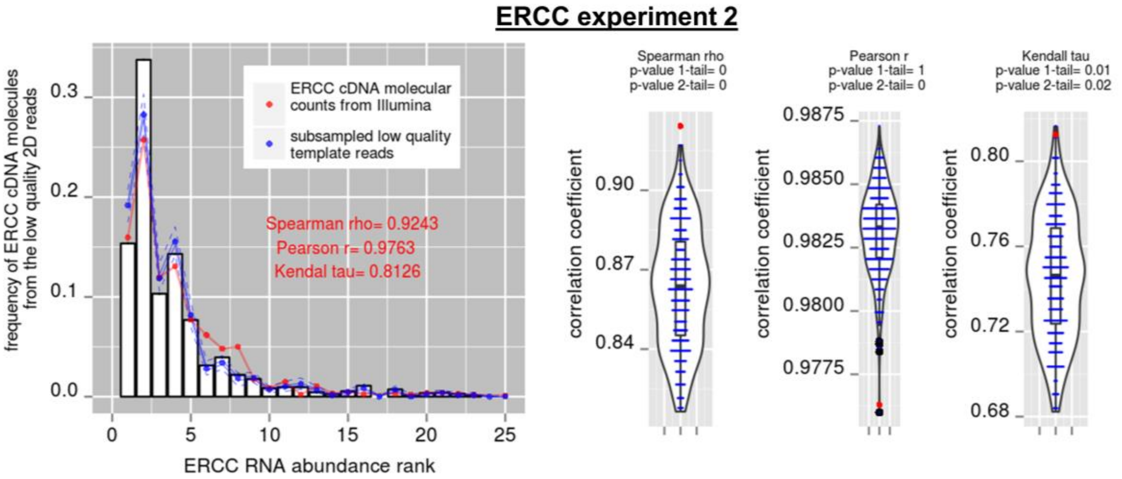
ERCC experiment 2
frequency of ERCC cDNA molecules from the low quality 2D reads
ERCC cDNA molecular counts from Illumina
subsampled low quality template reads
Spearman rho= 0.9243
Pearson r= 0.9763
Kendal tau= 0.8126
ERCC RNA abundance rank
Spearman rho
p-value 1-tail= 0
p-value 2-tail= 0
Pearson r
p-value 1-tail= 1
p-value 2-tail= 0
Kendall tau
p-value 1-tail= 0.01
p-value 2-tail= 0.02
correlation coefficient

I.

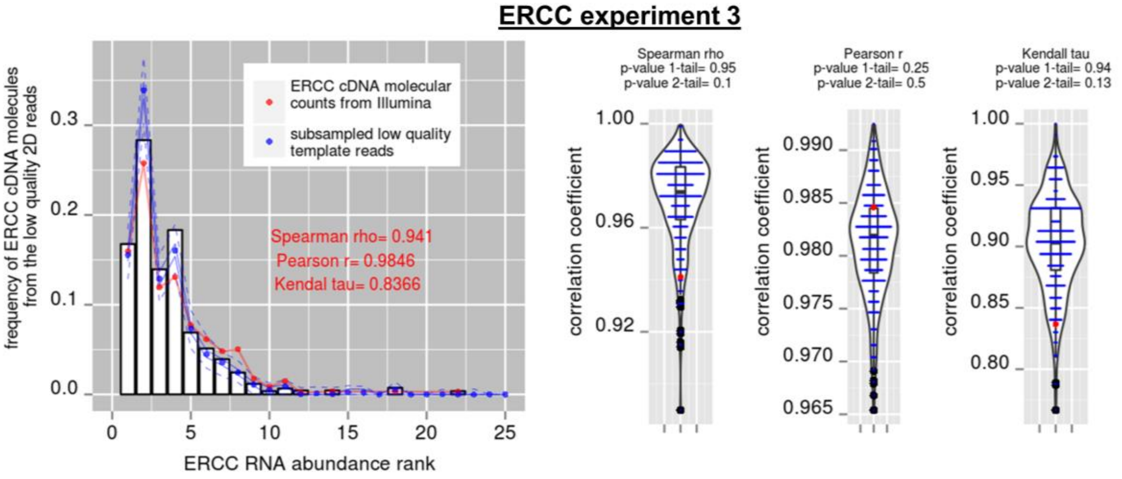
ERCC experiment 3
frequency of ERCC cDNA molecules from the low quality 2D reads
ERCC cDNA molecular counts from Illumina
subsampled low quality template reads
Spearman rho= 0.941
Pearson r= 0.9846
Kendal tau= 0.8366
ERCC RNA abundance rank
Spearman rho
p-value 1-tail= 0.95
p-value 2-tail= 0.1
Pearson r
p-value 1-tail= 0.25
p-value 2-tail= 0.5
Kendall tau
p-value 1-tail= 0.94
p-value 2-tail= 0.13
correlation coefficient

Supplementary Figure S9 (previous page). Consistency of comparisons between the low quality template reads against either the high quality template reads (A-C), the low quality complement reads (D-F) and the low quality 2D reads (G-I) for the ERCC experiments number 2 (B, E, H), number 3 (C, F, I) and number 4 (A, D, G). Each figure consists of a barplot on the left and three violin plots on the right. The bars on the barplot correspond to the ERCC transcript frequency for the reads presented on the y axis. The blue dots, on the barplots, correspond to the median value of the frequency for the individual ERCC transcripts from the 300 groups of the subsampled low quality template reads. The lower and upper solid blue lines, on the barplots, correspond to the frequency values present at the 25th and 75th percentile respectively from the distribution of the values from the 300 groups of the subsampled low quality template reads. The lower and upper dotted blue lines, on the barplots, correspond to the minimum and maximum frequency values from the distribution of the values from the 300 groups of the subsampled low quality template reads. The blue dots on the violin plots show the distribution of the Spearman, Pearson and Kendall correlation coefficients for the comparisons of each one of the 300 subsampled groups against the Illumina molecular counts (the blue lines on the violin plots are the horizontal pileup of these dots), along with their distribution density. The distribution of the correlation coefficient values is also indicated as boxplots on the violin plots. The correlation coefficient values under testing (we call them sample correlations), which indicate the agreement between the height of the bars and the red line on

the barplots, are presented with red letters on the barplots and as red dots on the violin plots. The 1-tail and 2-tail p-values correspond to the probability of the sample correlation values from a gamma distribution fitted on the distribution of the correlation coefficient values from the 300 subsampled group comparisons.

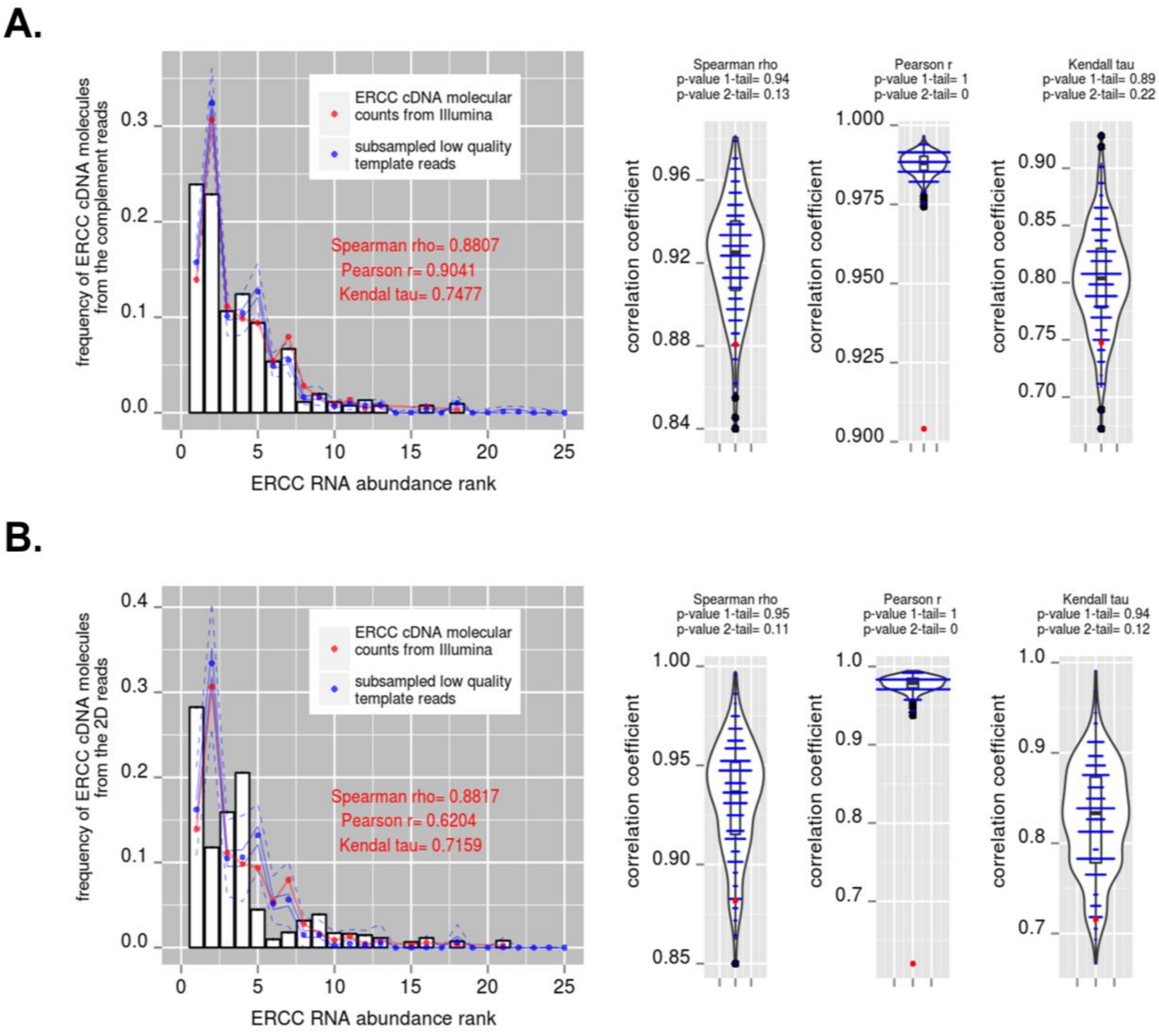

Supplementary Figure S10. Consistency of comparisons between the template reads against either the complement reads (A) or the 2D reads (B) for the ERCC experiment number 1. Each figure consists of a barplot on the left and three violin plots on the right. The bars on the barplot correspond to the ERCC transcript frequency for the reads presented on the y axis. The blue dots, on the

barplots, represent the average value of the frequency for the individual ERCC transcripts from the 300 groups of the subsampled template reads. The lower and upper solid blue lines, on the barplots, correspond to the frequency values present at the 25th and 75th percentile respectively from the distribution of the values from the 300 groups of the subsampled template reads. The lower and upper dotted blue lines, on the barplots, correspond to the minimum and maximum frequency values from the distribution of the values from the 300 groups of the subsampled template reads. The blue dots on the violin plots show the distribution of the Spearman, Pearson and Kendall correlation coefficients for the comparisons of each one of the 300 subsampled groups against the Illumina molecular counts (the blue lines on the violin plots are the horizontal pileup of these dots), along with their distribution density. The distribution of the correlation coefficient values is also indicated as boxplots on the violin plots. The correlation coefficient values under testing (we call them sample correlations), which indicate the agreement between the height of the bars and the red line on the barplots, are presented with red letters on the barplots and as red dots on the violin plots. The 1-tail and 2-tail p-values correspond to the probability of the sample correlation values from a gamma distribution fitted on the distribution of the correlation coefficient values from the 300 subsampled group comparisons.

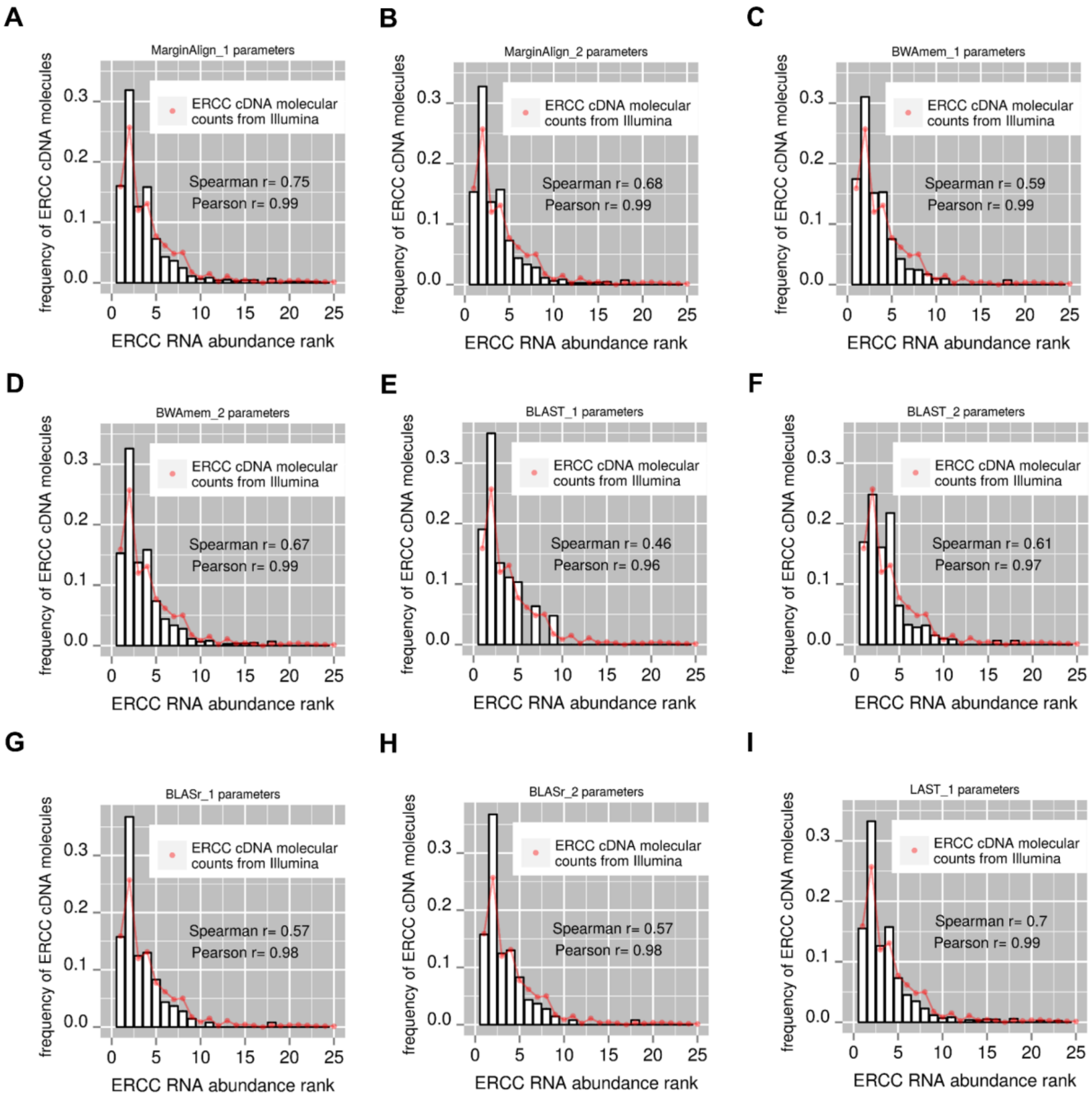

(the figure continues on the next page)

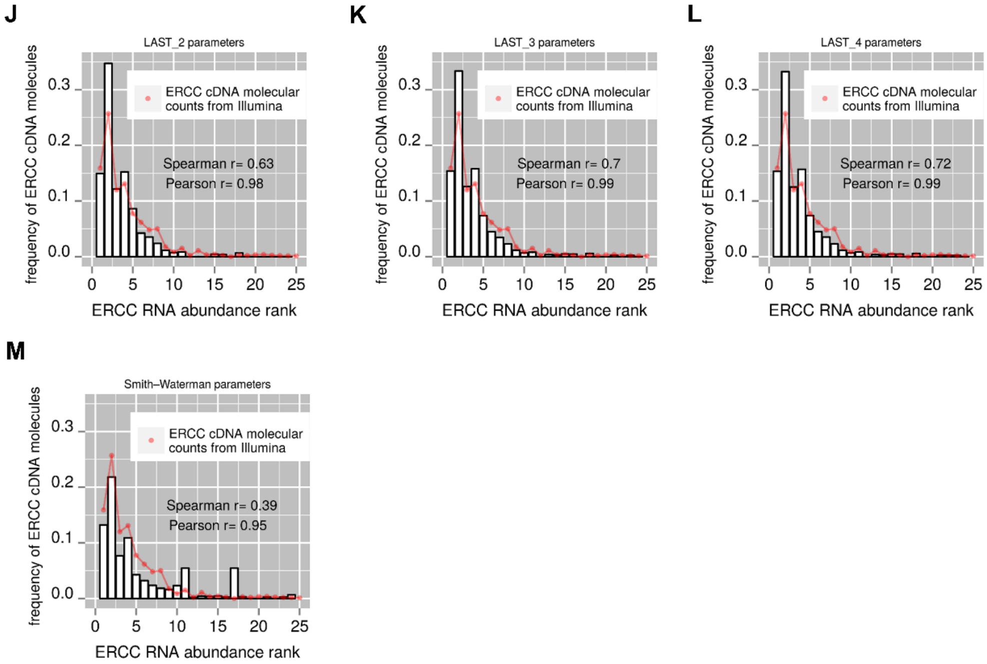

Supplementary Figure S11. cDNA abundance of the 92 ERCC transcripts from the ONT MinION sequencing (barplots) using different aligners and different alignment parameters (Supplementary Table S2). The low quality template read group of the MinION reads from the ERCC experiment number 3 is used. The expected cDNA abundance of the ERCC transcripts as calculated with the Illumina 5' end molecular counts is also presented (red lines). The correlation between the observed cDNA abundance from the ONT MinION and the expected cDNA abundance from Illumina is also shown on the graph. The ERCC transcripts are sorted from the most abundant (on the left of the x axis) to the least abundant (on the right of the x axis) based on the RNA concentration as provided from the manufacturer (Ambion). The 25 most abundant ERCC transcripts are presented.

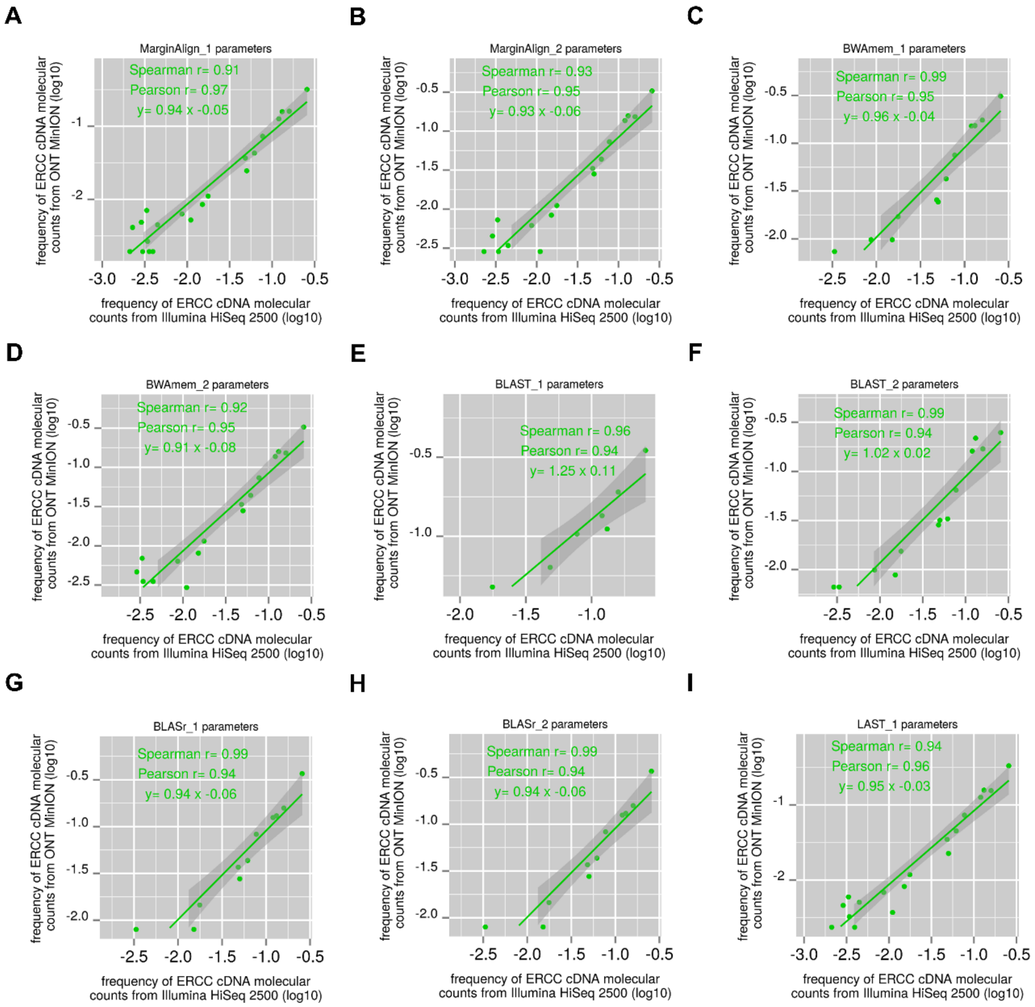

(the figure continues on the next page)

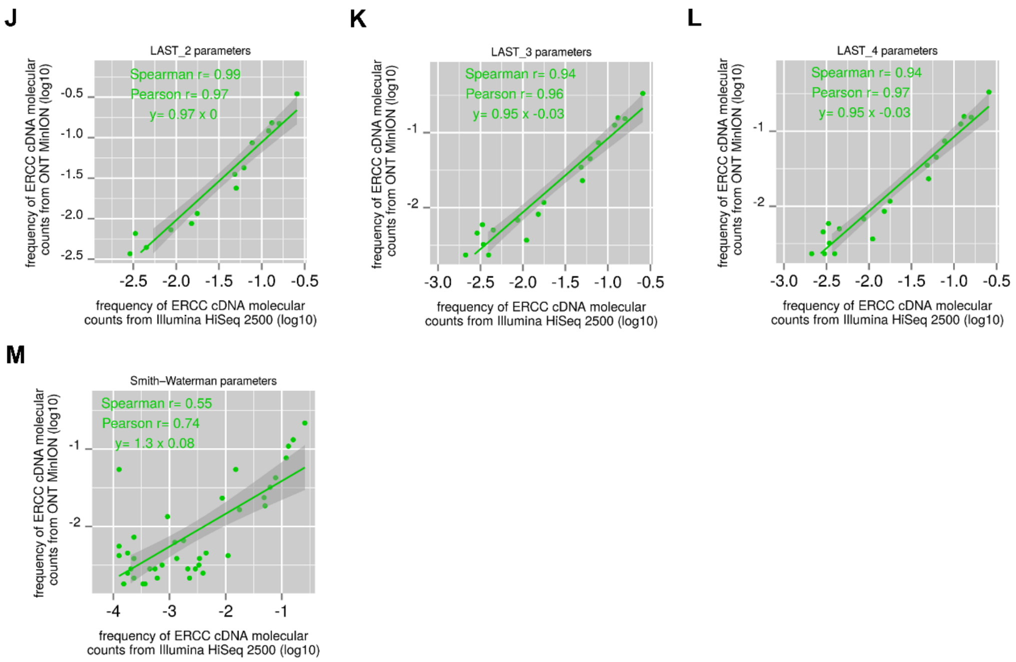

Supplementary Figure S12. Similar with Supplementary Fig. S11 but the values are log10 transformed. For the y-axis values the values from the MinION experiment number 3 were used. For the x-axis the corresponding Illumina 5' end molecular counts values were used. All the ERCC transcripts, with either Illumina sequenced reads or MinION sequenced molecules or both, are presented

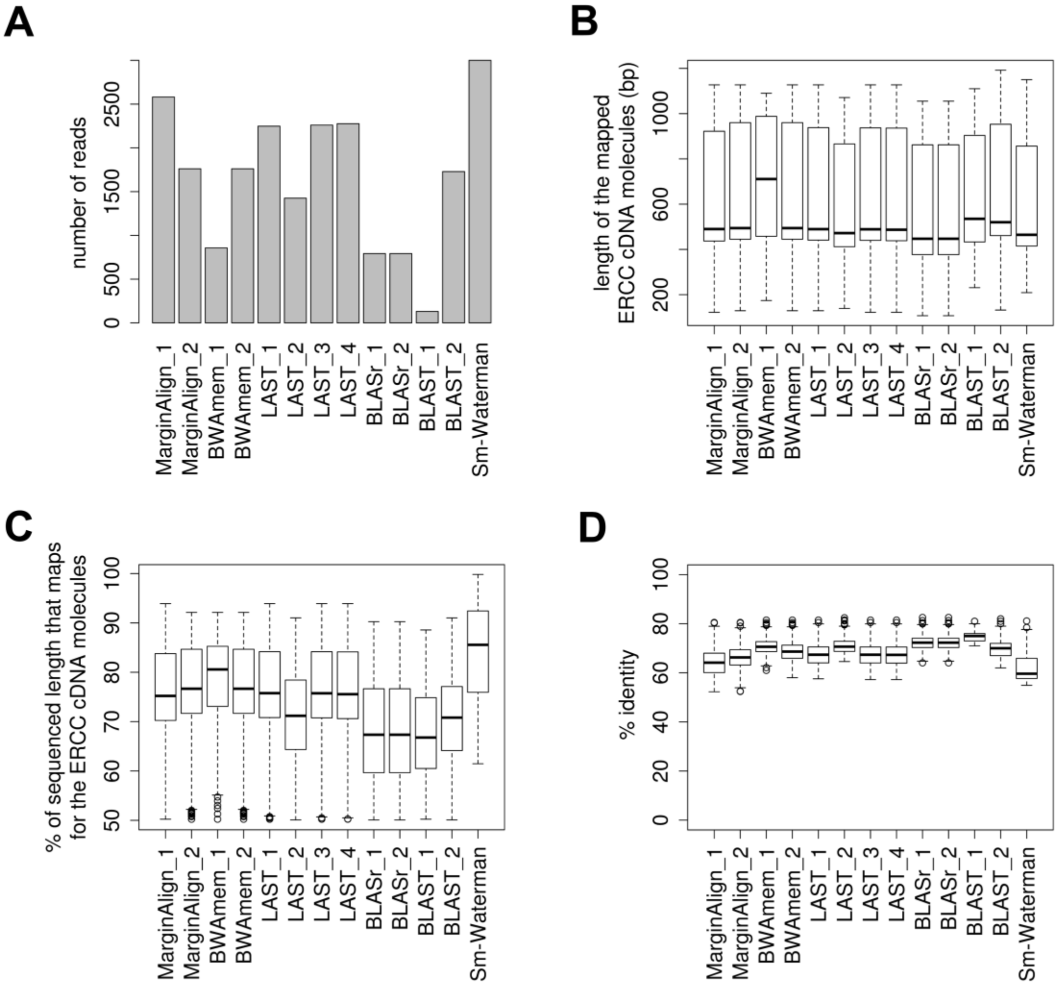

Supplementary Figure S13. Comparison of different aligners and alignment parameters*.* Number of aligned reads (A), raw alignment length (B), alignment length as percentage of the sequenced length (C), identity (D) for the different aligners and the different alignment parameters of Supplementary Table S2. The low quality template read group of the MinION reads from the ERCC experiment number 3 were used. The identity corresponds to the percentage of matches over the sum of matches, mismatches, insertions and deletions.

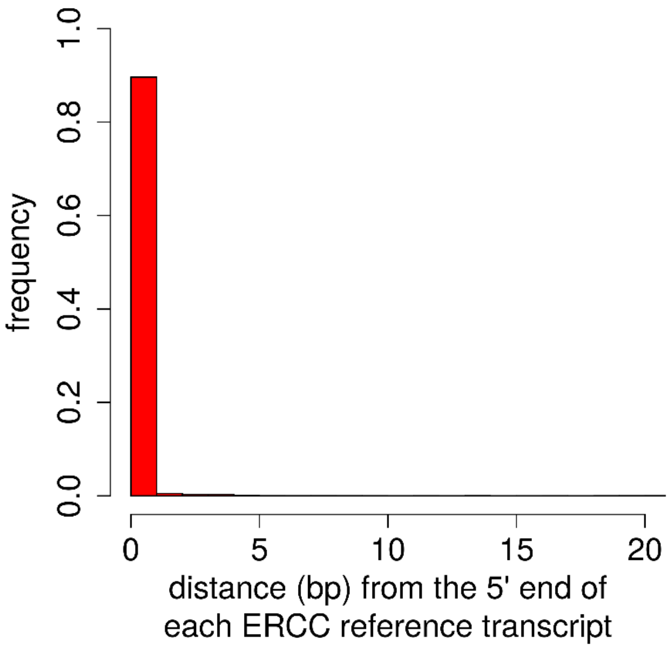

Supplementary Figure S14. Positional distribution on the ERCC reference transcripts of the Illumina HiSeq 2500 or MiSeq fragments. Only fragments that were derived from the 5’ end of the ERCC cDNA molecules are used. Illumina fragments from both the ERCC sample with the 21 PCR cycles of cDNA amplification and the ERCC sample with the 14 PCR cycles of cDNA amplification, are pooled together and are presented in the graph.

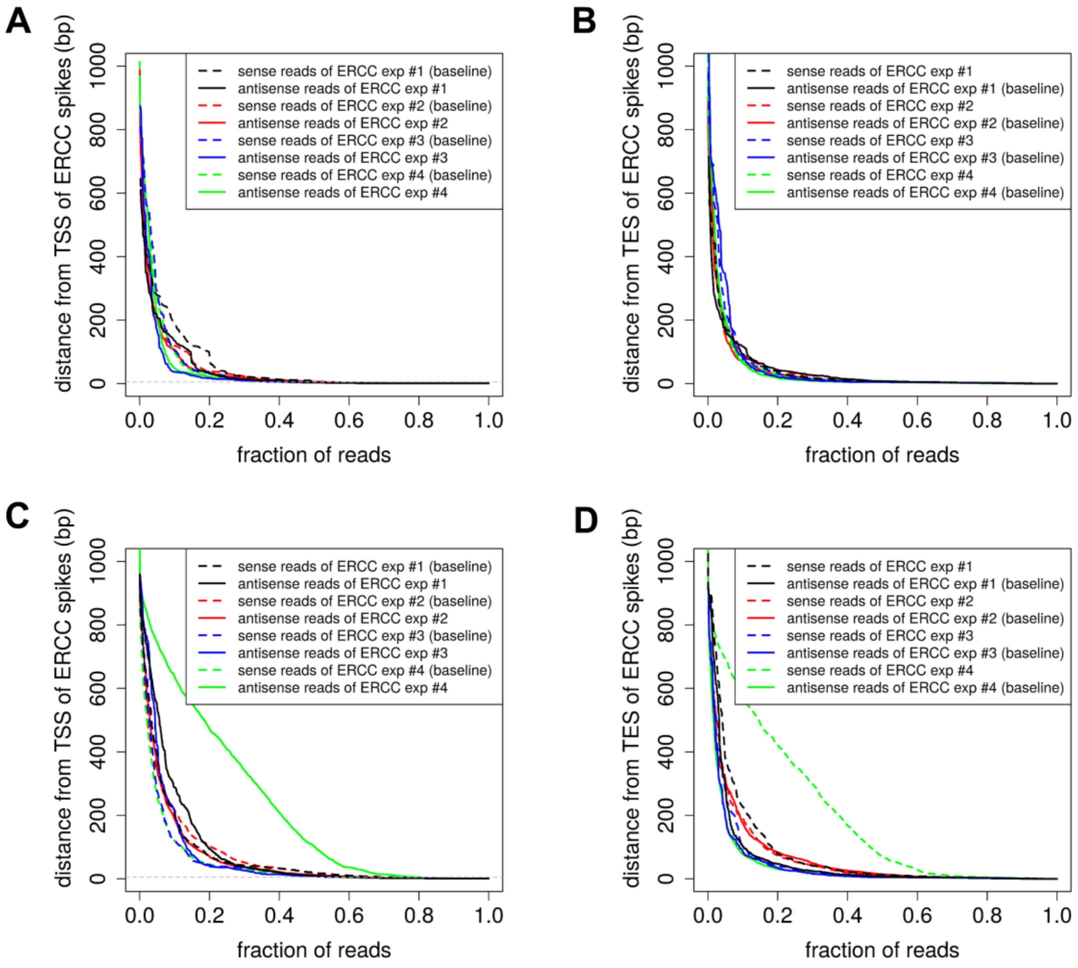

Supplementary Figure S15. Full length processivity of the ERCC cDNA molecules from the ONT MinION platform. Distance (number of nucleotides) from the transcription start site (TSS) or the transcription end site (TES) for the reads where the antisense strand (A, C) or the sense strand (B, D) were sequenced first respectively. The graphs correspond to two different read groups. In (A, B) the 2D read group (from both the high and low quality categories) is presented and in (C, D) the reads from the low quality template read group, that are not present in the low quality 2D read group, are presented. The reads are sorted from the ones that are further from the TSS or TES (left side of x axis) to

the closest to the TSS or TES (right side of x axis). In (A, C) the distance from the TSS of the 5’ end of the sense strand reads is used as a baseline reference indicating how well the aligner can align the ends of the MinION reads which should start from the zero position. Similarly in (B, D) the anti-sense strand reads are used as a baseline reference. In (A, C) the thin dashed horizontal blue line marks the position 5 bp from the TSS.

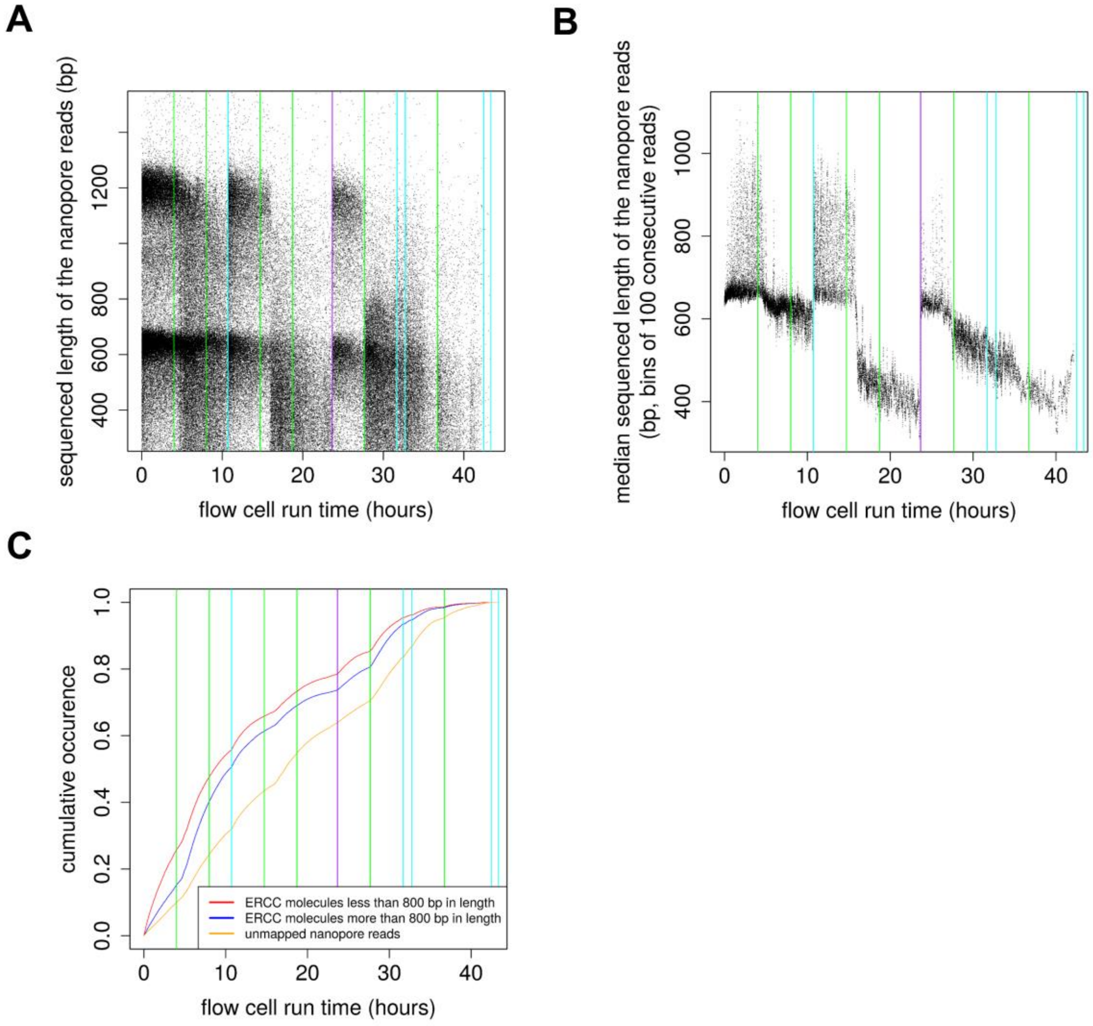

Supplementary Figure S16. Length of the ONT MinION reads over the sequencing time. A) Length of sequenced reads over time for the template read group of ERCC experiment number 4. Both low and high quality template reads are shown**.** B) Like figure (A) but the points correspond to the median length of 100 consecutive reads in overlapping windows. C) Cumulative occurrence of MinION reads aligned on ERCC molecules less (blue line) or more than 800 bp in length (red line). The green color of the vertical bars represent the time points where the voltage is increased by 5 mV relative to the previous time window (the

baseline voltage at every restart is 140mV[1]) .The cyan line represents the time point where the machine is restarted without the addition of new sequencing library. The purple line represents the time point where the machine is restarted with the addition of new sequencing library.

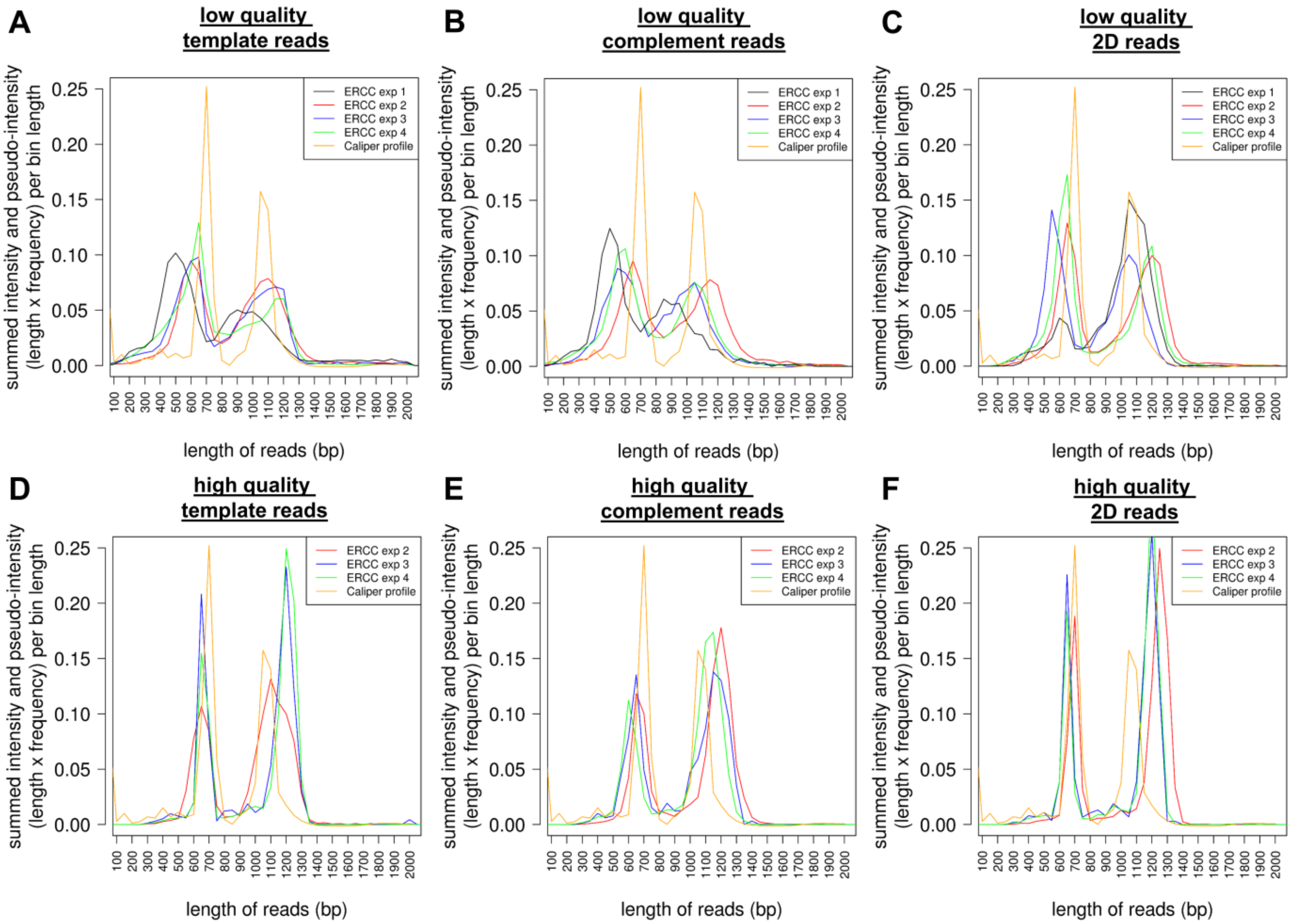

Supplementary Figure S17. Read length frequency distribution of the cDNA molecules sequenced on the ONT MinION platform from the ERCC experiments 1, 2, 3, 4. The reads from the low quality template (A), low quality complement (B) and low quality 2D (C) read categories are presented. The reads from the high quality template (D), high quality complement (E) and high quality 2D (F) read categories are also presented. The cDNA electrophoresis profile from the Caliper Labchip GX instrument for the ERCC cDNA library is also presented (yellow line). In order to compare the cDNA electrophoresis profile from Caliper

and the ONT MinION cDNA read length abundance we performed transformations on the data similar to the ones presented in Supplementary Fig. S24. For the ERCC experiment 1 the reads from the template, complement and 2D read categories are presented in (A), (B), (C) respectively. Although these reads are presented on the low quality category plots they correspond to reads derived from both high and low quality groups because the basecalling pipeline of the ERCC experiment 1 did not have the quality filter option.

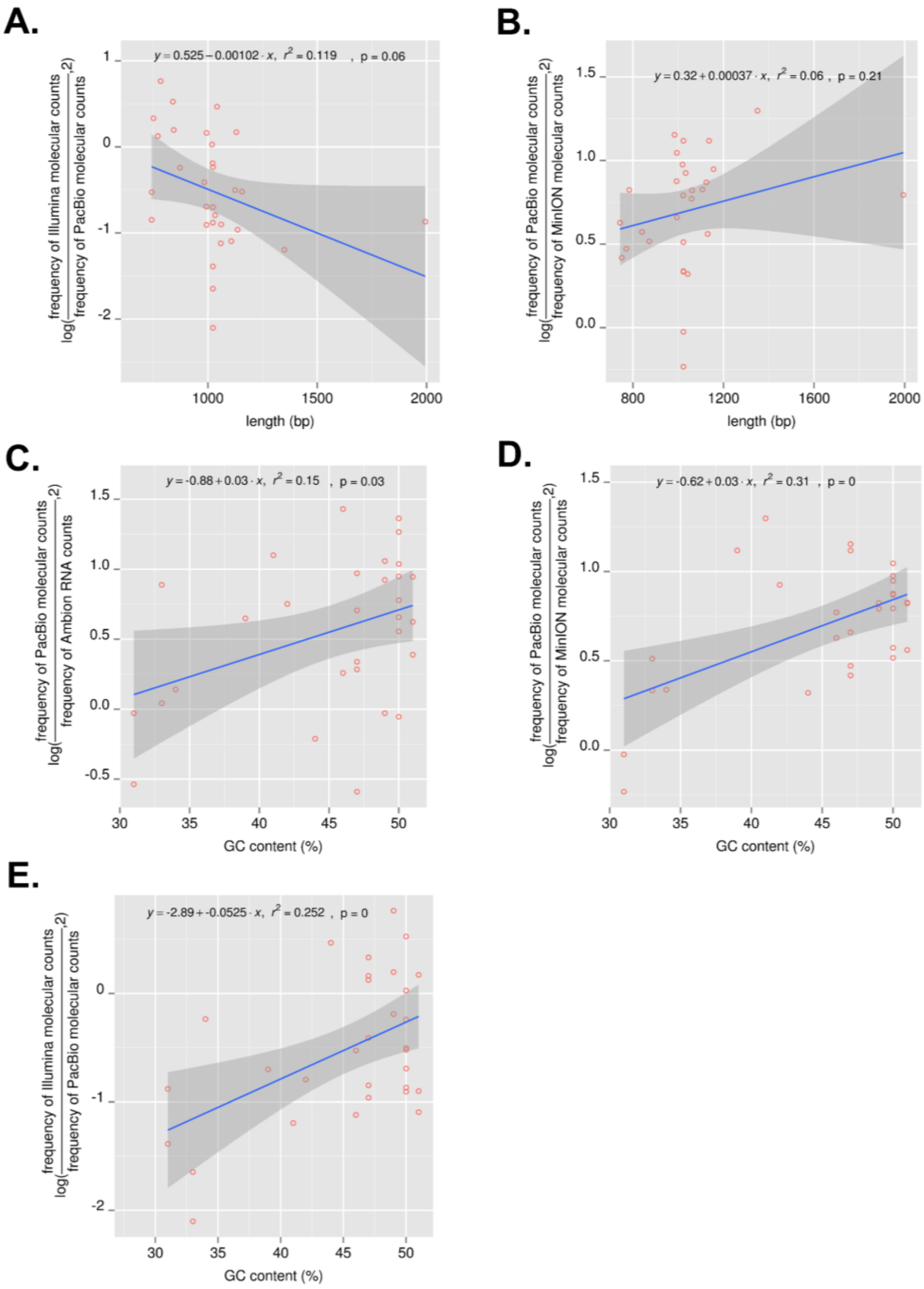

Supplementary Figure S18. Effect of the GC content and ERCC length on the estimation of the ERCC cDNA abundance with the PacBio RS II platform. The figures present deviations of the ERCC expression level estimates with the PacBio RS II platform from the Illumina HiSeq 2500/MiSeq estimated cDNA abundance

(A, E), from the ONT MinION estimated cDNA abundance (B, D) or from the Ambion RNA molecular counts (C) as a function of the ERCC length (A, B) and the GC content (C, D, E). Only ERCC transcripts with more than 700 bp in length and with at least 5 reads in the PacBio RS II run, are presented. We plot the log2 ratio of observed (PacBio RS II) to expected (Ambion, Illumina, ONT MinION) read counts for the ERCC spike-ins (y-axis, log) for each of the samples relative to their length or GC content (x-axis). For the PacBio RS II / ONT MinION comparison the low and high quality template reads from the ONT MinION ERCC experiment 4 are used.

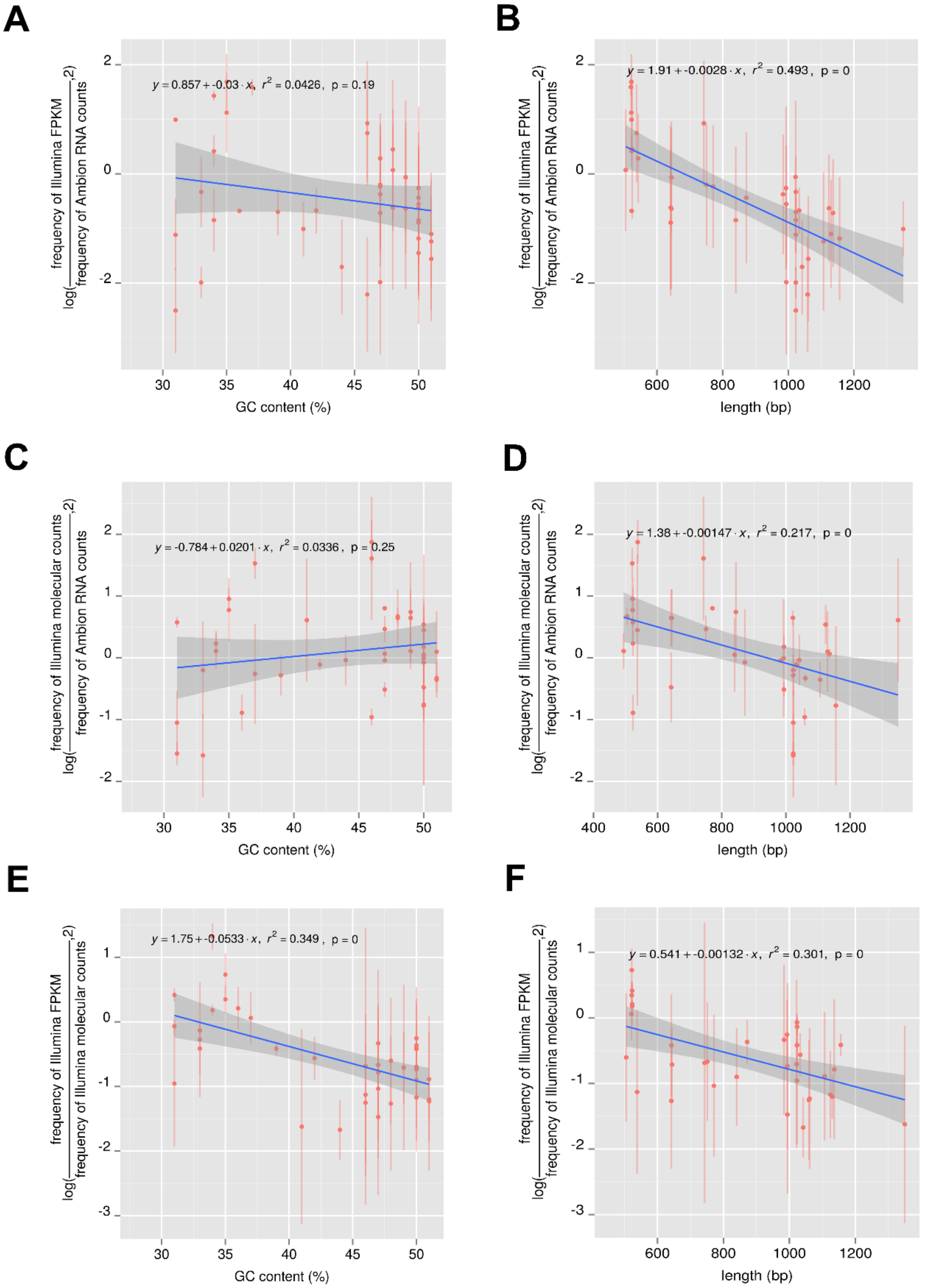
A
y = 0.857 + -0.03 · x, r2 = 0.0426, p = 0.19
log(frequency of Illumina FPKM / frequency of Ambion RNA counts, 2)
GC content (%)
B
y = 1.91 + -0.0028 · x, r2 = 0.493, p = 0
log(frequency of Illumina FPKM / frequency of Ambion RNA counts, 2)
length (bp)
C
y = -0.784 + 0.0201 · x, r2 = 0.0336, p = 0.25
log(frequency of Illumina molecular counts / frequency of Ambion RNA counts, 2)
GC content (%)
D
y = 1.38 + -0.00147 · x, r2 = 0.217, p = 0
log(frequency of Illumina molecular counts / frequency of Ambion RNA counts, 2)
length (bp)
E
y = 1.75 + -0.0533 · x, r2 = 0.349, p = 0
log(frequency of Illumina FPKM / frequency of Illumina molecular counts, 2)
GC content (%)
F
y = 0.541 + -0.00132 · x, r2 = 0.301, p = 0
log(frequency of Illumina FPKM / frequency of Illumina molecular counts, 2)
length (bp)

Supplementary Figure S19 (previous page). Deviation of the ERCC expression level estimates with the Illumina HiSeq 2500 or MiSeq platforms from the expected Ambion RNA molecular counts as a function of the GC content (A, C, E) and the ERCC length (B, D, F). A-D) We plot the log2 ratio of observed cDNA abundance (Illumina) to the expected (Ambion) read counts for the ERCC spike-ins (y-axis, log) relative to their length or GC content (x-axis). The ERCC expression level is calculated with either the FPKM method or the 5' end fragments molecular counting. E, F) Comparison of the deviation in the ERCC expression level estimation between the FPKM method and the 5' end fragments molecular counting for the Illumina platform. We plot the log2 ratio of the cDNA abundance from the FPKM method to the cDNA abundance from the 5' end fragments molecular counting for the ERCC spike-ins (y-axis, log) relative to their length or GC content (x-axis). The biases in ERCC expression level estimation introduced with the FPKM method, relative to the 5' end fragment molecular counting, is indicated by the underrepresentation of ERCC transcripts with high GC content or underrepresentation of long ERCC transcripts. In all the graphs the points are derived from the average of the ratio values from the ERCC samples with either the 14 PCR cycles or the 21 PCR cycles of cDNA amplification, for each one of the ERCC transcripts. The standard deviation is calculated accordingly.

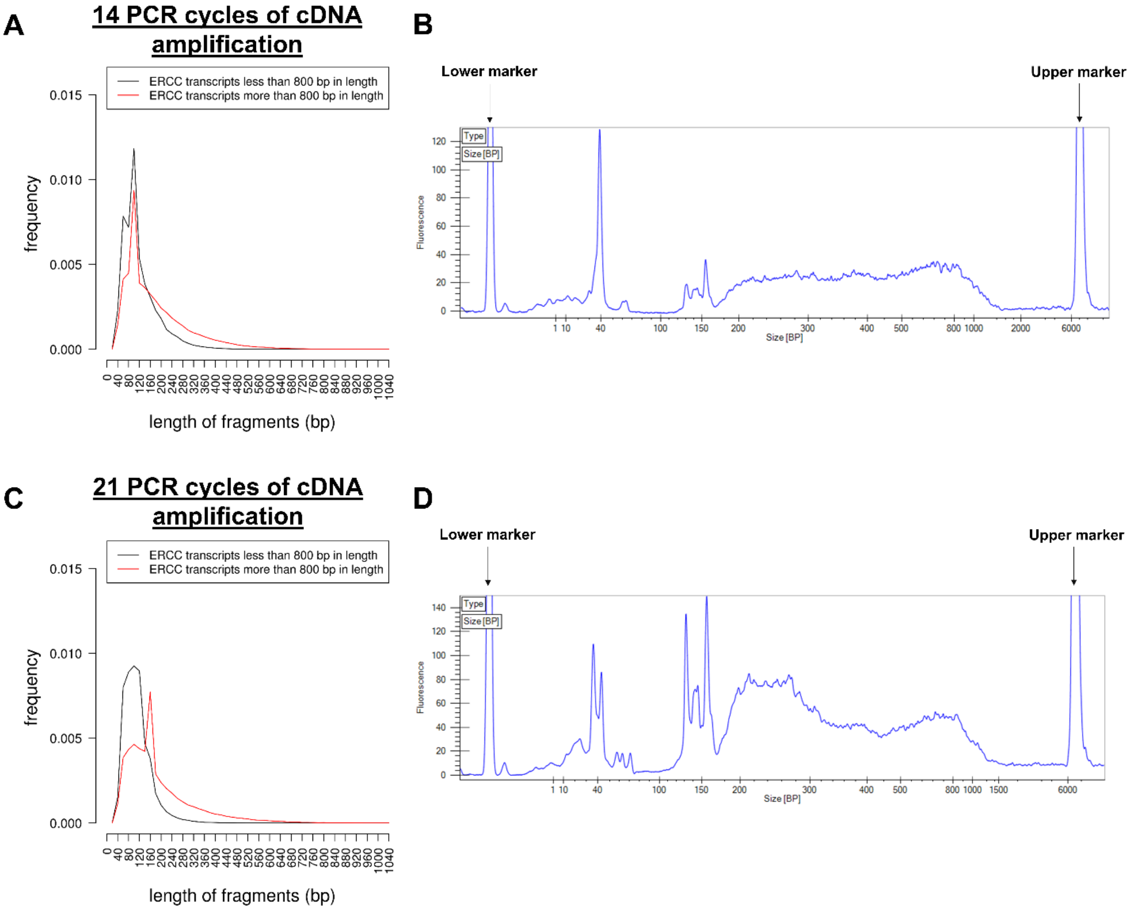

Supplementary Figure S20. Fragment size distribution of the Illumina libraries. The libraries of the 14 (A) and 21 (B) PCR cycles of cDNA amplification are presented. Two differently colored curves are shown in each graph. The black one corresponds to fragments derived from ERCC transcripts with length less than 800 bp. The red one corresponds to fragments derived from ERCC transcripts with length more than 800 bp. In (B) and (D) we are presenting the Caliper Labchip GX profile of the tagmentation fragments for the libraries derived from the 14 and 21 PCR cycles of cDNA amplification respectively. The tagmentation profile is the one exactly after the tagmentation amplification step and before the final Ampure XP cleanup step.

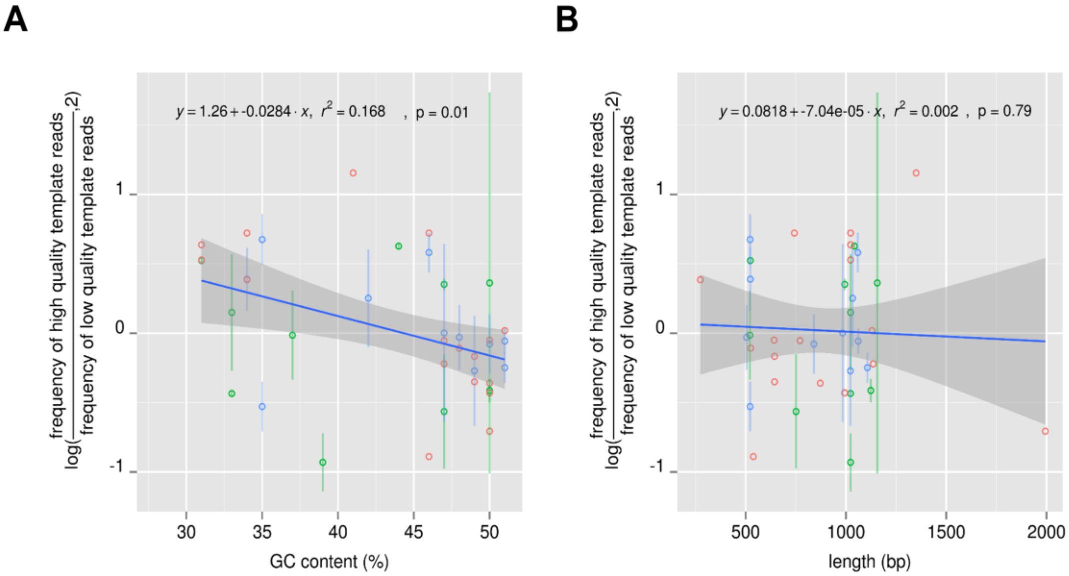

Supplementary Figure S21. Effect of GC content and length on the number of high quality reads acquired from each ERCC transcript. Only ERCC transcripts that have at least 5 high quality and at least 5 low quality reads are presented. We plot the log2 ratio of the frequency of high quality template reads relative to the frequency of low quality template reads (y-axis, log) for each ERCC transcript as a function of the GC content or length (x-axis). A regression line is presented with blue color along with its significance values.

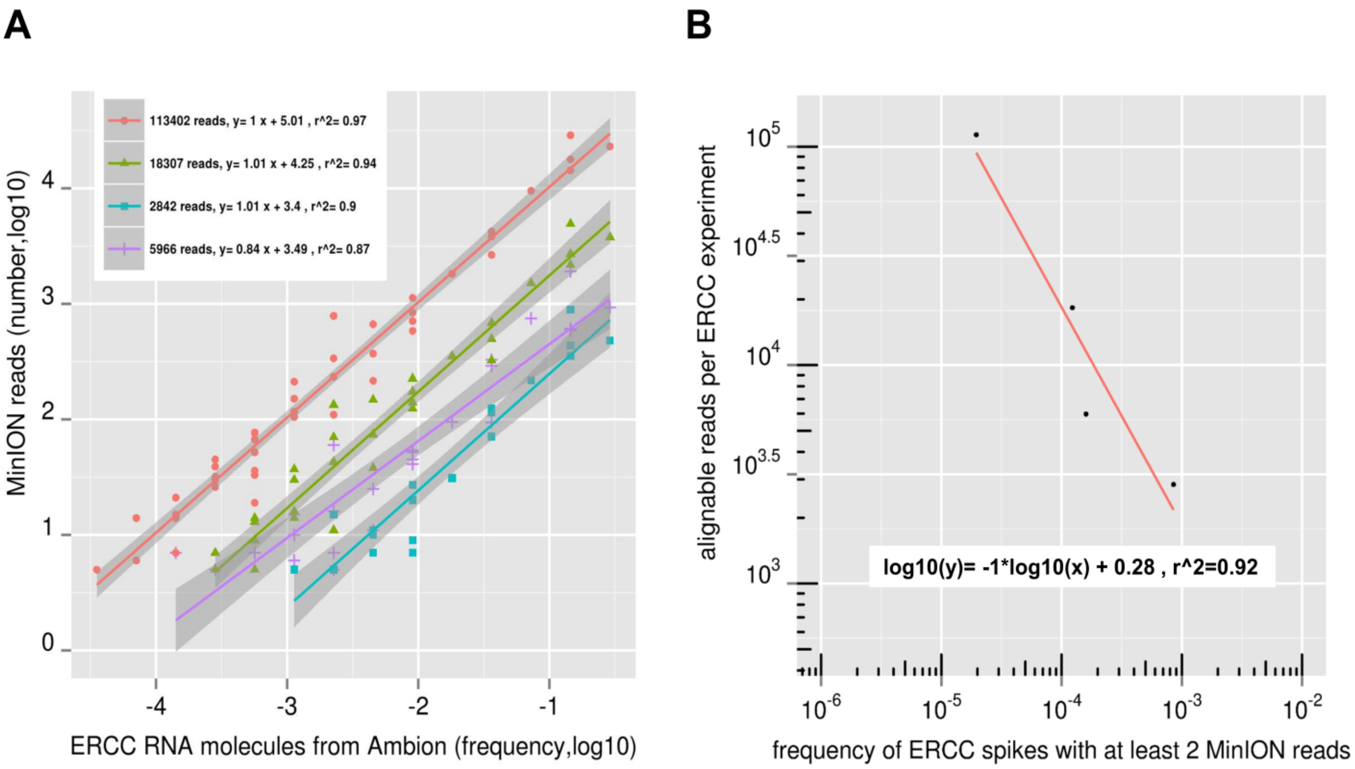

Supplementary Figure S22. Limit of detection of the ONT MinION platform as calculated from the ERCC experiments 1, 2, 3, 4. A) The number of MinION reads per ERCC transcript from the four ERCC experiments is presented relative to the expected frequency of the corresponding ERCC transcripts from Ambion. A linear regression line is fitted in the data from each experiment. The total number of aligned MinION reads per ERCC experiment along with the regression coefficient values and the correlation coefficient values are presented in the white box legend. The red, green, purple and cyan colored regression lines correspond to the ERCC experiments 4, 2, 1 and 3 respectively. B) Number of total aligned MinION reads obtained from the four ERCC experiments relative to the frequency of ERCC spikes with at least 2 MinION reads. The frequency of ERCC spikes with

at least 2 MinION reads was calculated from the regression line equations of picture (A). The number of total aligned MinION reads used are the ones presented in the white box legend of picture (A).

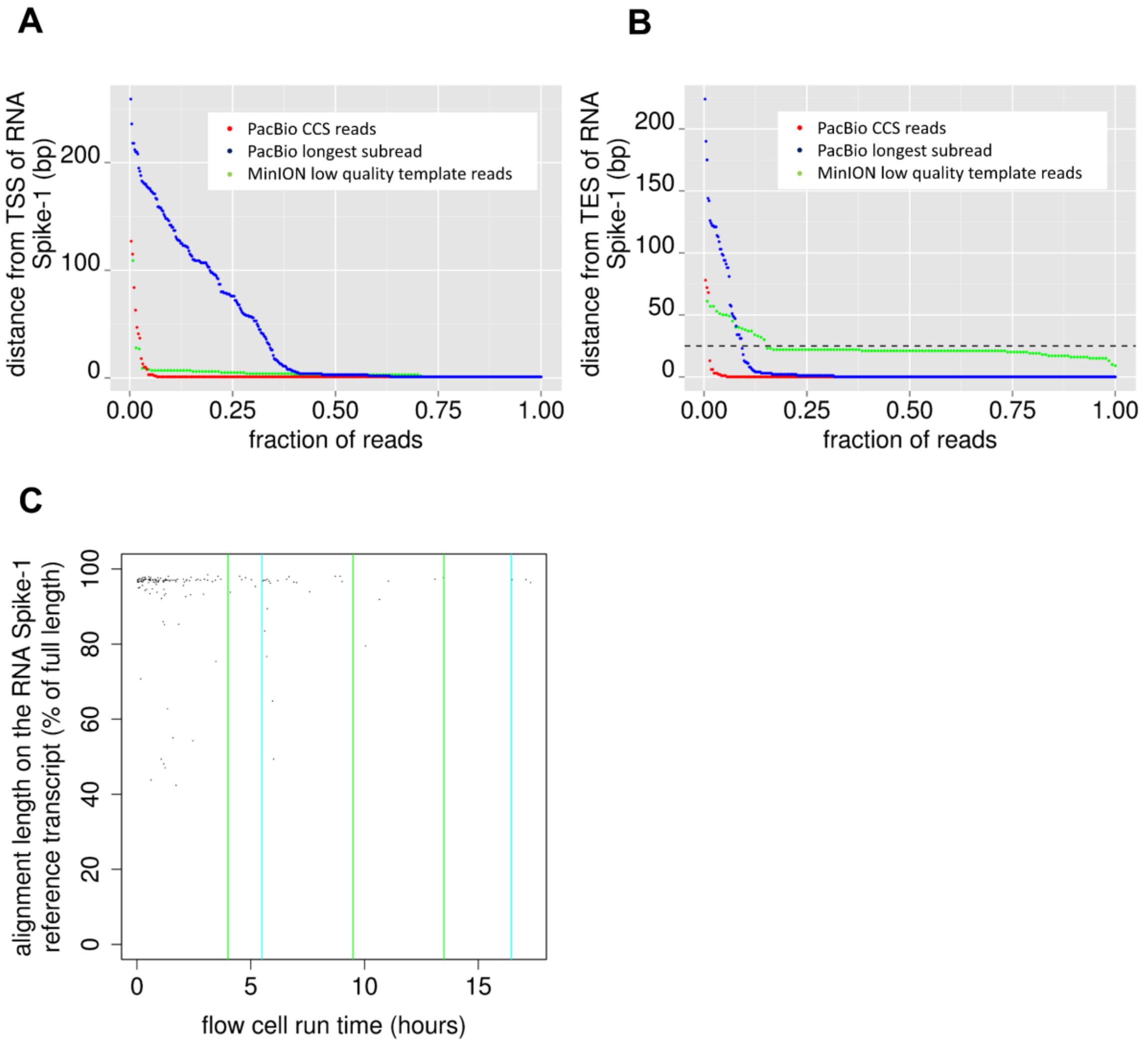

Supplementary Figure S23. Full length sequencing of the RNA Spike-1 cDNA molecules from the complex HEK-293 cDNA population. The distance of the sequenced reads from the TSS (A) or TES (B) are presented for the PacBio CSS reads, the PacBio longest subread and the ONT MinION low quality template reads. The reads are sorted from the ones that are further from the TSS or TES (left side of x axis) to the closest to the TSS or TES (right side of x axis). C) The full length processivity of the RNA Spike-1 cDNA molecules during the ONT MinION sequencing run is presented as sequenced fraction of the full length

sequence from the RNA Spike-1 reference transcript. In (B) the dashed horizontal black line marks the beginning position of the poly-A tail.

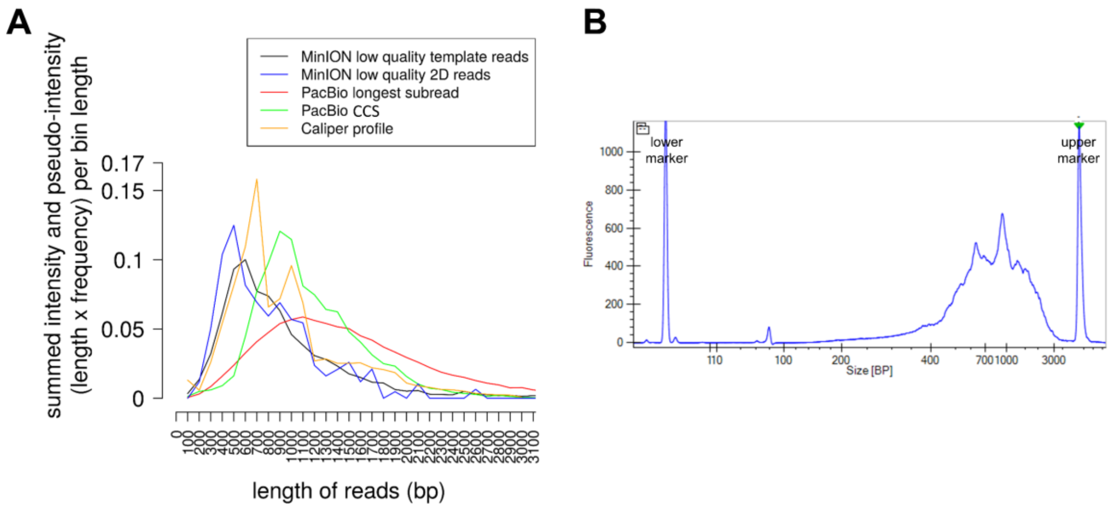

Supplementary Figure S24. Read length frequency distribution of the HEK-293 cDNA molecules sequenced on the ONT MinION and PacBio RS II platforms. A) For the ONT MinION platform the template and 2D read group from the low quality category are presented. For the PacBio RS II platform, cDNA reads derived from Circular Consensus Sequencing (CCS) are presented. Additionally, the longest subread is presented for molecules that were sequenced as CCS reads or not. The cDNA electrophoresis profile from the Caliper Labchip GX instrument is also presented (B). In order to compare the cDNA electrophoresis profile from the Caliper and the ONT MinION or the PacBIO RS II cDNA read length abundance we did the following transformations. In the case of the MinION reads we first binned the molecules based on their length in 100 bp intervals. Afterwards we multiplied the bin length value of the interval with the number of molecules in the specific bin and plotted the final values. We call these values as pseudo-intensity. In the case of the Caliper cDNA electrophoresis profile we binned the raw intensity

based on their corresponding length in 100 bp intervals and afterwards we summed the raw intensity values in each bin.

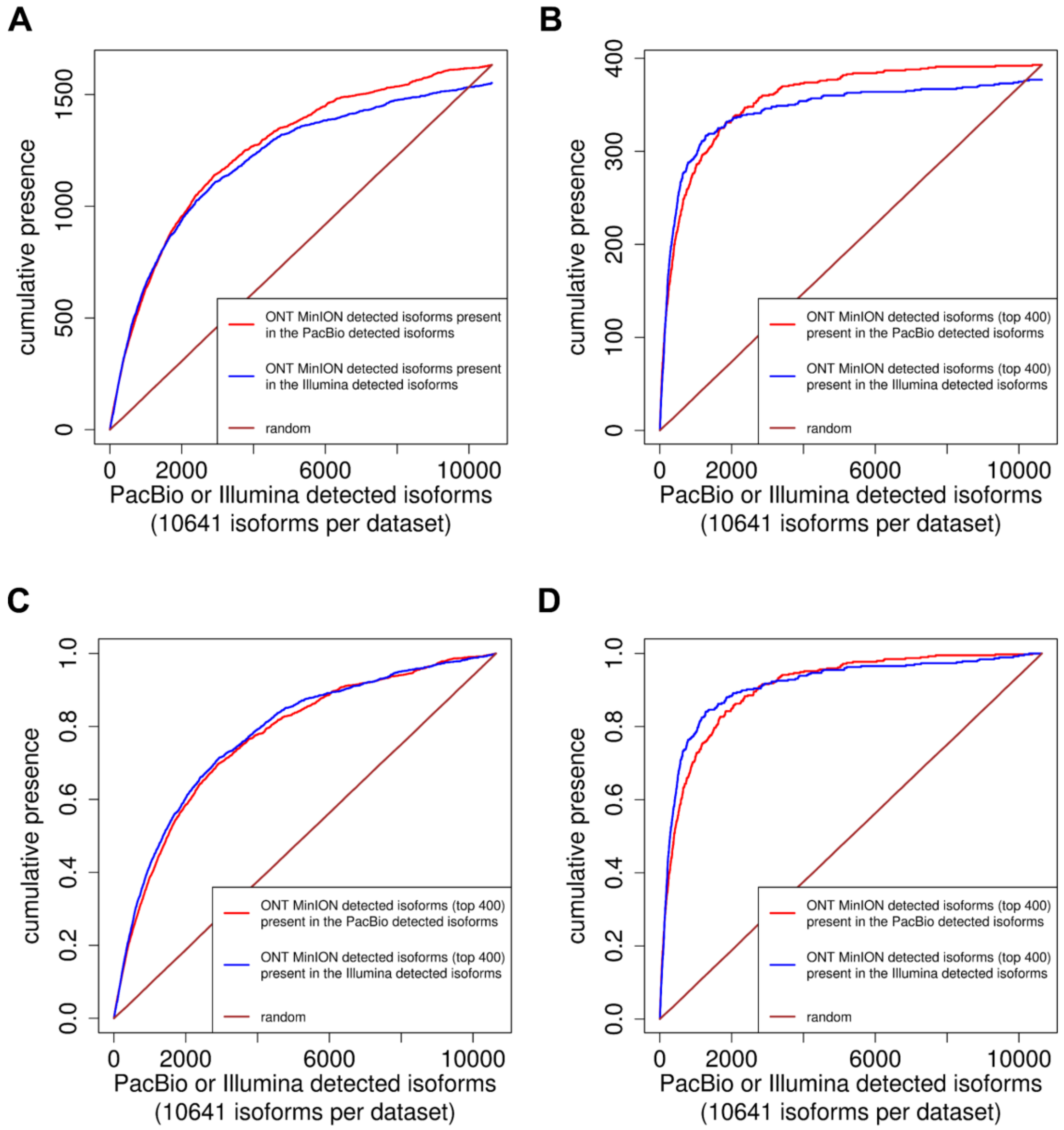

Supplementary Figure S25. Similarity between the isoforms detected on the ONT MinION, PacBio RS II and Illumina HiSeq 2500 platfoms. A) The comparison between ONT MinION and PacBio RS II (red line) as well as between ONT MinION and Illumina HiSeq 2500 are presented (blue line). For the Illumina

HiSeq 2500 we select equal number from the top expressed isoforms as the number of isoforms detected with the PacBio RS II platform (10641 isoforms in each case). In the ONT MinION platform 1846 isoforms are detected with at least 1 MinION read. For the PacBio RS II or Illumina isoform expression datasets, the reads are sorted from the most abundant (left side of x axis) to the least abundant isoforms (right side of x axis). The existence of a common isoform between the ONT MinION platform and the PacBio RS II or Illumina HiSeq 2500 platforms is indicated as cumulative presence over the rank-ordered isoform expression. B) Similar with (A) but only the top 400 expressed isoforms from the ONT MinION platform are used. The (C) and (D) graphs correspond to the (A) and (B) ones, respectively, but the maximum y axis values are scaled to 1 (proportional scaling for the rest of the y axis values). The scaling is performed in order to show better that the common MinION/Illumina isoforms are more abundant on the top expressed Illumina isoforms than the common MinION/PacBio isoforms on the top expressed PacBio isoforms. This is prominent for the top 400 expressed MinION isoforms.

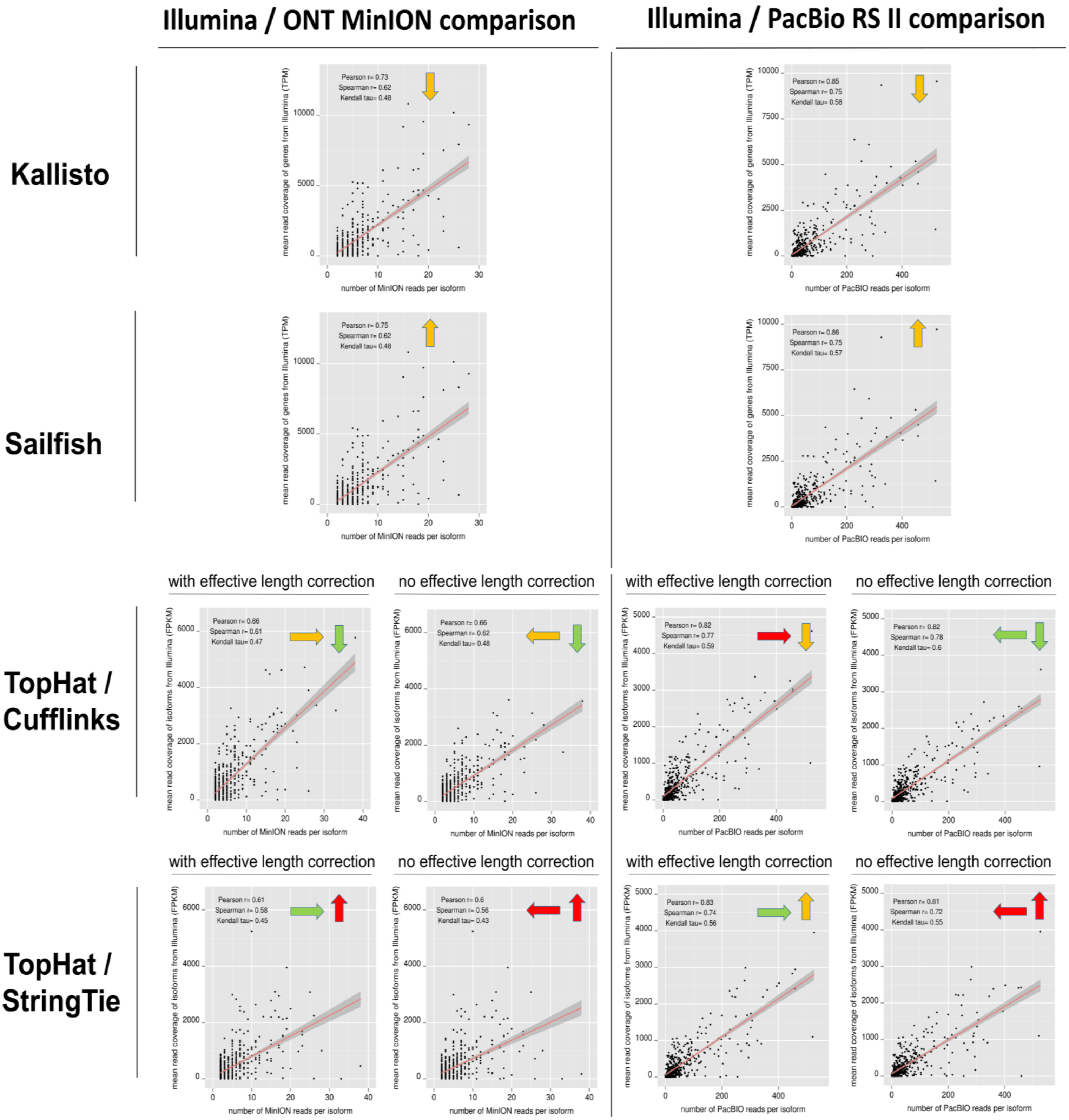
Illumina / ONT MinION comparison
Illumina / PacBio RS II comparison
Kallisto
Sailfish
TopHat / Cufflinks
TopHat / StringTie
with effective length correction
no effective length correction
Pearson r= 0.73
Spearman r= 0.62
Kendall tau= 0.48
Pearson r= 0.85
Spearman r= 0.75
Kendall tau= 0.58
Pearson r= 0.75
Spearman r= 0.62
Kendall tau= 0.48
Pearson r= 0.86
Spearman r= 0.75
Kendall tau= 0.57
Pearson r= 0.66
Spearman r= 0.61
Kendall tau= 0.47
Pearson r= 0.66
Spearman r= 0.62
Kendall tau= 0.48
Pearson r= 0.82
Spearman r= 0.77
Kendall tau= 0.59
Pearson r= 0.82
Spearman r= 0.78
Kendall tau= 0.6
Pearson r= 0.61
Spearman r= 0.58
Kendall tau= 0.45
Pearson r= 0.6
Spearman r= 0.56
Kendall tau= 0.43
Pearson r= 0.83
Spearman r= 0.74
Kendall tau= 0.56
Pearson r= 0.81
Spearman r= 0.72
Kendall tau= 0.55
mean read coverage of genes from Illumina (TPM)
mean read coverage of isoforms from Illumina (FPKM)
number of MinION reads per isoform
number of PacBIO reads per isoform

Supplementary Figure S26 (previous page). Effect of different quantification methods in the agreement between the Illumina and the ONT MinION or the PacBio RS II estimated cDNA abundance for the HEK-293 isoforms. The FPKM values from the TopHat / Cufflinks and the TopHat / StringTie pipelines are presented. Additionally, the TPM values from two kmer quantification aligners like Kallisto and Sailfish are also presented. In the case of the TopHat / Cufflinks and the TopHat / StringTie pipelines we used different quantification parameters, with and without effective length correction as indicated. The color of the horizontal arrows indicates the effect of comparison for the conditions in the presence and absence of effective length correction for a specific aligner and for a specific contrast (either the Illumina/ONT MinION or the Illumina/PacBio RS II contrast). The condition with the green color of the horizontal arrow has a better correlation than the other condition with which it is compared. The worse condition has a red color horizontal arrow. The orange color arrow indicates that both conditions are equally good. The right or left direction of the tip of the horizontal arrow points toward the position of the other condition that it is compared. The color of the vertical arrows indicates the effect of comparison between the two alignment pipelines (TopHat / Cufflinks and the TopHat / StringTie) for specific alignment parameters (presence or absence of effective length correction) and for a specific contrast (either the Illumina/ONT MinION or the Illumina/PacBio RS II contrast). The color of the vertical arrows has the same meaning as the color of the horizontal arrows

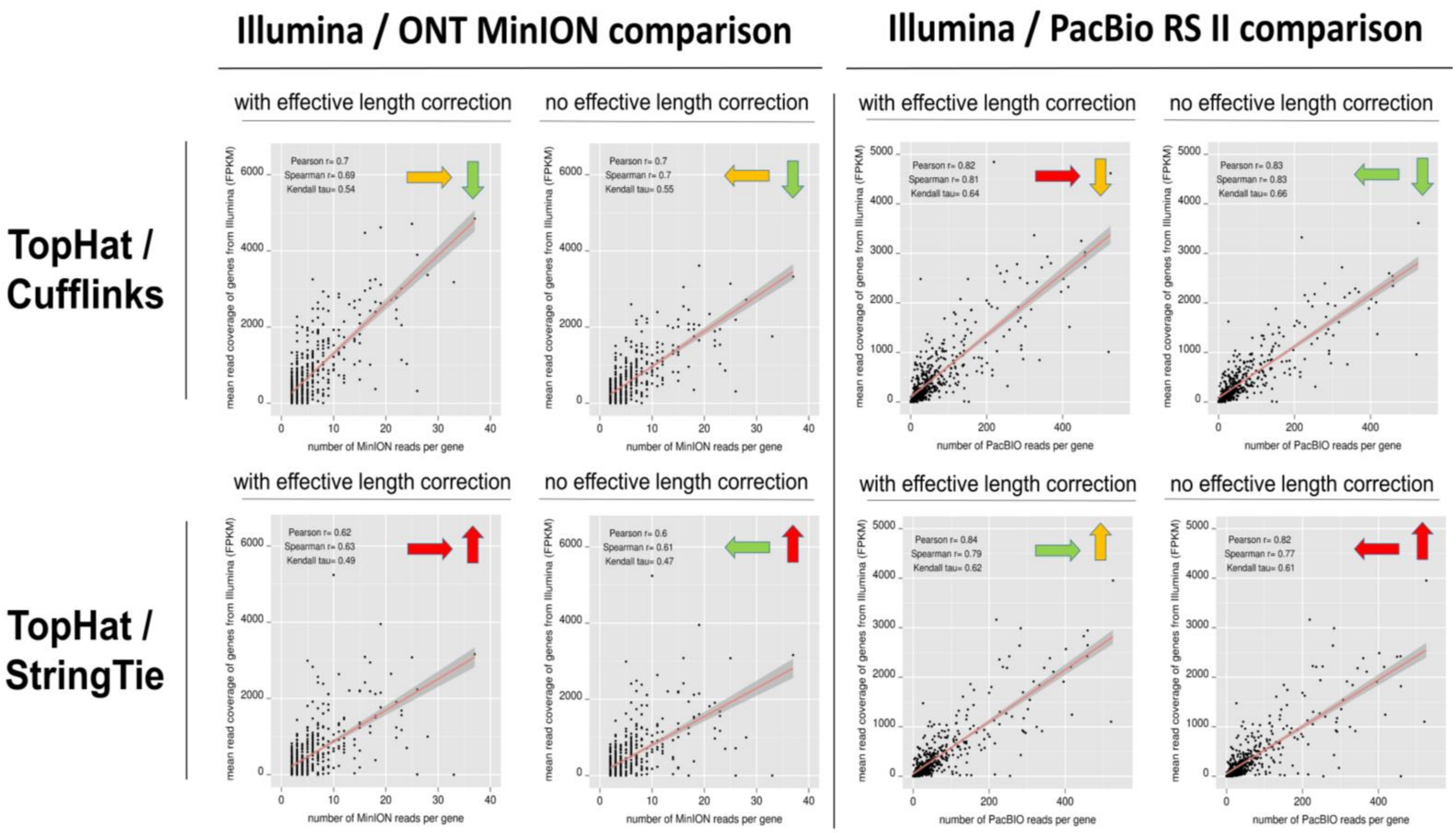

Supplementary Figure S27 . Effect of different quantification methods in the agreement between the Illumina and the ONT MinION or the PacBio RS II estimated cDNA abundance for the HEK-293 genes. The FPKM values from the TopHat / Cufflinks and the TopHat / StringTie pipelines are presented. In the case of the TopHat / Cufflinks and the TopHat / StringTie pipelines we used different quantification parameters, with and without effective length correction as indicated. The explanation of the arrows and their colors is similar with the one presented on Supplementary Fig. S26.

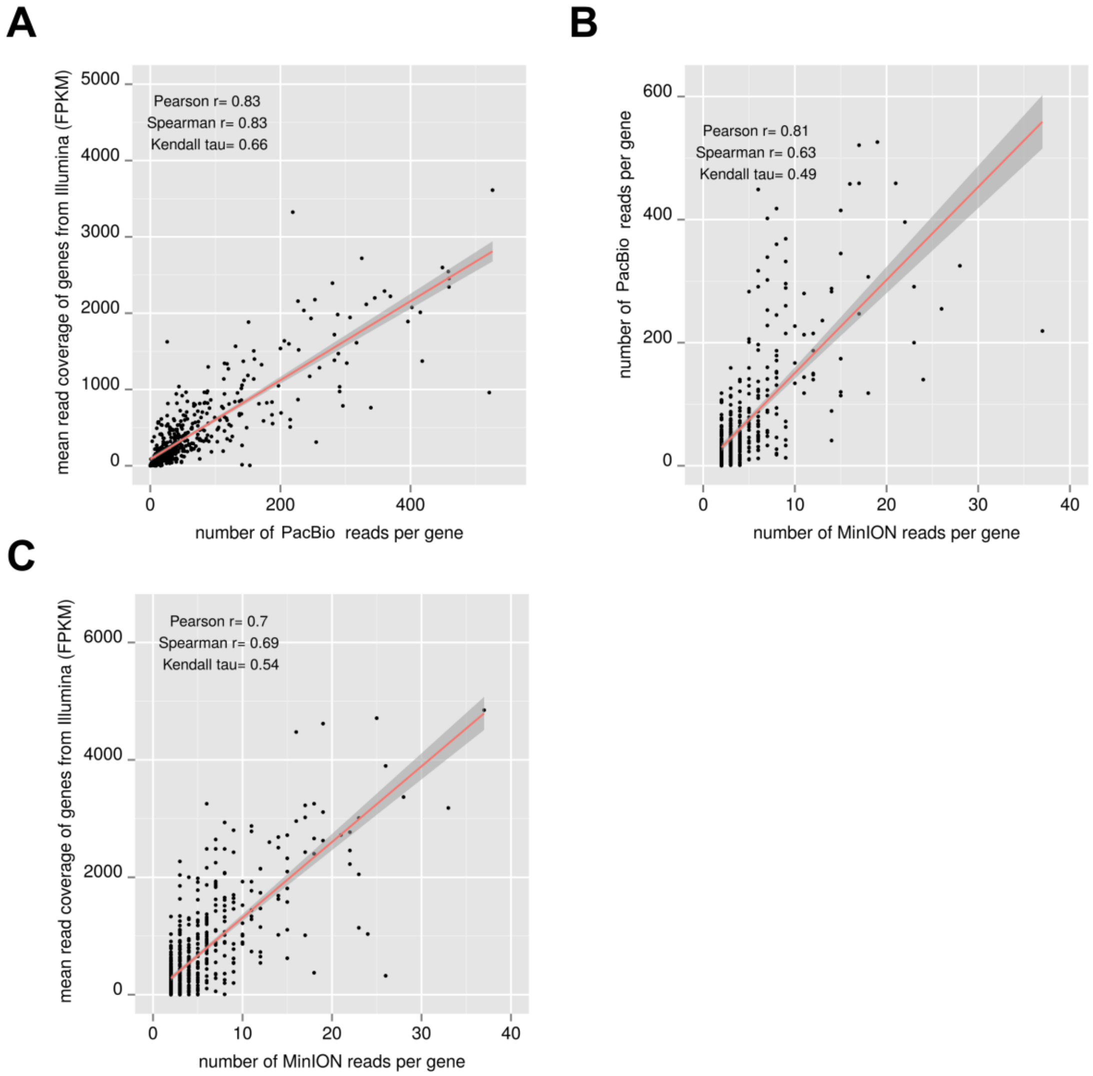

Supplementary Figure S28. Estimation of the HEK-293 cDNA gene abundance with three sequencing platforms. The comparison between the cDNA gene abundance estimated from the Illumina HiSeq 2500 platform and from the PacBio RS II platform is presented in (A). The expression level of the HEK-293 genes estimated with the ONT MinION platform is compared with the one calculated from either the PacBio RS II (B) or the Illumina HiSeq 2500 platform (C). For the Illumina HiSeq 2500 platform the expression level is calculated with the

FPKM method using the TopHat / Cufflinks pipeline without the effective length correction alignment parameter. For the PacBio RS II or the ONT MinION platform the counts of sequenced molecules per gene are presented.

.

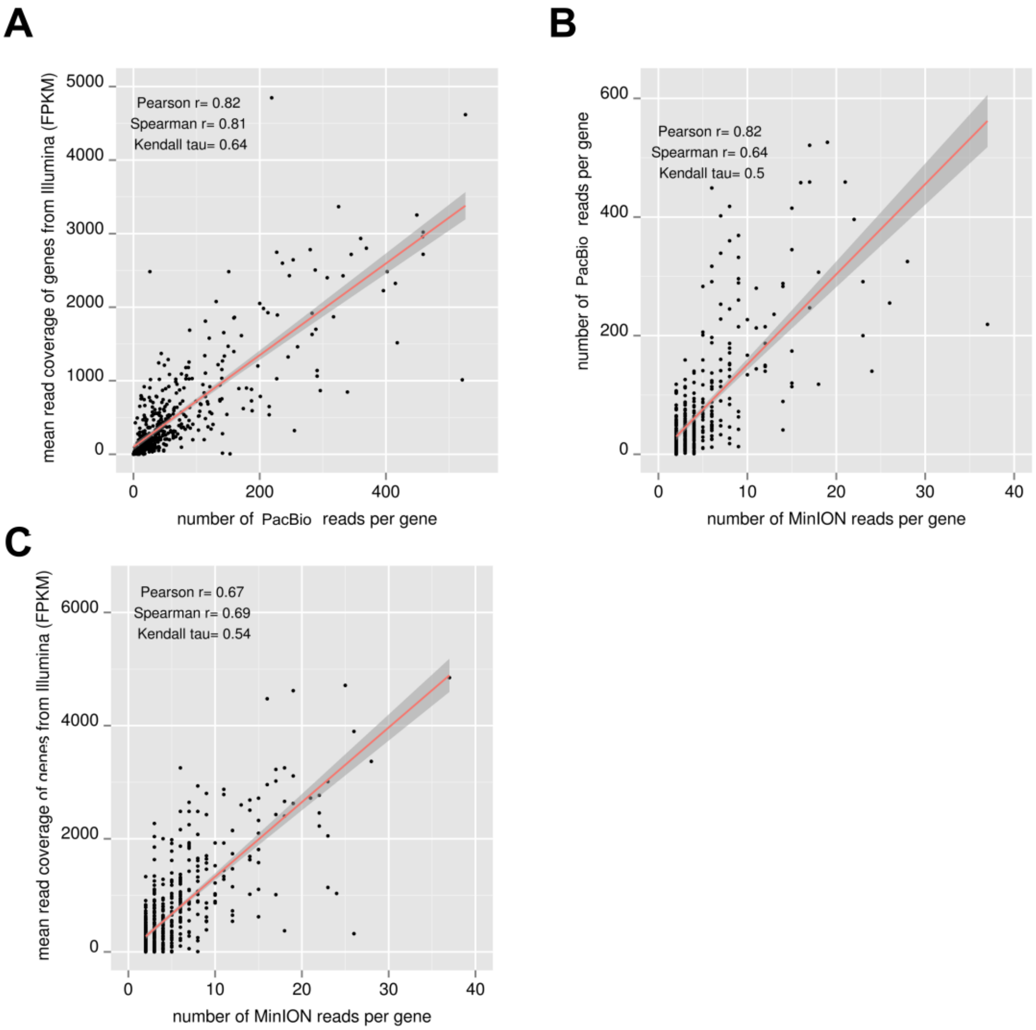

Supplementary Figure S29. Estimation of the HEK-293 cDNA gene abundance with three sequencing platforms after removing a set of problematic genes. It has been shown that the cDNA abundance of these problematic genes cannot be accurately estimated with the short-read sequenced fragments from the Illumina platform[6]. The comparison between the cDNA gene abundance estimated from the Illumina HiSeq 2500 platform and from the PacBio RS II platform is presented in (A). The expression level of the HEK-293 genes estimated with the ONT MinION platform is compared with the one calculated from

either the PacBio RS II (B) or the Illumina HiSeq 2500 platform (C). For the Illumina HiSeq 2500 platform the expression level is calculated with the FPKM method using the TopHat / Cufflinks pipeline without the effective length correction alignment parameter. For the PacBio RS II or the ONT MinION platform the counts of sequenced molecules per gene are presented.

template switch primer:
5'-AAGCAGTGGTATCAACGCAGAGT-*YYYYYYYYNN*-ACGC**rGrGrG**-3′

oligo-dT30V primer:
5'-AAGCAGTGGTATCAACGCAGAGT-*RRRRRRRRNN*-AC[T]$_{30}$VN-3′

cDNA amplification primer:
5'-**TCGTCGGCAGCGTC**-AAGCAGTGGTATCAACGCAGAGT-3′

Supplementary Figure S30. DNA sequence of the template switch primer, the oligo-dT30V primer and the cDNA amplification primer used during the ERCC cDNA synthesis. The red bases correspond to ribonucleotides.

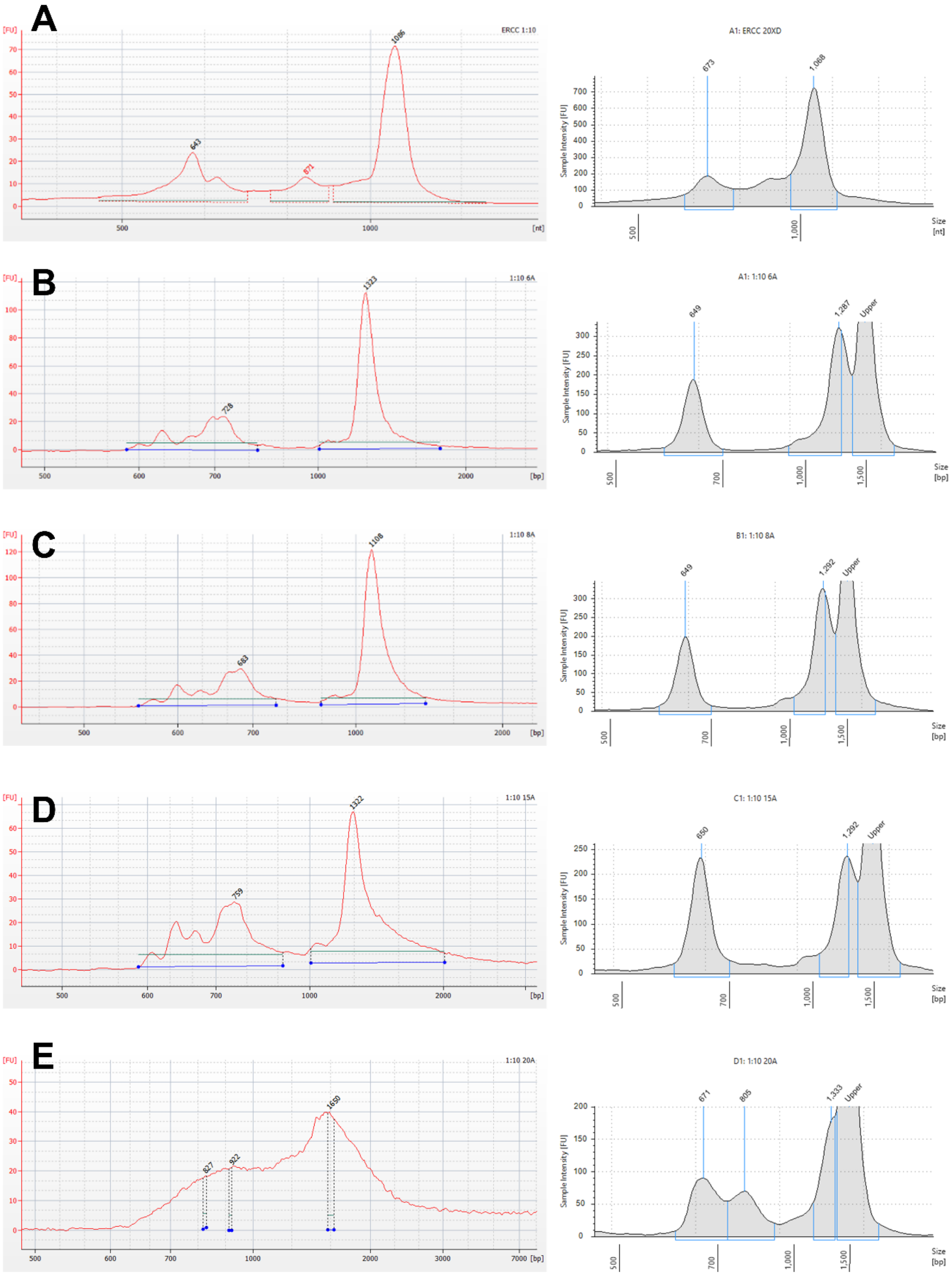
A
ERCC 1:10
A1: ERCC 20XD
B
1:10 6A
A1: 1:10 6A
C
1:10 8A
B1: 1:10 8A
D
1:10 15A
C1: 1:10 15A
E
1:10 20A
D1: 1:10 20A
Sample Intensity [FU]
Size [bp]

Supplementary Figure S31 (previous page). Electropherogram profile of the ERCC RNA (A) and cDNA (B-E) populations. The RNA and cDNA profiles were derived from either the Agilent Bioanalyser (first column) or the Agilent Tapestation (second column). For the cDNA, profiles from different PCR cycles of the cDNA amplification reaction are shown as follows: 6 PCR cycles (B), 8 PCR cycles (C), 14 PCR cycles (D), 20 PCR cycles (E). The surface ratio of the 900bp-1300bp group to the 550bp-800bp group is for the different cDNA amplification cycles and instruments: 6 PCR cycles ($ratio_{bioanalyser}$=2.1 , $ratio_{tapestation}$=2.6), 8 PCR cycles ($ratio_{bioanalyser}$=1.9 , $ratio_{tapestation}$=2.4), 14 PCR cycles ($ratio_{bioanalyser}$=1.3 , $ratio_{tapestation}$=1.4). The surface ratio of the 900bp-1300bp group to the 550bp-800bp group for the RNA profile is $ratio_{bioanalyser}$=2.16 , $ratio_{tapestation}$ =2.8. The starting amount of ERCC RNA in the cDNA synthesis reaction is 3.2 ngs.

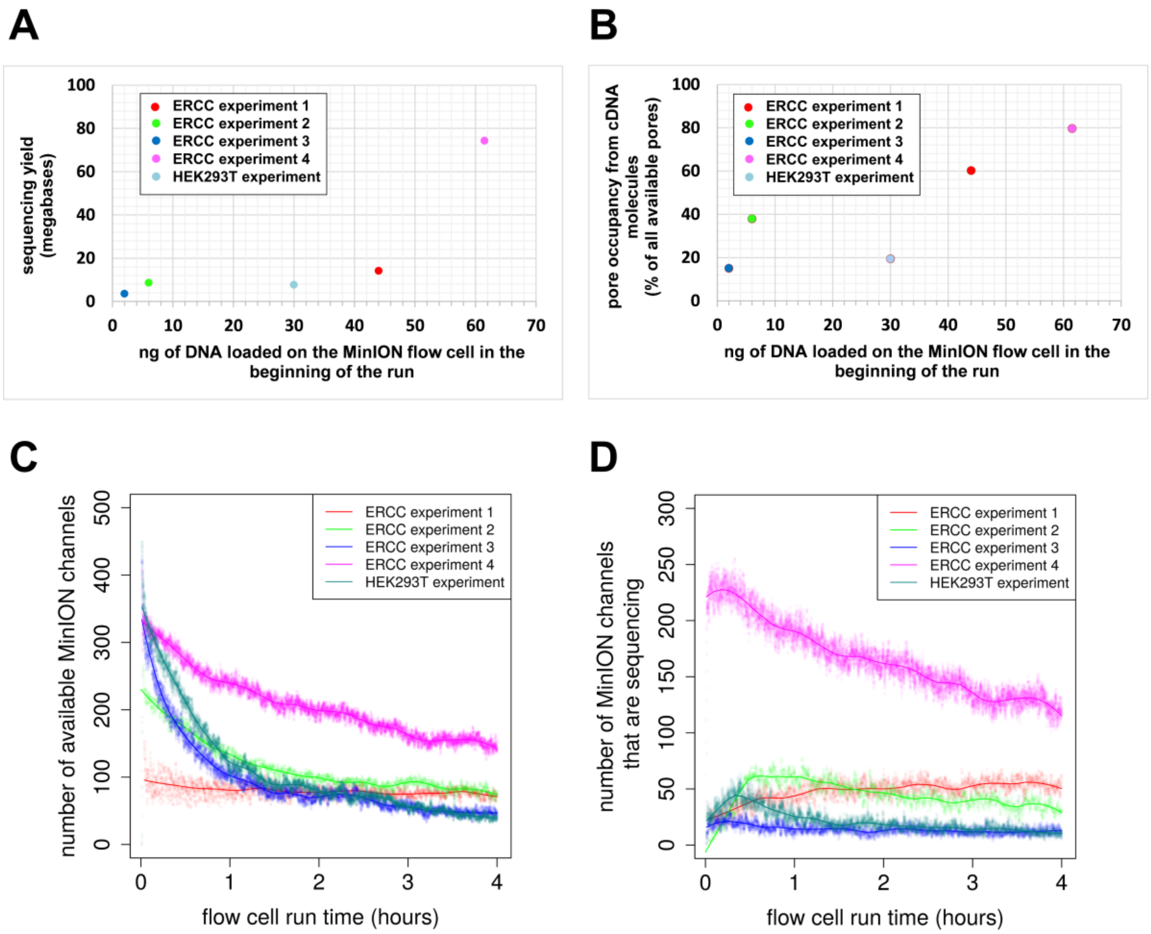

Supplementary Figure S32. Performance of the five ONT MinION flow cells used in this study. (A) Number of bases produced in the first 4 hours of the sequencing run in relationship to the amount of the DNA loaded on the flow cell. (B) Pore occupancy during the first 4 hours of the sequencing run in relationship to the amount of the DNA loaded on the flow cell. The pore occupancy for each MinION flowcell is expressed as the ratio of the area under curve in figure (D) to the area under curve in (C). (C) Number of available sequencing pores at each

time point during the first 4 hours of sequencing run. (D) Number of sequencing pores at each time point during the first 4 hours of sequencing run. Only the first 4 hours of the sequencing run for all the MinION flow cells are shown as representative of an uninterrupted sequencing run performance because in some ERCC experiments, but not all, new cDNA library was introduced in the flow cell at the end of this time period. As seen in (C) the high amount of cDNA loaded in the flow cell, which corresponds to an increased number of pores sequencing (D), might delay the reduction in the number of available channels/pores over time.

Supplementary Tables

A

ERCC experiment	null hypothesis	alternative hypothesis	Spearman 2 tail p-value	Pearson 2 tail p-value	Kendall 2 tail p-value	Type of difference
1	complement reads are similar to the template reads	complement reads are different from the template reads	0.13	0	0,22	magnitude
	2D reads are similar to the template reads	2D reads are different from the template reads	0.11	0	0.12	magnitude
2	high quality template reads are similar to the low quality template reads	high quality template reads are different from the low quality template reads	0.02	0	0	magnitude and rank
	low quality complement reads are similar to the low quality template reads	low quality complement reads are different from the low quality template reads	0.02	0	0.03	magnitude and rank
	low quality 2D reads are similar to the low quality template reads	low quality 2D reads are different from the low quality template reads	0	0	0.02	magnitude and rank
3	high quality template reads are similar to the low quality template reads	high quality template reads are different from the low quality template reads	0.69	0.22	0.87	
	low quality complement reads are similar to the low quality template reads	low quality complement reads are different from the low quality template reads	0.04	0	0.02	magnitude and rank
	low quality 2D reads are similar to the low quality template reads	low quality 2D reads are different from the low quality template reads	0.1	0.5	0.13	
4	high quality template reads are similar to the low quality template reads	high quality template reads are different from the low quality template reads	0.95	0	0.72	magnitude
	low quality complement reads are similar to the low quality template reads	low quality complement reads are different from the low quality template reads	0.81	0	0.74	magnitude
	low quality 2D reads are similar to the low quality template reads	low quality 2D reads are different from the low quality template reads	0.01	0	0.12	magnitude and rank

B

ERCC experiment	null hypothesis	alternative hypothesis	Spearman 1 tail p-value	Pearson 1 tail p-value	Kendall 1 tail p-value
1	template reads closer to Illumina molecular counts	complement reads closer to Illumina molecular counts	0.94	1	0.89
	template reads closer to Illumina molecular counts	2D reads closer to Illumina molecular counts	0.95	1	0.94
2	low quality template reads closer to Illumina molecular counts	high quality template reads closer to Illumina molecular counts	0.99	1	1
	low quality template reads closer to Illumina molecular counts	low quality complement reads closer to Illumina molecular counts	0.99	1	0.99
	low quality template reads closer to Illumina molecular counts	low quality 2D reads closer to Illumina molecular counts	0	1	0.01
3	low quality template reads closer to Illumina molecular counts	low quality complement reads closer to Illumina molecular counts	0.98	1	0.99
4	low quality template reads closer to Illumina molecular counts	high quality template reads closer to Illumina molecular counts	0.48	1	0.62
	low quality template reads closer to Illumina molecular counts	low quality complement reads closer to Illumina molecular counts	0.41	1	0.63
	low quality template reads closer to Illumina molecular counts	low quality 2D reads closer to Illumina molecular counts	0	1	0.06

Supplementary Table S1 (previous page). Summary results of the comparisons presented in Supplementary Fig. S9 and Supplementary Fig. S10. The red colored boxes indicate conditions where the p-value is less than 0.05. The blue colored boxes indicate the hypotheses accepted after the hypothesis testing. Table (A): initial hypotheses and 2-tail p-values; table (B) further analysis of cases where at least one of the corresponding 2-tail p-values, presented in table (A), is less than 0.05 . In table (A) we reject the null hypothesis if at least one of the 2-tail p-values, from either the Pearson/Spearman/Kendall correlation metrics, is less than 0.05. In table (B) we reject the null hypothesis if one of the following happens: i) Only the 1-tail p-value of the Pearson correlation metric is less than 0.05, ii) All the 1-tail p-values of the Pearson/Spearman/Kendall correlation metrics are less than 0.05. In table (A) if the ERCC cDNA abundance as estimated from either the high quality template reads, the low quality complement reads or the low quality 2D reads, differs from the one estimated from the low quality template reads, then we report that the compared cDNA abundance values of the examined read groups differ in their “magnitude” if the 2-tail p-value of the Pearson correlation metric is < 0.05 or that they differ in their “rank” if the 2-tail p-value of the Spearman or Kendall correlation metric is < 0.05. In table (B) the green colored boxes indicate that the 1-tail p-value of the Pearson correlation metric is prioritized over the 1-tail p-value from the Spearman or Kendall correlation metrics. A detailed explanation of the figure and the selection criteria used is presented in Supplementary methods section VIII.

**aliases used in Supplementary Fig. S11 - Supplementary Fig. S13**	**aligner version**	**alignment parameters**	**references**
LAST_1	LAST v475 [7]	lastal -a1 -b1 –q1 -s 2 -T 0 -Q 0 –e 45	[8]
LAST_2	LAST v475 [7]	lastal -a1 -b1 -q2 -s 2 -T 0 -Q 0 –e 40	[9]
LAST_3	LAST v475 [7]	lastal -a1 -b1 –q1 -s 2 -T 0 -Q 0 –e 40	[10-13]
LAST_4	LAST v475 [7]	lastal -a1 -b1 –q1 -s 2 -T 0 -Q 0 –e 30	
BLAST_1	BLASTn 2.2.28+[14]	default parameters	[15]
BLAST_2	BLASTn 2.2.28+[14]	word size=11, reward=2, penalty=−3, gapopen=2 and gapextend=2	[10]
BWAmem_1	BWAmem v 0.7.12-r1039[16]	-x pacbio	[11]
BWAmem_2	BWAmem v 0.7.12-r1039[16]	-x ONT	
Sm_Waterman	SMITH-WATERMAN[17]	match score= +5, mismatch penalty= -4, gap/extend penalty= -8 (top 3000 reads with the highest score were selected)	[18]
BLASr_1	BLASr 2.0.0 [19]	gap open penalty = 10, gap extension penalty = 0, minimum seed length = 12	[3]
BLASr_2	BLASr 2.0.0 [19]	gap open penalty = 0, gap extension penalty = 0, minimum seed length = 12	[3,12]
MarginAlign_1	MarginAlign[20]	default options: (LAST aligner)	[20]
MarginAlign_2	MarginAlign[20]	option: -bwa	[20]

Supplementary Table S2 (previous page). Alignment parameters for the indicated aligners as are retrieved from the literature.

experiment type:	ERCC experiment 1	ERCC experiment 2	ERCC experiment 3	ERCC experiment 4	HEK-293
flowcell version	r7	r7.3	r7.3	r7.3	r7.3
library prep version	SQK-MAP003	SQK-MAP004	SQK-MAP005	SQK-MAP005	SQK-MAP005
starting amount of cDNA	0.45 ug	1 ug	2 ug	3 ug	0.5 ug
amount of cDNA in the "end repair reaction"	0.45 ug (1 reaction)	1 ug (1 reaction)	2 ug (1 reaction)	1.5 ug (2 reactions)	0.5 ug (half reaction)
amount of cDNA after the "end repair reaction"	0.4 ug	0.75 ug in total	1.2 ug in total	2 ug in total	0.435 ug
amount of cDNA in the "dA tailing reaction"	0.4 ug (1 reaction)	0.75 ug (1 reaction)	1.2 ug (1 reaction)	1 ug (2 reactions)	0.43 ug (half reaction)
amount of cDNA after the "dA tailing reaction"	0.35 ug	not applicable	1 ug in total	1.76 ug in total	0.281 ug in total
amount of cDNA in the "adaptor ligation reaction"	0.35 ug (1 reaction)	not applicable	0.133 ug (1 reaction)	1.33 ug (1 reaction)	0.281 ug (1/4 reaction)
ratio of : (moles of adaptors) /(moles of cDNA)	1:1	1.6:1	10:1	1:1	1:1
amount of cDNA recovered	350 ng (with no bead enrichment step, so by extrapolation from "ERCC experiment 4" , this corresponds to 44 ng of cDNA with adaptors)	52.5 ngs in 25 ul buffer (after bead enrichment step)	17.5 ngs in 25 ul buffer (after bead enrichment step)	258 ngs in 25 ul buffer (after bead enrichment step)	30 ngs in 6 ul buffer (after bead enrichment step)

Supplementary Table S3. Amount of cDNA available at each step of the ONT library preparation procedure from the different MinION experiments.

	long transcripts quantification	short transcripts quantification	small RNA quantification	variant calling (SNP & RNA editing events)	defined TSS and TES of transcripts	isoform abundance estimation
Illumina	accurate	accurate	accurate	yes (high basecalling accuarcy)	the TSS and TES are not accurately defined with standard RNA-seq protocols (for example the TruSeq protocol)	medium accuracy, isoform abundance is statisticaly inferred
PacBio RS II	accurate	non accurate	uknown	yes, despite a 0.001%-15% read error rate. For the Consensus Circular Reads low/medium coverage is needed . For the non Consensus Circular Reads , high coverage is needed to cancel out the non-systematic errors.	full length sequencing of cDNA molecules	accurate isoform abundance estimation for the long isoforms of genes or accurate isoform abundance estimation for the short isoforms of genes
ONT MinION	accurate	accurate	uknown	possible but the per read error rate is high (7%-40%). High coverage for the high quality 2D reads and even higher for the low quality reads is needed to cancel out the non-systematic errors	full length sequencing of cDNA molecules	accurate isoform abundance estimation for both the long and the short isoforms of genes

Supplementary Table S4 (previous page). Cases where the different platforms can be used either exclusively or interchangeably.

A

	ERCC 14 PCR cycles of cDNA amplification		ERCC 21 PCR cycles of cDNA amplification
**all fragments**	**1098753**		**338677**
**paired reads**	1094474		338385
**unpaired reads**	4279		292
**total mapped fragments**	**1077880**		**334336**
**mapped paired reads**			
pairs where both the R1 and the R2 reads align	817236		266882
pairs where only the R1 reads align	117387		35509
pairs where only the R2 reads align	139627		31681
**mapped unpaired reads**	3630		264

B

	ERCC 14 PCR cycles of cDNA amplification		ERCC 21 PCR cycles of cDNA amplification
number of pairs with the 5' adaptor	39125		5878
number of pairs with the 5' adaptor where both the R1 and the R2 reads align	38428		5808
number of pairs with the 5' adaptor where either the R1 or the R2 reads align	339		37
number of pairs with the 5' adaptor where the R1 read was aligned but the R2 read was not used during the alignment process	38592		5835
number of pairs with the 5' adaptor where the R2 read was aligned but the R1 read was not used during the alignment process	38348		5811
number of pairs with the 3' adaptor	14393		1242
number of pairs with the 3' adaptor where both the R1 and the R2 reads align	2233		416
number of pairs with the 3' adaptor where either the R1 or the R2 reads align	4817		31
number of pairs with the 3' adaptor where the R1 read was aligned but the R2 read was not used during the alignment process	191		374
number of pairs with the 3' adaptor where the R2 read was aligned but the R1 read was not used during the alignment process	13050		705

C

	ERCC 14 PCR cycles of cDNA amplification		ERCC 21 PCR cycles of cDNA amplification
number of pairs with the 5' adaptor that showed the same UMI two or more times	806		27
expected number of pairs with the 5' adaptor that showed by chance the same UMI two or more times	378		13
number of pairs with the 3' adaptor that showed the same UMI two or more times	133		1
expected number of pairs with the 5' adaptor that showed by chance the same UMI two or more times	58		0

Supplementary Table S5. Number of ERCC cDNA fragments sequenced on the Illumina platforms. The ERCC experiments with the 14 and 21 PCR cycles of cDNA amplification are presented. A) Number of Illumina fragments sequenced and aligned on the reference database. B) Number of sequenced (blue color

boxes) and aligned (red color boxes) Illumina fragments that correspond to the 5' or the 3' end of the ERCC cDNA molecules. C) Number of 5' or 3' end ERCC cDNA fragments that show the same UMI 2 or more times (yellow color boxes). The expected number of UMI by chance is also presented (green color boxes).

experiment type:<br>flowcell version<br>library prep version	ERCC experiment 1<br>r7<br>SQK-MAP003		ERCC experiment 2<br>r7.3<br>SQK-MAP004		ERCC experiment 3<br>r7.3<br>SQK-MAP005	
	loading time (h):	amount of cDNA loaded:	loading time (h):	amount of cDNA loaded:	loading time (h):	amount of cDNA loaded:
	0	350 ng	0	6 ng (3ul)	0.0	2 ngs (3ul)
			4	6 ng (3ul)	11.9	6 ngs (9ul)
			8	6 ng (3ul)	31.5	9.5 ngs (13.6ul)
			11	6 ng (3ul)	35.8	6 ngs (from another ERCC library preparation)
			20.3	12 ng (6ul)		
			24.3	6 ng (3ul)		
			31.6	3 ng (1.5 ul)		
	restart time (h):		restart time (h):		restart time (h):	
	0.00		0.0		0.0	
			44.6		11.9	
			72.7		16.9	
					31.5	
					33.0	
					35.8	
					48.5	
					52.4	
					54.3	
					61.0	
					62.8	

experiment type:<br>flowcell version<br>library prep version	ERCC experiment 4<br>r7.3<br>SQK-MAP005		HEK-293<br>r7.3<br>SQK-MAP005	
	loading time (h):	amount of cDNA loaded:	loading time (h):	amount of cDNA loaded:
	0	61.5 ngs (6 ul)	0	30ng (6ul)
	23.7	61.5 ngs (6 ul)		
	restart time (h):		restart time (h):	
	0.0		0.0	
	10.7		5.5	
	23.7		16.5	
	31.7			
	32.7			
	42.5			
	43.3			
	64.4			
	67.3			

Supplementary Table S6. Amount of cDNA loaded on the flow cell during the different MinION experiments. The time points when the DNA was loaded are indicated. The time points when the machine was restarted during the sequencing run is also indicated.

**HEK-293 cDNA PacBio RS II reads**	
number of reads in the CCS group	9685
number of aligned reads from the CCS group	8978
number of reads in the "longest subread" group	104550
number of aligned reads from the "longest subread" group	80552
**ERCC cDNA PacBio RS II reads**	
number of reads in the "longest subread" group	159328
number of aligned reads from the "longest subread" group	133849

Supplementary Table S7. Number of sequenced and aligned PacBio RS II reads from the HEK-293 cDNA and the ERCC cDNA. Depending on the dataset the reads of only the "longest subread" group or both the "longest subread" group and the "Circular Consensus Sequencing" (CCS) group are presented.

Supplementary text

I. Variability in the ONT MinION flowcell performance

The proportion of sequenced reads assigned to the complement read group, the 2D read group and the high quality category from this study in comparison to other studies is presented in Supplementary Fig. S2. Similar to the data from Ip et al[1] we see a variability in the percentage of the complement read group, the 2D read group and the high quality category relative to the template read group for the different runs. We also observe that the fraction of reads in the complement and the 2D read groups is lower than in the other presented studies.

For the ERCC experiments number 2, 3, 4 we used the same low complexity ERCC cDNA aliquot from the 14 cycles of cDNA amplification. Additionally, minor modifications on the standard library preparation protocol were performed in order to increase the yield of the adaptor ligated cDNA that is loaded on the MinION flowcell (Supplementary Table S3). The quality of the adaptor ligated cDNA seems similar in all these three runs (see Supplementary text section III). Consequently, the observed differences can be attributed to the performance of the ONT MinION flowcell run. In the case of the HEK-293 cDNA library the high complexity of the cDNA library can also affect negatively the observed fraction of the complement and the 2D reads.

II. Estimation of ERCC transcript abundance with the Illumina HiSeq 2500 or MiSeq platforms

Full length cDNAs tagged with unique molecular identifiers (UMIs) on both the 5' and 3' ends of the cDNA were produced from the ERCC 92 spike RNAs standards. These full length cDNAs also featured Illumina compatible P5 motifs on both 5' and 3' ends to increase the rescue of the ends after tagmentation with the Illumina Nextera XT method. Following alignment, the ERCC cDNA abundance was estimated using the FPKM[21] or TPM[22] methods (Supplementary Fig. S4A, B) as well as molecular counting using the 5' end and 3' end fragments (Supplementary Fig. S4C-F).

We compared how close is the observed ERCC cDNA abundance to the ERCC RNA abundance, as provided from the manufacturer (Ambion). The expectation is that if there is no significant bias in either the cDNA synthesis (PCR bias during cDNA amplification), the sequencing method (PCR amplification bias after tagmentation, PCR amplification bias during the cluster generation on the Illumina platform) or the gene expression estimation methods (FPKM method or molecular counting) the two values must be close together. In our design as we did not have enough UMI (4096 available UMI) to barcode the original RNA molecules and the ONT MinION cannot accurately basecall them, we cannot address the cDNA

amplification PCR bias by estimating the original RNA abundance. Comparison between the Illumina estimated ERCC cDNA abundance and the ONT MinION ERCC cDNA abundance can reveal biases introduced during the tagmentation PCR amplification and the Illumina cluster generation PCR amplification (main text). Here we compared the accuracy in the cDNA abundance estimation between the FPKM and the molecular counting approach by examining how close they are to the expected ERCC RNA abundance.

Large deviations from the expected ERCC RNA abundance were observed when using values derived from the FPKM method ($r_{pearson\ 21\ PCR\ cycles}$ =0.46 and $r_{pearson\ 14\ PCR\ cycles}$ =0.72 for 21 and 14 PCR cycles of cDNA amplification respectively), and to a lesser extent when inferring using molecular counting from either the 5' end of the cDNA molecules ($r_{pearson\ 21\ PCR\ cycles}$ =0.81, $r_{pearson\ 14\ PCR\ cycles}$ =0.89) or the 3' end of the cDNA molecules ($r_{pearson\ 21\ PCR\ cycles}$ =0.74, $r_{pearson\ 14\ PCR\ cycles}$ =0.88). We also noticed that the ERCC expression level estimation with the FPKM method, relative to the Ambion RNA counts (Supplementary Fig. S19A, B) or to the 5' end fragment molecular counting (Supplementary Fig. S19E, F), is biased towards underrepresenting long ERCC transcripts or ERCC transcripts with high GC content. This indicates that the molecular counts is a better estimator of cDNA transcript abundance than the FPKM method (or its equivalent TPM method). As we had aligned more Illumina reads from the 5' end of the molecules rather than the 3' end (Supplementary Table S5), we used the 5' end reads as representative of the number of molecules sequenced with the Illumina platform.

Twenty two percent of the variation (r^2=0.217) between the expected ERCC RNA abundance and the observed ERCC cDNA molecular counts from the Illumina platform can be attributed to the length of the transcripts (Supplementary Fig. S19D). In Supplementary Fig. S19D we see that the short ERCC molecules (length < 800bp) showed a higher number of Illumina 5' end cDNA fragments than expected from the original ERCC RNA concentration from Ambion. Similarly, when we examined the electrophoresis profile of the ERCC cDNA library on both an Agilent Bioanalyzer and an Agilent TapeStation instrument, we observed that the ERCC transcripts with length between 400-700 bp were more abundant (either one type or multiple types of ERCC transcripts) relative to the 900-1200 bp group at the cDNA level (Supplementary Fig. S31C,D) rather than the RNA level (Supplementary Fig. S31A). This indicates that the cDNA amplification reaction preferentially amplified short cDNA molecules at the expense of longer ones. Increased rounds of PCR cycles during the cDNA amplification make the deviation from the expected RNA distribution more prominent (Supplementary Fig. S31). Consequently, the Illumina estimated cDNA abundance is closer to the true ERCC cDNA library abundance at the end of the cDNA amplification procedure.

III. Assessment of the quality of the ERCC cDNA library loaded on the MinION flowcells

To assess whether the produced cDNA molecules were fragmented during the ONT MinION library preparation procedure we examined the size distribution of the sequenced fragments from the 2D reads group for the ERCC runs 1, 2, 3, 4 (Supplementary Fig. S17). The expected size distribution of the ERCC cDNA library as calculated from the Caliper Labchip GX instrument is also presented (Supplementary Fig. S17). The ERCC experiments number 2, 3, 4 were produced from the same ERCC cDNA aliquot derived from the 14 cycles of cDNA amplification. The ERCC experiment number 1 was produced from the ERCC cDNA library derived from the 21 cycles of cDNA amplification. In Supplementary Fig. S17 we see that the distributions of the low and high quality 2D reads show two clear distinct peaks independently of the ERCC experiment they are coming from. As the 2D reads correspond to full length sequenced molecules they are representative of the size distribution of the cDNA library which was loaded on the flow cell. The peaks on the size distribution plots for the high quality 2D reads, whenever available, are more distinct than the ones from the low quality 2D reads. This is due to the fact that the corresponding high quality template and complement sequences from each one of the high quality 2D reads are usually of the same length which is one of the criteria to categorize a 2D read as high quality. The corresponding low quality template and complement reads from each one of the low quality 2D reads can be more variable in size. In this case the derived low

quality 2D read sequence consensus will deviate more from the expected length on the ERCC reference transcript database.

All the above show that the ONT MinION library preparation procedure did not create partially fragmented molecules. On the contrary the distribution of the pseudo-read length of the template reads for the ERCC experiments number 2, 3 show two distinct peaks whereas for the ERCC experiment number 4 the peak at around 1100 bp is not clearly separated from the peak at around 700 bp as the intermediate region is heavily populated with fragments. As has already been discussed this is due to the fact that, for the ERCC experiment number 4, the template strand of some ERCC molecules was not sequenced as full length (Supplementary Fig. S15, Supplementary Fig. S16). Consequently, these sequenced reads are shorter than the expected reference transcript length and in Supplementary Fig. S17A they appear before the peak of the short ERCC transcript group as well as between the peaks of the long and short ERCC transcript groups.

IV. Effect of the ERCC transcript length and GC content in the ERCC cDNA abundance estimation with the PacBio RS II platform

It has been already discussed that the PacBio ZMW loading procedure that was used to sequence the ERCC cDNA transcripts, enriches for molecules longer than

700 bp. This is reflected as a considerable length dependent bias when the PacBio RS II estimated cDNA abundance for the ERCC transcripts, is compared against the expected number of RNA molecules as provided from the manufacturer (Ambion) (Supplementary Fig. S5C). As expected, this bias is absent when we only examine the ERCC transcripts more than 700 bp in length (Supplementary Fig. S5D). Similarly the length depended bias is absent when the PacBio RS II estimated cDNA abundance, of ERCC transcripts more than 700 bp in length, is compared either against the Illumina molecular counts (Supplementary Fig. S18A), or the ONT MinION molecular counts (Supplementary Fig. S18B).

We then compared the effect of the GC content on the deviation (log fold difference) between the observed ERCC cDNA abundance from the PacBio RS II platform and the expected ERCC RNA/cDNA abundance, from either the Ambion molecular counts (RNA concentration), the Illumina 5' end molecular counts and the ONT MinION platform. In Supplementary Fig. S18C we see that the high GC content molecules are overrepresented relative to the expected Ambion RNA concentration. Similarly, the high GC content molecules are also overrepresented when the PacBio RS II estimated cDNA abundance is compared against the ONT MinION derived cDNA abundance (Supplementary Fig. S18D). This is expected as the ONT MinION platform shows no length and GC bias relative to the expected Ambion RNA concentration (Fig. 2A, C). The comparison of the PacBio RS II estimated cDNA abundance with the one derived from the Illumina molecular counts showed an underrepresentation of the low GC content ERCC transcripts in

the case of the Illumina platform (Supplementary Fig. S18E). The same underrepresentation was observed when the cDNA abundance from the Illumina molecular counts was compared against the ONT MinION estimated cDNA abundance (Fig. 2B).

V. Limit of detection of the ERCC transcripts for the variable sequencing depth of the ONT MinION experiments

We used the different ERCC experiments to identify the limit of detection for the variable sequencing depths. In Supplementary Fig. S22A we present the regression lines from the different ERCC experiments that can be used to estimate the ERCC transcript frequency. The sequencing depth, the performance of the flow cells or the library preparation do not seem to have a considerable effect on the estimated cDNA abundance of the different ERCC transcripts because the regression coefficient from the different ERCC experiments is ~1. The sequencing depth affects only the detection of the lower abundance transcripts. In Supplementary Fig. S22B we present the minimum abundance frequency of a given ERCC transcript that can be detected with 2 ONT MinION reads at the different sequencing depths. The 113402, 18307, 5966, 2842 aligned ONT MinION reads can detect, with around 2 ONT MinION reads or more, transcripts with approximate abundance of at least 1 in 51164, 8125, 6257, 1170 molecules respectively.

In Supplementary Fig. S22A the different slope of the regression line from the ERCC experiment number 1 (purple color line) relative to the slope of the regression lines from the ERCC experiments number 4, 2, 3 (red, green, cyan color lines respectively) is due to the different number of cDNA amplification cycles (21 cycles for the ERCC experiment number 1 and 14 cycles for the ERCC experiments number 4, 2, 3). The increased number of cDNA amplification cycles overamplifies the lower abundant transcripts at the expense of the highly abundant ones.

VI. The Illumina short read cDNA abundance estimation is not considerably altered with different short read alignment methods and gene/isoform expression estimation methods.

Programs that use RNA-seq to estimate gene expression can give different results depending on the algorithmic details of how they handle multi-mapped or ambiguously assigned short reads[6]. Additionally, inferring the cDNA abundance of different isoforms, from short-read data, for the same gene can vary depending on the underlying assumptions of the statistical models used by different programs[23]. In the case of the HEK-293 cDNA we examined how different algorithms can affect the concordance in the estimated cDNA abundance between the Illumina RNA-seq and the ONT MinION or the PacBio RS II platform. We examined both the

concordance at the isoform level (Supplementary Fig. S26) and at the gene level (Supplementary Fig. S27). We used both the TopHat / Cufflinks[21] pipeline and the TopHat / StringTie[24] pipeline to calculate the HEK-293 cDNA gene and isoform abundance from the Illumina RNA-seq data. Similarly to Cufflinks, the StringTie software calculates the expression abundance from the TopHat aligned RNA-seq fragments. Differences in gene expression estimation should correspond to how the two algorithms treat ambiguous or multi-mapped data. Differences in isoform expression estimation should additionally correspond to the different statistical models used to assign short reads on the different isoforms of the same gene. We also used two more aligners, Kallisto[25] and Sailfish[26], which instead of aligning on the genome they create an index of unique kmers for each transcript. This choice was made because Kallisto and Sailfish has been reported to provide more accurate results[6] than Cufflinks. During the comparisons both the FPKM and the TPM expression estimation methods were used whenever available.

The -multi-read-correct parameter in Cufflinks was avoided as it causes the algorithm to perform worse[6], something that we also observed.

The default behavior of Cufflinks is to report the FPKM values based on an effective length correction. The effective length correction which instructs the software to determine the length of the transcript from the data rather than use the length in the reference database has been argued that is responsible for

overestimating the FPKM value especially for shorter genes[6]. For this reason we estimated the cDNA abundance with and without the effective length (EL) correction (-no-effective-length-correction parameter). We performed a similar approach in StringTie (–t parameter).

Initially, we examined the agreement of the correlation values for the TopHat / Cufflinks and the TopHat / StringTie pipelines for the Illumina/ONT MinION platforms comparison. We argue that one condition is different only if all the correlation values of this condition are either higher or lower than the correlation values of the other condition. In the case where some of correlation values of this condition are higher than the ones of the other condition, and the rest of the correlation values are lower or equal then we argue that the two conditions are not different. We observe that StringTie performs always worse than Cufflinks for either the isoform abundance (Supplementary Fig. S26) or the gene abundance comparisons (Supplementary Fig. S27). The presence or absence of effective length correction had no significant effect on the calculated correlation values in the case of the TopHat / Cufflinks pipeline.

For the Illumina/PacBio RS II comparison in the presence of effective length correction both the TopHat / Cufflinks and the TopHat / StringTie pipelines performed equally well, whereas in the absence of effective length correction the TopHat / Cufflinks pipeline performed better than the TopHat / StringTie pipeline

(Supplementary Fig. S26, Supplementary Fig. S27). For the TopHat / Cufflinks pipeline, the algorithm performed better in the absence than in the presence of effective length correction.

Overall the TopHat /Cufflinks pipeline performed better than the TopHat / StringTie pipeline. The absence or presence of effective length correction did not have any consistent effect on the data.

When we used the Kallisto and Sailfish aligners we observed that both performed equally well and better than the TopHat / Cufflinks pipeline, as far as it concerns the Illumina/ONT MinION comparison and the Illumina/PacBio RS II comparison (Supplementary Fig. S26). The better performance of Kallisto or Sailfish relative to Cufflinks was also observed by Roberts et al[6].

All the presented TopHat / StringTie pipeline comparisons were made with the FPKM values. We also used the reported TPM values from the StringTie output. StringTie uses the FPKM values to calculate the TPM values with a method similar to the equation presented in Supplementary methods section II. For this reason the TPM values show the exact relative abundance for the transcript isoforms as the FPKM values. Consequently the correlation values that we calculated for the TPM values, were exactly the same as the ones presented for the FPKM values and for this reason no figures are presented with the corresponding TPM values.

VII. Fragment multi-mapping as a potential confounding factor of Illumina abundance estimation

It has been reported that the Illumina platform cannot accurately quantify certain genes if the sequenced short read fragments, corresponding to a specific gene, can have multiple mapping positions[6] for example in other genes from the same family. In this case the gene expression of some of the genes in this family can be overestimated whereas for the rest of the genes is either underestimated or even absent.

To assess a potential bias in our quantification of ERCC transcripts, introduced by the multi-mapping problem described, we examined how many simulated reads from the ERCC transcripts show multi-mapping positions. For this we used a similar approach as presented by Robert et al[6] (Supplementary methods section III). We simulated fragments from the ERCC transcript database of the same length as the ones we sequenced. We performed this simulation 10 times. We then examined whether we observed multi-mapped fragments or fragments that failed to align. No multi-mapping or failed to align reads were observed in all 10 simulations. Additionally, we did not observe any fragment that aligned on a particular ERCC sequence that was different from the one it was simulated from.

This indicated that the multi-mapping problem does not affect the quantification of the ERCC transcripts.

Robert et al[6] also provided a list of 958 genes for which the expression estimation is problematic with the Illumina short reads. To examine whether these genes can significantly affect the concordance estimation between the ONT MInION and the Illumina or PacBio RS II platforms we removed these genes from the Illumina/ONT MinION, the ONT MinION/PacBio RS II and the PacBio RS II/Illumina comparisons. From the 958 problematic genes examined, 28 genes (out of 623 genes) were present in the ONT MinION gene list and had at least 2 ONT MinION reads. Additionally, 131 genes (out of 5628 genes that have an average length of isoforms more than 700 bp) were present in the PacBio RS II list and had at least 2 PacBio RS II reads. When we removed these genes from our comparisons and we recalculated the concordance in the cDNA abundance no significant difference was observed (Supplementary Fig. S29).

Supplementary methods

I. Tagmentation of the ERCC cDNA for Illumina HiSeq 2500 or MiSeq sequencing

0.375 ngs of ERCC cDNA in 1.25 ul of H_20 were tagmented using the Nextera XT DNA Library Preparation Kit (Illumina Inc). Briefly, 2.5 ul of Tagment DNA Buffer (TD) buffer were mixed with the 1.25 ul of the ERCC cDNA solution. Afterwards, 1.25 uls of Amplicon Tagment Mix (ATM) were added to the previous solution, mixed and incubated at 55°C for 5 minutes. Then, 1.25 uls of Neutralize Tagment Buffer (NT) was added to the previous solution to neutralize the tagmentase. The introduction of the Illumina P5 and P7 sequences to the end of the cDNA fragments, was done through PCR after addition of 3.75 ul of Nextera PCR Master Mix to the previous solution along with 1.25 ul of Nextera XT Index 1 Primers and 1.25 ul of Nextera XT Index 2 Primers. The thermocycling parameters were as follows: 1 cycle of [72 ^{o}C for 3 min], 1 cycle of [95 ^{o}C for 30 sec], 12 cycles of [95 ^{o}C for 10 sec, 55 ^{o}C for 30 sec, 72 ^{o}C for 1 min], 1 cycle of [72 ^{o}C for 5 min]. The samples were then cleaned with Ampure XP beads (1.9X sample volume for the ERCC sample with 14 cycles of cDNA amplification, 0.75X sample volume for the ERCC sample with 21 cycles of cDNA amplification) and processed for sequencing on the Illumina HiSeq 2500 or MiSeq platform.

II. Alignment of the ERCC cDNA fragments sequenced on the Illumina platform

cDNA fragments that were sequenced on an Illumina HiSeq 2500 or a MiSeq platform were aligned on the ERCC reference database with bowtie2[27] v2.1.0 with default parameters. The ERCC reference database was created with the sequences provided from the manufacturer (Ambion) with the exception that the poly-A tails at the end were omitted. Cufflinks[21] v2.2.1 was used to assign the Illumina reads on the ERCC transcripts, with default parameters, and produce FPKM values for every transcript. The GTF file was created manually. The number of Illumina fragments aligned is presented in Supplementary Table S5A.

Except for the FPKM values we also used TPM values after taking the Cufflinks produced FPKM values and transforming them in TPM values as follows[28]:

$$\mathrm{TPMi} = \frac{\mathrm{FPKMi}}{\sum_{i=1}^{n} \mathrm{FPKMi}} * 10^{6}$$

where i corresponds to the different transcripts used and n is the total number of transcripts used. In the ERCC case n=92. From the above equation the TPM method keeps the relative proportion of the FPKM values constant but assigns the values on a different scale. Due to this the correlation values from the comparison between the estimated transcript expression from Illumina and from either the ONT

MinION, the PacBio RS II or the Ambion RNA values, when the FPKM or the TPM method is used, are exactly the same.

III. Simulating fragments from the ERCC transcript database

We followed a similar approach like the one used by Robert et al[6]. We used the "wgsim" module (https://github.com/lh3/wgsim) to simulate random reads from the ERCC transcript sequences. The "wgsim" module creates random fragments from a normal distribution of length fragments with a user defined specific average length and standard deviation. The length of the fragments, at the bulk simulation level, is also user defined and fixed. Our fragment size distribution has a heavy tail (Supplementary Fig. S20) which indicates that modelling the fragment size with a normal distribution is not correct. Additionally, the length of the Illumina sequenced read 1 and read 2 after adaptor removal, is variable between 30-150 bp. In order to simulate accurately the fragment size and read length distributions we used paired end reads where both reads aligned on the same ERCC transcript. Then for each one of these paired end reads we used the "wgsim" module to extract one random fragment with the following properties:

- The simulated fragment is produced from the same ERCC transcript as the one where the specific pair aligns to.
- The simulated length of "read 1" and "read 2" is the same as the original length of the "read 1" and "read 2" for this specific pair.

- The length of the simulated fragment is the same as the aligned length of the specific pair as derived from the SAM file (TLEN field of the Sequence Alignment/Map Format Specification document; https://samtools.github.io/hts-specs/SAMv1.pdf).

This approach also takes care of the different relative abundances of the ERCC transcripts.

IV. Barcoding the ERCC cDNA population

Poly-T priming and template switching enabled the addition of sequences to both the 3' and 5' ends of a cDNA molecule during reverse transcription. This permitted to rescue a number of 3' and 5' end fragments of cDNAs that can be used for molecular counting. Additionally, it permitted the introduction of Unique Molecular Identifiers (UMI) [29] to cDNA during reverse transcription which helped to account for PCR biases.

In order to rescue the 3' and 5' end fragments of cDNAs that can be used for molecular counting we used the following approach. The Illumina flow cell has two sequences, the "complementary P5" and the "complementary P7" sequence that are covalently attached on the flow cell surface and are used to hybridise with the "Illumina adaptor" sequences. The Nextera XT tagmentation (Illumina Inc) process fragments full length cDNAs and adds at the ends of each fragment the "Illumina

adaptor” sequences namely the P5 and P7 sequence. However, the tagmentation process cannot add P5 and P7 sequences at the very end of a full length cDNA thus the ends are lost. To prevent this, sequences compatible with the Nextera XT tagmentation i5 PCR primer were added to the ends of the synthesized cDNAs (bold letters in the Supplementary Fig. S30). After the entire tagmentation process the P5 sequences of the 3’ and 5’ end fragments turns them into sequencing ready libraries that would otherwise be lost.

Having the P5 sequence at the 3’ and 5’ ends of a cDNA, also permits sequencing the UMI region with the highest quality bases. The UMI region (region with italics in the Supplementary Fig. S30) is located between the P5 complementary sequence (underlined region in the Supplementary Fig. S30) and the beginning or end of the ERCC molecules. The UMI are 10 bases long. We introduced UMI with pyrimidines [Y] on the 5’ end and purines [R] on the 3’ end of the cDNA molecules. The different nucleotide content permits us to discriminate the 5’ end adaptor from the 3’ end adaptor.

In general, barcoding the RNA molecules permits the identification of the original number of RNA molecules after removing any PCR amplification bias introduced during the cDNA production. In our case because the MinION platform cannot accurately read the barcode, we were only interested in comparing the estimated cDNA abundance between the MinION and Illumina platforms. Because the

MinION platform sequences directly the cDNA molecules whereas the Illumina platform sequences the cDNA fragments after tagmentation we were only interested in correcting the fragment duplication introduced after the tagmentation PCR amplification. For this we used the UMI barcodes present on each adaptor molecule. The number of different barcodes encoded with our degenerate UMI (YYYYYYYYNN or AAAAAAAANN) is 4096. If the Nextera XT tagmentation enzyme is able to tagment the DNA sequence of a specific ERCC type randomly, a large number of different 5' end and 3' end fragments will be created and the chances of getting the same fragment from two different molecules with the same UMI is small given our sequencing depth. Nevertheless, the Nextera XT tagmentation enzyme shows considerable bias and some fragments are more frequent than others. Because we only have 4096 UMIs this will lead in overestimation of the true number of tagmentation PCR duplicates. For this reason, for each fragment we found the number of expected fragments that have the same UMI and appear by chance. We then randomly selected, from the fragments that appear two or more times, an equal number as the ones that appear by chance and we kept all the duplicated copies of them. For the rest of the fragments that appear two or more times we kept only one copy.

The number of 5' end fragments with the same UMI that appear more than two times was calculated as follows. Initially, we define the probability of capturing exactly k copies of a UMI from the same ERCC 5' end fragment (5' end fragments with the same start and end position on the specific ERCC) as follows[30]:

$$P(X = k) = \frac{n!}{k!\,(n-k)!}\left(\frac{1}{m}\right)^{n}\left(1-\frac{1}{m}\right)^{n-k}$$

where n is the number of the specific ERCC 5’ end fragments sequenced and m is the total number of different UMI available (in our case 4096).

Secondly, we define the probability of observing at least 2 copies of the same UMI from different molecules of the same ERCC fragment as follows:

$$P(X \geq 2) = 1 - (P(0) + P(1))$$

Eventually, the expected number of any UMI present more than once per ERCC 5’ end fragment is given from the following [30]:

$$E = m * (1 - (P(0) + P(1)))$$

where m is the total number of different UMI available (in our case 4096).

In Supplementary Table S5C the total number of duplicated 5’ and 3’ end ERCC cDNA fragments is presented along with the expected number of duplicated 5’ and 3’ end fragments by chance.

V. Bioinformatic processing of the UMI barcoded ERCC reads sequenced on the Illumina platform in order to isolate fragments from the 5’ or the 3’ end of the cDNA molecules

To extract the barcodes we applied a procedure similar with other studies[31].

First the Illumina adaptor sequences were trimmed from the fragments using the AdapterRemoval software[32] with the following commands:

#1st step: remove the Illumina adaptors from both R1 and R2 reads

AdapterRemoval --file1 R1.fastq --file2 R2.fastq --minalignmentlength 10000 --minlength 0 --mm 0.03125 --pcr2 CAAGCAGAAGACGGCATACGAGATNNNNNNNNGTCTCGTGGGCTCGGAGATGTGTATAAGAGACAG --pcr1 CTGTCTCTTATACACATCTCCGAGCCCACGAGACNNNNNNNNATCTCGTATGCCGTCTTCTGCTTG

#2nd step: all the R1 reads from the 1st step that were trimmed or they failed to be properly trimmed from the Illumina adaptors are additionally trimmed as follows to remove any sequence left up to the transposon DNA sequence:

AdapterRemoval --file1 R1_reads_truncated_from_previous_step --minalignmentlength 10000 --minlength 0 --pcr1 CTGTCTCTTATACACATCT

#3rd step: all the R2 reads from the 1st step that were trimmed or they failed to be properly trimmed from the Illumina adaptors are additionally trimmed as follows to remove any sequence left up to the transposon DNA sequence:

AdapterRemoval --file1 R2_reads_truncated_from_previous_step --output1 R2_reads_truncated_from_second_trimming_step --minalignmentlength 10000 --minlength 0 --pcr1 CTGTCTCTTATACACATCT

#4th step: all the R2 reads from the 3rd step that were trimmed or they failed to be properly trimmed from the Illumina adaptors are additionally trimmed as follows to remove any sequence left up to the sequence from the reverse transcription adaptors.

AdapterRemoval --file1 R2_reads_truncated_from_second_trimming_step --minalignmentlength 10000 --minlength 0 --pcr1 GACGCTGCCGACGA

If the length of the remaining R1 or R2 read was more than 30bp then the read was kept.

We then used the R1 reads to find the ones that have the adaptor sequence. For this we used bowtie v4.8.0[33] to align, on the R1 reads, the adaptor sequence present before the UMI position. We call this sequence "initial part of the adaptor sequence". The full length sequence of the "initial part of the adaptor sequence" and truncated versions of it were aligned as follows:

1st step: The following subsequences of the "initial part of the adaptor sequence" were aligned with maximum 3 mismatches against the R1 reads by using bowtie (parameters: -k 100000000 -f -n 3 -e 150 --seedlen 50) :

GCGTCAAGCAGTGGTATCAACGCAGAGT, CGTCAAGCAGTGGTATCAACGCAGAGT, GTCAAGCAGTGGTATCAACGCAGAGT, TCAAGCAGTGGTATCAACGCAGAGT, AAGCAGTGGTATCAACGCAGAGT, AGCAGTGGTATCAACGCAGAGT, GCAGTGGTATCAACGCAGAGT, CAGTGGTATCAACGCAGAGT

2nd step: The following subsequences of the "initial part of the adaptor sequence" were aligned with maximum 2 mismatches against the R1 reads by using bowtie (parameters: -k 100000000 -f -n 2 -e 150 --seedlen 50) :

AGTGGTATCAACGCAGAGT, GTGGTATCAACGCAGAGT, TGGTATCAACGCAGAGT, GGTATCAACGCAGAGT, GTATCAACGCAGAGT, TATCAACGCAGAGT, ATCAACGCAGAGT, TCAACGCAGAGT, CAACGCAGAGT

3rd step: The following subsequences of the “initial part of the adaptor sequence” were aligned with 1 mismatch against the R1 reads by using bowtie (parameters: -k 100000000 -f -n 2 -e 150 --seedlen 50):

AACGCAGAGT

4th step: The reads that were kept as having the 5’ adaptor where the ones where the truncated versions of the “initial part of the adaptor sequence” were able to align as sense strand from the beginning of the Illumina 5’ fragment. For every read only the position of the full length or the longest from the truncated versions of the “initial part of the adaptor sequence” adaptor were accepted as indicative of the position of the “initial part of the adaptor sequence”. The next 10 nucleotides after the “initial part of the adaptor sequence” were kept as the UMI barcode. Additionally, nucleotides between the position 10 and 15 downstream of the “initial part of the adaptor sequence” were kept as the “additional adaptor sequence”.

5th step: An Illumina fragment was kept as a 5’ end fragment only if: 1) the “additional adaptor sequence” corresponded to “ACGCGGG”, 2) the first 8 nucleotides of the UMI barcode sequence was only “C/T” and 3) the Phred quality score of all the 10 nucleotides of the barcode was more than 17. If the Illumina fragment was assigned as a 5’ end fragment, a series of “G” nucleotides after the “additional adaptor sequence” were removed (these correspond to the “C” nucleotides added from the terminal transferase activity of the SuperScript III reverse transcriptase). Additionally, in the Illumina sequences an extra sequence was noticed before the beginning of the ERCC sequences. As this sequence was not in the reference database (“AATTC”), it was additionally removed. If the remaining sequence was more or equal than 25 nucleotides it was kept as characteristic of the ERCC reference database.

6th step: An Illumina fragment was kept as a 3’ end fragment only if: 1) the “additional adaptor sequence” corresponded to “ACTTT”, 2) the first 8 nucleotides of the UMI barcode sequence were only “A/G” and 3) the Phred quality score of all the 10 nucleotides of the barcode was more than

17. Again if the remaining sequence was more or equal than 25 nucleotides in length, it was kept as characteristic of the ERCC sequence.

7th step: The analysis of the R2 reads was done similarly. In the case of reads with polyA stretches, if the non-poly A part of the read was less than 15 nucleotides the read was discarded.

8th step: For each R1 and R2 reads where the adaptors were found, their respective R2 and R1 pairs were selected. The bowtie2[27] v2.1.0 aligner with default parameters (-k 1) was used to align the files. As the bowtie2 can softclip the reads, reads were kept only if they were able to align as pairs with mappable fragment length of more than 30 bp. Additionally for the reads derived from the 3' adaptor we observed that depending on the size distribution of the fragments from the tagmented cDNA library, the R2 file is more probable to align than the R1 file (Supplementary Table S5). This is due to the fact the R2 reads usually lack the poly-A/poly-T tail or they have a part of it and the remaining sequence is adequate for alignment. In this case, the R2 reads were kept only if they were able to align with at list 25 nucleotides in length.

VI. Considerations during the ONT MinION sequencing of the ERCC cDNA population

The amount of ERCC cDNA material in every step of the ONT library preparation for the different experiments is presented in Supplementary Table S3. In Supplementary Table S3 the ug of DNA left after each cleaning step is also presented. In Supplementary Table S6 the amount of DNA loaded on the flow cell is also indicated along with the time intervals of the DNA loading. Additionally, the time intervals where the machine was restarted (“remux” time) and thus a new batch of pores was selected from the functional ones is also presented[1]. The ideal

time intervals for restarting and reloading the machine with DNA is under investigation from the ONT MinION community. The time intervals presented here is the ones used while we familiarized ourselves with the platform. The MinION Analysis and Reference Consortium has provided some guidelines about the restarting and reloading intervals[1]. From our experiments we also noticed that the yield (megabases) and the pore occupancy at the first 4 hours of the MinION flow cell runs is proportional to the amount of DNA loaded on the MinION machine (Supplementary Fig. S32A, B).

An important point for consideration in our experiments is the amount of DNA used at the ONT MinION adaptor ligation step. The ONT recommended ratio of "MinION adaptors:DNA" at the adaptor ligation step is "10:1", which is the same as the ratio used in all the other sequencing platforms that involve ligation of adaptor sequences on the DNA of interested. As the total yield from a MinION flowcell depends on the amount of DNA loaded (Supplementary Fig. S32 and Table 1) we tried to recover as much DNA as possible from one library preparation reaction (each kit can be used for maximum four library preparations). As the library preparation kit used here (SQK-MAP0005, SQK-MAP0004) involved the use of magnetic beads to separate DNA molecules that have the hairpin adaptor from the ones that do not have it, we tried to increase the DNA yield after the enrichment step. For this we used a ratio of "MinION adaptors:DNA" equal to "1:1". Indeed as we see in the case of the ERCC experiment number 4 we recovered 15 times more DNA (258 ngs versus 17.5 ngs in ERCC experiment number 3) whereas in

the case of the H3K293 cDNA we recovered 7 times more (125 ngs vs 17.5 ngs in ERCC experiment number 3, the 125 ngs were calculated from the extrapolation of the 30 ngs recovered in 6 ul of library solution up to a potential 25 ul of library solution) (Supplementary Table S3). The 1:1 “MinION adaptors:DNA” ratio does not affect the recovery of high quality 2D reads as the total yield of high quality 2D reads sequenced, in the case of the ERCC experiment 2, 3, 4 was proportional to the total amount number of reads sequenced (14.8% in ERCC experiment number 4 , 8% in ERCC experiment number 2, 5.36% in ERCC experiment number 3 , % of the number of aligned high quality 2D reads to the total number of sequenced reads from Table 1). In the case of the HEK-293 cDNA the complexity of the library has probably affected the recovery of high quality 2D reads proportional to the total number of reads sequenced (0.9% of the total number of sequenced reads from Table 1).

VII. Alignment of the ONT MinION reads

The r7 2D basecalling pipeline (r7 flow cell) and r7.X 2D basecalling rev 1.12 or 1.16 pipeline (r7.3 flow cell) were used to process the raw signal (Table 1). The basecalled sequences were extracted as fastq files from HDF5 files using poRe[34] v0.6 . Multiple aligners, from the ones presented in the literature, were used for aligning on the reference ERCC database (Supplementary Table S2). For each aligner the parameters presented on Supplementary Table S2 were used whereas

any other parameter was left as default. The ERCC reference database contained only the ERCC sequence as provided from the manufacturer without the reverse transcription adaptors and the poly-A tail. For every aligner only reads that aligned with at least 50% of their sequenced length were kept. If the reads aligned in more than one position, the alignment with the highest score was kept. The results from each aligner were tested for their agreement with the expected cDNA abundance (Supplementary Fig. S11 and Supplementary Fig. S12), number of aligned reads (Supplementary Fig. S13A), alignment length (Supplementary Fig. S13B,C) and alignment accuracy (Supplementary Fig. S13D). Different implementations of the LAST aligner ("MarginAlign_1","LAST_1", "LAST_3" and "LAST_4" in Supplementary Table S2) gave the best agreement between the expected and observed cDNA abundance (r_{p_raw}=0.99, r_{s_raw}=0.7-0.75, r_{p_log10}=0.96-0.97, r_{s_log10}=0.91-0.94), an adequate number of aligned reads ($n_{MarginAlign\_1}$=2581, n_{LAST_1}=2248, n_{LAST_3}=2260, n_{LAST_4}=2276), a high identity (64.1% for "MarginAlign_1", 67.4% for "LAST_1", 67.4% for "LAST_3" and 67.3% for "LAST_4") and alignment length (75.2% for "MarginAlign_1", 75.8% for "LAST_1", 75.7% for "LAST_3" and 75.6% for "LAST_4" relative to the sequenced length which represent the ERCC sequence with the two adaptors and a small number of nucleotides from the ONT MinION adaptors). The LAST aligner with the parameters of the "LAST_1" category (Supplementary Table S2) was eventually selected. The basecalling accuracy for all the ONT MinION experiments is presented in Supplementary Fig. S1.

VIII. Subsampling the ONT MinION low quality template reads

To support our finding that the low quality template reads are in better agreement with the expected cDNA abundance from the Illumina molecular counts, we examined whether it may be originating from the difference in the number of either the high quality template reads, the low quality complement reads or the low quality 2D reads (Supplementary Fig. S9*,* Supplementary Fig. S10).

In order to test for this possibility, we subsampled from the aligned ONT MinION low quality template reads equal number as the number of aligned reads present in either the high quality template reads category, the low quality complement reads category or the low quality 2D reads category. For each one of these three categories we subsampled 300 groups from the low quality template reads. Then, for each one of the 300 groups the agreement between the expected cDNA abundance from the Illumina molecular counts and the observed cDNA abundance from the group was calculated. We used three metrics of agreement. We used the Pearson correlation as a non-rank based metric. We also used rank based metrics. As the rank based metrics are less sensitive than the non-rank based ones we used two different metrics, the Spearman correlation and the Kendall correlation. The Spearman and the Kendall correlations examine concordance of rank orders. The Pearson correlation examines concordance of cDNA abundance.

If the concordance of either the high quality template reads category, the low quality complement reads category or the low quality 2D reads category with the expected cDNA abundance (Illumina molecular counts) is significantly different from the one of the low quality template reads category, then the correlation values of either the high quality template reads category, the low quality complement reads category or the low quality 2D reads category (we call these values “sample correlation values”) must be present at the extremes of the distribution of the correlation values from the 300 subsampled groups. The previous assessment is carried out independently for each one of the Pearson, Spearman or Kendall correlation metrics (Supplementary Fig. S9, Supplementary Fig. S10). To have a measure of how extreme the sample correlation values are, we fitted a gamma distribution on the distribution of the correlation values from the 300 subsampled groups and a p-value was calculated based on the gamma distribution as follows:

The gamma distribution was fitted on the data with the “fitdistrplus”[35] library in R. The command line is:

```
#First the parameters of the gamma distribution are calculated from the 300 correlation values of the subsampled groups for either the Pearson, Spearman or Kendall correlation metrics.

distribution <- fitdist( data, "gamma", method="mge" )
```

Secondly, we calculate the probability value for the sample correlation value from the gamma distribution of either the Pearson, Spearman or Kendall correlation metrics. This value corresponds to the 1-tail p-value. The parameters of the gamma distribution are taken from the previous command.

```
probability_sample_correlation <- pgamma ( sample_coeffcient, distribution$estimate[[1]], rate=
distribution $estimate[[2]] )
one_tail_p_value <- probability_sample_correlation
```

#Thirdly, we define the complement of the previous event as follows:

```
complement_event <- ( 1- probability_sample_correlation )
```

#Then the 2-tail p-value is calculated as follows:

```
two_tailed_p_value <- 2 * min ( probability_sample_correlation, complement_event )
```

The 2-tail p-value indicates the distance of a specific sample correlation value (for either the Pearson, Spearman or Kendall correlation metrics) from the center of the distribution of the values, for the same correlation metric, from the 300 subsampled groups (p-value close to 0 is further away from the center). The concordance of either the high quality template reads, the low quality complement reads or the low quality 2D reads with the Illumina molecular counts is different

from the concordance of the low quality template reads with the Illumina molecular counts, if at least one of the correlation metrics have 2-tail p-values <0.05. If the rank order correlations (Spearman, Kendall) do not show any significant difference (2-tail p-value >0.05) but the Pearson correlation does so (2-tail p-value <0.05), then the values of the examined groups differ in their magnitudes rather than their ranks. If the reverse happens then the examined groups differ in their ranks.

For the cases where at least one of the 2-tail p-values is less than 0.05, the corresponding 1-tail p-value indicates whether the specific sample correlation value is in better concordance with the Illumina molecular counts than the correlation of the low quality template reads group (p-value close to 0, the sample correlation value is greater than the 75th percentile of the distribution of the values, for the same correlation metric, from the 300 subsampled groups). If this specific 1-tail p-value is close to 1, it indicates that the examined sample correlation value is in worse concordance with the Illumina molecular counts than the correlation of the low quality template reads group (the sample correlation value is lower than the 25th percentile of the distribution of the values, for the same correlation metric, from the 300 subsampled groups).

In cases where all the 2-tail p-values are less than 0.05 or at least one of the rank-based metrics (Spearman, Kendal) and the Pearson correlation metric have 2-tail p-values <0.05, then we give priority to the Pearson correlation metric and we

examine the 1-tail p-value only from the Pearson correlation metric. In this case the Spearman and Kendall correlation metrics are not taken into account. If the 1-tail p-value, of the Pearson correlation metric, is more than 0.05 then the low quality template reads are in better concordance with the Illumina molecular counts otherwise if the 1-tail p-value is less than 0.05 then one of the read groups (the high quality template reads group, the low quality complement reads group, the low quality 2D reads group) is in better concordance with the Illumina molecular counts.

In Supplementary Table S1 the p-values presented in Supplementary Fig. S9 and Supplementary Fig. S10 are recorded. Both the 2-tail p-values group (Supplementary Table S1A) or the 1-tail p-values group (Supplementary Table S1B) from the Pearson, Spearman and Kendall sample/subsample comparisons are presented. The hypotheses that each p-value group is testing are also indicated. The accepted hypothesis from each comparison, based on the selection criteria presented in this section, is indicated with the blue color boxes in Supplementary Table S1**.**

In Supplementary Table S1A we see that in the majority of cases tested for the ERCC experiments number 1, 2, 3, 4 (9 out of 11 cases; tests where at least one of the 2-tail p-values is less than 0.05) the cDNA abundance of the ERCC transcripts estimated from either the high quality template reads, the low quality

complement reads or the low quality 2D reads, always differs in its magnitude from the one estimated from the low quality template reads. In some cases (5 out of 9 cases; tests where at least one of the 2-tail p-values, from the Spearman or Kendall correlation metrics, is less than 0.05) the rank order of the ERCC cDNA abundance is also affected something that is not unexpected. When we examined the 1-tail p-values (Supplementary Table S1B) all of them showed that the low quality template reads from the ERCC experiments 2, 3, 4 or all the template reads from the ERCC experiment 1 were in better concordance with the Illumina molecular counts than either the high quality template reads, the low quality complement reads or the low quality 2D reads (1-tail p-value for the Pearson correlation metric >0.05). In the case of the high quality template reads from the ERCC experiment 3, the 2-tail p-value of the Pearson correlation metric (~0.22), although not statistically significant, points to a lower concordance with the Illumina molecular counts compared to the low quality template reads. The barplot distribution is also indicative to this (Supplementary Table S1A). In the case of the low quality 2D reads from the ERCC experiment 3, the 2-tail p-values from the Spearman and Kendall correlation metrics (~0.1, ~0.13 respectively), although not statistically significant, again point to a lower concordance, in the rank order, with the Illumina molecular counts compared to the low quality template reads. The low number of MinION reads produced in the case of the ERCC experiment 3 might not give a clear picture as in the other ERCC experiments but the discussed trends support the observation that the low quality template reads are in better concordance to the Illumina molecular counts, as was observed in all the other ERCC experiments.

IX. HEK-293 cDNA production

The isolation of total RNA and the cDNA production was based on the Single-Cell cDNA Library preparation protocol for mRNA Sequencing for the Fluidigm C1 machine (PN 100-7168 I1)[36] as follows: Synthetic RNA transcripts number #1, #4 and #7 from the “ArrayControl RNA Spikes kit” (#AM1780, Ambion, Thermo) were diluted in RNA storage solution as follows. For Synthetic RNA transcript number #1, 1.5 ul of stock was diluted in 148.5 ul of RNA storage solution. For Synthetic RNA transcript number #4, 1.5 ul of stock was diluted in 12 ul of RNA storage solution. For Synthetic RNA transcript number #7, 1.5 ul of stock was diluted in 13.5 ul of RNA storage solution. All the solutions were vortexed briefly and afterwards 1.5 ul from the diluted Synthetic RNA transcript number #7 solution was transferred to the diluted Synthetic RNA transcript number #4 solution and mixed briefly (we call this solution “4, 7 mix”). Then another 1.5 ul from the “4, 7 mix” was transferred to the Synthetic RNA transcript #1 solution and the final solution was mixed briefly (we call this solution “1, 4, 7 mix”) and stored at -80°C until use. Upon defrost 1 ul from the “1, 4, 7 mix” was diluted in 99 ul of Loading Reagent (Fluidigm) (we call this solution “final 1, 4, 7 mix”). Afterwards the solution for the cell lysis and the poly-A hybridization of the first strand cDNA synthesis primer, was created as follows (1 ul from the “final 1, 4, 7 mix”, 0.5 ul RNase Inhibitor (Clontech), 7 ul 3′ SMART CDS Primer IIA (Clontech), 11.5 ul Clontech Dilution Buffer) and briefly mixed (we call it “lysis solution”). We then extracted RNA from 200 HEK-293 cells.

For this, 3 ul from the “lysis solution” was added on the pellet of 200 HEK-293 cells. Then the cell lysis and the the poly-A hybridization step was performed after incubating the tube with the following thermocycler parameters 72°C for 3 min, 4°C for 10 min, 25°C for 1 min. The first and second strand cDNA synthesis solution was created as follows: 1.2 ul of Loading Reagent (Fluidigm),11.2 ul of 5X First-Strand Buffer (RNase-free) (Clontech), 1.4 ul of DTT (Clontech), 5.6 ul of dNTP Mix (dATP, dCTP, dGTP, and dTTP, each at 10 mM) (Clontech), 5.6 ul of SMARTer IIA Oligonucleotide (Clontech), 1.4 ul of RNase Inhibitor (Clontech), 5.6 ul of SMARTScribe Reverse Transcriptase (Clontech) (we call it “Reverse Transcription (RT) Reaction Mix”). The cDNA synthesis was performed by addition of 4 ul “Reverse Transcription (RT) Reaction Mix” to the 3 ul lysis reaction. The cDNA synthesis was performed with the following thermocycling parameters: 1 cycle of [50 ^{o}C for 90 min], 10 cycles of [55 ^{o}C for 2 min, 50 ^{o}C for 2 min], 5 cycles of [60 ^{o}C for 2 min, 55 ^{o}C for 2 min], 1 cycle of [70 ^{o}C for 15 min]. To amplify the cDNA, 63 ul of cDNA amplification mix (44.45 ul of PCR-Grade Water (Advantage 2 PCR Kit, Clontech), 7 ul of 10X Advantage 2 PCR Buffer (not SA, short amplicon) (Advantage 2 Kit, Clontech), 2.8 ul of 50X dNTP Mix (Advantage 2 PCR Kit, Clontech), 2.8 ul of IS PCR primer (Clontech SMARTer Kit), 2.8 ul of 50X Advantage 2 Polymerase Mix (Advantage 2 PCR Kit, Clontech), 3.15 ul of Loading Reagent (Fluidigm)) was added to 7 ul of first strand cDNA synthesis reaction. This 70 ul cDNA PCR reaction was amplified for 21 cycles with the following thermocycler parameters: 1 cycle of [95°C for 1 min], 5 cycles of [95°C for 20 seconds, 58°C for 4 minutes, 68°C for 6 minutes], 9 cycles for [95°C for 20

seconds, 64°C for 30 seconds, 68°C for 6 minutes], 7 cycles for [95°C for 30 seconds, 64°C for 30 seconds, 68°C for 7 minutes], 1 cycle for [72°C for 10 minutes]. The amplified product was subsequently cleaned with 0.9 X sample volume AMPure XP beads, eluted in H_20 and the cDNA size distribution was profiled on a Caliper LabChip GXII (PerkinElmer).

X. ONT MinION sequencing of the HEK-293 cDNA population

The HEK-293 cDNA library was sequenced with a version r7.3 MinION flow cell. The ONT MinION Genomic DNA Sequencing Kit reagents (SQK-MAP0005) and the 2D cDNA sequencing protocol were used to prepare the libraries. The amount of starting material used is presented in Supplementary Table S3. In each case the cDNA was end-repaired in a 50 ul reaction containing: 0.5 ug of a 40 ul solution cDNA, 5 ul of 10X End-repair buffer (from NEBNext End Repair Module), 2.5ul End-repair enzyme mix (from NEBNext End Repair Module), nuclease-free water (2.5 ul). It was then incubated at 25°C in a thermocycler for 30 minutes. Afterwards we added 1X sample volume Ampure XP beads to the End-Repair reaction. The solution was mixed by pipetting and the cDNA was allowed to bind to the beads by rotating for 5 minutes on a HulaMixer (Thermo). Then the beads were pelleted on a magnet, the supernatant was aspired off and the beads, while they stayed on the magnet, were washed twice with 200 µl of freshly prepared 70% ethanol. Then the tube was centrifuged to collect residual liquid at bottom of tube and the residual wash solution was aspirated. The cDNA was eluted from the beads by re-

suspending the beads in 12.5 ul of 10 mM Tris-HCl pH 8.5 and the beads were incubated for 5 minutes at room temperature. The isolated cDNA was quantified on the Qubit fluorimeter. For the subsequent dA tailing step all the eluted cDNA was used in the following reaction cDNA library (430 ug), 1.5 ul 10x dA-tailing buffer (from NEBNext dA-Tailing Module), 1 ul dA-tailing enzyme (from NEBNext dA-Tailing Module). Subsequently, the cDNA was cleaned with the Ampure XP beads as described above and eluted in 7.5 ul of H20. In the ligation reaction the DNA was mixed with the following reagents: 7.5 ul of dA-tailed DNA, 2.5 ul Adapter Mix, 2.5 ul HP adapter, 12.5 ul Blunt/TA ligase Master Mix. The cDNA library was then enriched for sequences that bear the hairpin as described for the ERCC cDNA library with the difference that all the volumes were scaled down by 1/4. The amount of HEK-293 cDNA material in every step of the ONT library preparation method is presented in Supplementary Table S3.

The introduction of transcripts of known length (RNA spikes) are recommended to monitor the performance of the MinION flow cell (Supplementary Fig. S16) Additionally adaptor sequences at the beginning and end of the DNA molecules are also necessary in order to be able to recognize in cDNA molecules where only the template sequence was sequenced whether the molecules are full length

XI. Identification of the similarity in the TSS and TES positions between the PacBio RS II and ONT MinION platforms for the HEK-293 cDNA library.

Regions of low basecalling quality can usually be aligned only if they are flanked from regions of high quality. As a consequence low basequalling quality regions at the beginning and end of the molecules usually will not be able to align. For this reason, in order to accurately identify the position of the TSS and TES, we corrected the beginning position of the aligned part of the ONT MinION reads as follows.

We focused only on the 5' and 3' ends on which we can identify the position of the adaptor sequence. The adaptor sequence was aligned on each MinION read with the following parameters:

#1st step: The adaptor sequence (AAGCAGTGGTATCAACGCAGAGTAC) was aligned with the lastal module, from the LAST package[7], with the following parameters:

lastal -Q0 -k1 -m1000000 -a1 -j4 -g1.0 -T0 -w0 -s2 -e 10

#2nd step:

The adaptor was accepted as aligning on the 5' end of the sequence only if: 1) It aligns as sense strand, 2) The beginning of the adaptor sequence is inside the beginning 15% of the sequence on the reference database, 3) At least 80% of the adaptor sequence aligns.

#3rd step:

The adaptor was accepted as aligning on the 3' end of the sequence only if: 1) It aligns as antisense strand, 2) The beginning of the adaptor sequence is inside the last 20% of the sequence on the reference database, 3) At least 80% of the adaptor sequence aligns.

In case that the 3' end of the adaptor sequence is partially aligned we extended the aligned position on the MinION read up to as many 3' end nucleotides as the ones that were not able to align. The MinION reads were then aligned against the same database as the one used for the Illumina HEK-293 cDNA fragments. The length of the non aligned sequence between the position of the adaptor and the beginning or end of the aligned part of the MinION reads was added on the beginning or end positon of the aligned MinION read in order to have the corrected TSS or TES. The same approach was applied for the PacBio reads. In the case of the Illumina platform, the original 5' end fragments and respectively the 3' end fragments of the cDNA molecules produced with the Clontech SMARTer Kit and the Nextera XT tagmentation method (Illumina Inc), will frequently be lost as the adaptor lack sequences compatible with the Nextera XT tagmentation i5 PCR primer.

XII. Alignment of the HEK-293 Illumina HiSeq 2500 cDNA reads

Raw pair end sequencing reads were obtained from the Illumina HiSeq 2500 platform. They were trimmed using Trimmomatic[37] version 0.33, to a minimum length of 30 nucleotides. Nextera library adapters were removed in palindrome

mode. A minimum Phred quality score of 30 was required for the 3'end. Alignment was performed on the UCSC hg19 reference human genome downloaded from the Illumina iGenomes web site[38], using Tophat[39] version 2.0.13. Removal of duplicate reads was performed using Picard v.1.128 (http://picard.sourceforge.net). Estimation of expression levels for different known isoforms and genes was inferred using Cufflinks[21] version 2.2.1 by using gene models from NCBI Homo sapiens Annotation Release version 104.

For the comparison with the PacBio RS II and ONT MinION reads we used the "gffread" utility from the Cufflinks software package (command line: gffread -w transcripts.fa -g /path/to/genome.fa transcripts.gtf) to extract FASTA files with spliced exons corresponding to the aforementioned gene models GTF annotations (we call this file "transcriptome fasta file").

For the Kallisto[25] version 0.42.5 and Sailfish[26] version 0.9.2 aligners we aligned against the transcriptome fasta file. For the Kallisto default parameters were used. For the Sailfish default parameters were used with the exception of the kmer size ("-k " parameter) which was reduced down to 31. For the Sailfish the "biasCorrect" parameter was also used.

For the StringTie[24] version 1.2.3 software default parameters were used with the following changes:

- When we deactivated the effective size correction we selected the “-t” option.

For the Cuffilnks software default parameters were used with the following changes:

- When we deactivated the effective size correction we selected the “–no-effective-length-correction” option.

XIII. PacBio RS II processing of raw read files

The PacBio raw data was filtered using SMRT analysis v2.3 patch 4 to produce both raw subreads as well as Circular Consensus Sequencing reads (CCS) in fasta format. These reads were then aligned to the reference using blasr with settings "-bestn 1 -minMatch 11 -maxAnchorsPerPosition 500 -clipping soft -sam". The number of raw and aligned reads are presented in Supplementary Table S7.

For the PacBio RS II reads we define the “longest subread” group and the “Circular Consensus Sequencing” read group as follows. Each molecule in an SMRT cell microwell can be sequenced multiple times around because of the hairpin adapters on each end of the dsDNA producing a continuous sequence (polymerase read).

While sequencing around the molecule and producing the polymerase read, every time an adapter is observed a new subread is started. At the start of sequencing if no adapter sequence is observed a subread is also started there. The sequence after the last observed adapter is also considered as a subread. In the end each microwell can produce multiple subreads but only one polymerase or raw read. To avoid counting the same molecule multiple times we used the longest of the subreads as a representative of the molecule sequenced. Frequently the longest subread corresponds to partially sequenced cDNA molecules. For example in Supplementary Fig. S23A, B we see that 37% and 12% of the sequenced PacBio “longest subreads” do not reach the TSS or the TES accordingly, in contrast to the PacBio CCS reads.

Multiple raw subreads from the same molecule can be used to create a higher quality sequence for the sequenced molecule. For example if every base is covered by two or more subreads a two pass circular consensus read (CCS) can be called.

XIV. Bias quantification

To quantify the potential bias of the GC content or length on the ERCC cDNA sequencing from either the ONT MinION or Illumina HiSeq 2500 we followed an approach similar to the one presented in another study with ERCC cDNAs[40].

We used the R function "lm" to fit the regression models and the nlme (version 3.1-122) R package to compute the BIC score. The models used are:

$$Y = bo + b1 * L + e$$

$$Y = bo + b1 * G + e$$

$$Y = bo + b1 * L + b2 * G + e$$

where Y is the log 2 ratio of the observed to expected abundance for each ERCC transcript, L and G denote the length and GC content of each ERCC transcript; b0, b1, b2, are coefficients and e is residual error. ANOVA tests for all models were performed in R.

References

1 Ip, C. *et al. MinION Analysis and Reference Consortium: Phase 1 data release and analysis [version 1; referees: awaiting peer review]*. Vol. 4 (2015).

2 Bolisetty, M. T., Rajadinakaran, G. & Graveley, B. R. Determining exon connectivity in complex mRNAs by nanopore sequencing. *Genome biology* **16**, 204, doi:10.1186/s13059-015-0777-z (2015).

3 Ammar, R., Paton, T. A., Torti, D., Shlien, A. & Bader, G. D. Long read nanopore sequencing for detection of HLA and CYP2D6 variants and haplotypes. *F1000Research* **4**, 17, doi:10.12688/f1000research.6037.1 (2015).

4 Benitez-Paez, A., Portune, K. J. & Sanz, Y. Species-level resolution of 16S rRNA gene amplicons sequenced through the MinION portable nanopore sequencer. *GigaScience* **5**, 4, doi:10.1186/s13742-016-0111-z (2016).

5 Greninger, A. L. *et al.* Rapid metagenomic identification of viral pathogens in clinical samples by real-time nanopore sequencing analysis. *Genome medicine* **7**, 99, doi:10.1186/s13073-015-0220-9 (2015).

6 Robert, C. & Watson, M. Errors in RNA-Seq quantification affect genes of relevance to human disease. *Genome biology* **16**, 177, doi:10.1186/s13059-015-0734-x (2015).

7 Frith, M. C., Hamada, M. & Horton, P. Parameters for accurate genome alignment. *BMC bioinformatics* **11**, 80, doi:10.1186/1471-2105-11-80 (2010).

8 Benitez-Paez, A., Portune, K. & Sanz, Y. *Species level resolution of 16S rRNA gene amplicons sequenced through MinIONTM portable nanopore sequencer*. (2015).

9 Quick, J. *et al.* Rapid draft sequencing and real-time nanopore sequencing in a hospital outbreak of Salmonella. *Genome biology* **16**, 114, doi:10.1186/s13059-015-0677-2 (2015).

10 Karlsson, E., Larkeryd, A., Sjodin, A., Forsman, M. & Stenberg, P. Scaffolding of a bacterial genome using MinION nanopore sequencing. *Scientific reports* **5**, 11996, doi:10.1038/srep11996 (2015).

11 Madoui, M. A. *et al.* Genome assembly using Nanopore-guided long and error-free DNA reads. *BMC genomics* **16**, 327, doi:10.1186/s12864-015-1519-z (2015).

12 Kilianski, A. *et al.* Bacterial and viral identification and differentiation by amplicon sequencing on the MinION nanopore sequencer. *GigaScience* **4**, 12, doi:10.1186/s13742-015-0051-z (2015).

13 Bolisetty, M., Rajadinakaran, G. & Graveley, B. *Determining Exon Connectivity in Complex mRNAs by Nanopore Sequencing*. (2015).

14 Altschul, S. F., Gish, W., Miller, W., Myers, E. W. & Lipman, D. J. Basic local alignment search tool. *Journal of molecular biology* **215**, 403-410, doi:10.1016/S0022-2836(05)80360-2 (1990).

15 Greninger, A. L. *et al. Rapid metagenomic identification of viral pathogens in clinical samples by real-time nanopore sequencing analysis*. (2015).

16 He, S. *et al.* Validation of two ribosomal RNA removal methods for microbial metatranscriptomics. *Nature methods* **7**, 807-812, doi:10.1038/nmeth.1507 (2010).

17 Zhao, M., Lee, W. P., Garrison, E. P. & Marth, G. T. SSW library: an SIMD Smith-Waterman C/C++ library for use in genomic applications. *PloS one* **8**, e82138, doi:10.1371/journal.pone.0082138 (2013).

18 Szalay, T. & Golovchenko, J. A. *A de novo DNA Sequencing and Variant Calling Algorithm for Nanopores*. (2015).

19 Chaisson, M. J. & Tesler, G. Mapping single molecule sequencing reads using basic local alignment with successive refinement (BLASR): application and theory. *BMC bioinformatics* **13**, 238, doi:10.1186/1471-2105-13-238 (2012).

20 Jain, M. *et al.* Improved data analysis for the MinION nanopore sequencer. *Nature methods* **12**, 351-356, doi:10.1038/nmeth.3290 (2015).

21 Trapnell, C. *et al.* Transcript assembly and quantification by RNA-Seq reveals unannotated transcripts and isoform switching during cell differentiation. *Nature biotechnology* **28**, 511-515, doi:10.1038/nbt.1621 (2010).

22 Li, B., Ruotti, V., Stewart, R. M., Thomson, J. A. & Dewey, C. N. RNA-Seq gene expression estimation with read mapping uncertainty. *Bioinformatics* **26**, 493-500, doi:10.1093/bioinformatics/btp692 (2010).

23 Angelini, C., De Canditiis, D. & De Feis, I. Computational approaches for isoform detection and estimation: good and bad news. *BMC bioinformatics* **15**, 135, doi:10.1186/1471-2105-15-135 (2014).

24 Pertea, M. *et al.* StringTie enables improved reconstruction of a transcriptome from RNA-seq reads. *Nature biotechnology* **33**, 290-295, doi:10.1038/nbt.3122 (2015).

25 Bray, N. L., Pimentel, H., Melsted, P. & Pachter, L. Near-optimal probabilistic RNA-seq quantification. *Nature biotechnology* **34**, 525-527, doi:10.1038/nbt.3519 (2016).

26 Patro, R., Mount, S. M. & Kingsford, C. Sailfish enables alignment-free isoform quantification from RNA-seq reads using lightweight algorithms. *Nature biotechnology* **32**, 462-464, doi:10.1038/nbt.2862 (2014).

27 Langmead, B. & Salzberg, S. L. Fast gapped-read alignment with Bowtie 2. *Nature methods* **9**, 357-359, doi:10.1038/nmeth.1923 (2012).

28 Pachter, L. Models for transcript quantification from RNA-Seq. *ArXiv e-prints* **1104** (2011). <http://adsabs.harvard.edu/abs/2011arXiv1104.3889P>.

29 Kivioja, T. *et al.* Counting absolute numbers of molecules using unique molecular identifiers. *Nature methods* **9**, 72-74, doi:10.1038/nmeth.1778 (2012).

30 Fu, G. K., Hu, J., Wang, P. H. & Fodor, S. P. Counting individual DNA molecules by the stochastic attachment of diverse labels. *Proceedings of the National Academy of Sciences of the United States of America* **108**, 9026-9031, doi:10.1073/pnas.1017621108 (2011).

31 Islam, S. *et al.* Quantitative single-cell RNA-seq with unique molecular identifiers. *Nature methods* **11**, 163-166, doi:10.1038/nmeth.2772 (2014).

32 Lindgreen, S. AdapterRemoval: easy cleaning of next-generation sequencing reads. *BMC research notes* **5**, 337, doi:10.1186/1756-0500-5-337 (2012).

33 Langmead, B., Trapnell, C., Pop, M. & Salzberg, S. L. Ultrafast and memory-efficient alignment of short DNA sequences to the human genome. *Genome biology* **10**, R25, doi:10.1186/gb-2009-10-3-r25 (2009).

34 Watson, M. *et al.* poRe: an R package for the visualization and analysis of nanopore sequencing data. *Bioinformatics* **31**, 114-115, doi:10.1093/bioinformatics/btu590 (2015).

35 Delignette-Muller, M. L. & Dutang, C. fitdistrplus: An R Package for Fitting Distributions. *J Stat Softw* **64**, 1-34 (2015).

36 <https://www.fluidigm.com/binaries/content/documents/fluidigm/resources/c1-mrna%E2%80%90seq-pr-100%E2%80%907168/c1-mrna%E2%80%90seq-pr-100%E2%80%907168/fluidigm%3Afile> (

37 Bolger, A. M., Lohse, M. & Usadel, B. Trimmomatic: a flexible trimmer for Illumina sequence data. *Bioinformatics* **30**, 2114-2120, doi:10.1093/bioinformatics/btu170 (2014).

38 *Illumina. iGenomes, ready-to-use reference sequences and annotations*, < https://support.illumina.com/sequencing/sequencing_software/igenome.html> (
39 Trapnell, C., Pachter, L. & Salzberg, S. L. TopHat: discovering splice junctions with RNA-Seq. *Bioinformatics* **25**, 1105-1111, doi:10.1093/bioinformatics/btp120 (2009).
40 Jiang, L. *et al.* Synthetic spike-in standards for RNA-seq experiments. *Genome research* **21**, 1543-1551, doi:10.1101/gr.121095.111 (2011).
