# Supplementary material for: Benchmarking of the Oxford Nanopore MinION sequencing for quantitative and qualitative assessment of cDNA populations
Source: Sci Rep. 2016 Aug 24;6:31602. doi: 10.1038/srep31602 (PMC4995519; doi:10.1038/srep31602)
Supplement: Supplementary Text [file srep31602-s4.doc]

**cDNA sequence of the 92 ERCC transcripts used during the alignment process. For each FASTA record the name corresponds to the following fields separated by spaces: ERCC transcript rank abundance (from the most to the least abundant based on the RNA concentration), ERCC transcript name, number of GC bases of the ERCC transcript, length of the ERCC transcript. In some alignment files provided on the ENA database only the rank abundance number was used as indicative of the ERCC transcript name whereas in others the transcript name itself was used.**

>1 ERCC-00130 486 1059

GCATTTTGAAAATTCTATGGAAGAGCTAGCATCTCTGACGAAAACAGCAGACGGAAAAGTACTGACCAGCGTCACACAAAAACGGAACAGGGCTGACGCCGCTACATATATAGGAAAAGGGAAGGTAGAAGAGCTGAAGGCACTCGTGGAAGAGCTTGAAGCTGATCTCCTCATCTTTAATGATGAACTGTCGCCAAGTCAGCTGAAGTCATTGGCAACAGCAATTGAAGTGAAGATGATTGACCGCACGCAATTGATATTAGATATTTTTGCAAAGCGGGCGAGAACGAGAGAAGGCAAACTTCAAATTGAGCTGGCTCAGCTGCAATATGCACTGCCGCGTCTGACGGGACAAGGGATCAACCTTTCCCGGCAAGGCGGAGGAATTGGGGCAAGAGGTCCCGGGGAAACGAAACTGGAAACCGACCGCCGCCATATCAGAAATCGCATTCATGAAATCAACACACAGCTTTCCACTGTCATTCGCCATAGAAGCCGATACCGTGAAAGAAGAAAGAAAAACGGTGTGCTTCAAATTGCGCTTGTCGGCTATACAAACGCAGGGAAATCAACATGGTTCAACCGCCTGACGAGTGCTGACAGCTATGAAGAAGACCTCCTGTTTGCCACGCTGGACCCGATGACCAGAAAAATGGTCCTGCCAAGCGGCTACAGTGTTCTTCTTTCAGATACAGTAGGATTTATTCAGGATCTTCCGACGACATTGATTGCTGCATTCCGCTCAACGCTTGAGGAAGTAAAAGAAGCGGATTTAATTCTGCATTTAATTGATTCTTCAAATGAGGATTATGCGGGACATGAAAAAACAGTGCTTCGGCTGCTTGAGGAGCTTGAAGCAGATGATATCCCGATGCTGACGGCTTACAATAAACGTGATCAAAAACTGCCTGATTTTATACCGACCGCCGGAAGGGATCACATTATGGTCAGTGCGAAATTTGAGGACGACGCTGCAGCCTTTAAAGAAGCGATTCAGCGCTATTTGCGCCAAGAACTGTTAACGTCTTGAATTCTGGAA

>2 ERCC-00074 181 522

TGGACATTAATTAGGGCTGAAAGCCCTAACTTAATGGACGGGAGGTATCCCAATAGGAGGTTTCCTCCTATGGTTTTCAAAACAATCACCATCATGCTATTAATGATATTAAAATCCCAACTATACCAAAGAATATCCCAATTATCCATAAAACTGTAACTAAGTGAGGCTCTCTCATTGGTTTATACTTCAATATAAGCCTTGGTAGGGATAGATAGCCACCTATATAGTATAGCTTCCCATCTTCTTTGAGAGTTGTTGGTTTATGCTCATCCCTACTCATAACCCCAGCACTTAGATATTTTAAAGAGGCATCTATCACATAAGGCATCATTATAACTAAAAATGGGATATATTCCTTATAAACTACTGCTAAGACAGCTAAGAAAGCTCCAATTGGTAGAGTTCCAACATCTCCTGGAAAAACCTTTGCTGGATATTTGTTAAATATCAATAGCCCTAAATAGGATGCAGAGAATATCAAAGCGGAAAAAATCCAAAA

>3 ERCC-00002 545 1061

TCCAGATTACTTCCATTTCCGCCCAAGCTGCTCACAGTATACGGGCGTCGGCATCCAGACCGTCGGCTGATCGTGGTTTTACTAGGCTAGACTAGCGTACGAGCACTATGGTCAGTAATTCCTGGAGGAATAGGTACCAAGAAAAAAACGAACCTTTGGGTTCCAGAGCTGTACGGTCGCACTGAACTCGGATAGGTCTCAGAAAAACGAAATATAGGCTTACGGTAGGTCCGAATGGCACAAAGCTTGTTCCGTTAGCTGGCATAAGATTCCATGCCTAGATGTGATACACGTTTCTGGAAACTGCCTCGTCATGCGACTGTTCCCCGGGGTCAGGGCCGCTGGTATTTGCTGTAAAGAGGGGCGTTGAGTCCGTCCGACTTCACTGCCCCCTTTCAGCCTTTTGGGTCCTGTATCCCAATTCTCAGAGGTCCCGCCGTACGCTGAGGACCACCTGAAACGGGCATCGTCGCTCTTCGTTGTTCGTCGACTTCTAGTGTGGAGACGAATTGCCAGAATTATTAACTGCGCAGTTAGGGCAGCGTCTGAGGAAGTTTGCTGCGGTTTCGCCTTGACCGCGGGAAGGAGACATAACGATAGCGACTCTGTCTCAGGGGATCTGCATATGTTTGCAGCATACTTTAGGTGGGCCTTGGCTTCCTTCCGCAGTCAAAACCGCGCAATTATCCCCGTCCTGATTTACTGGACTCGCAACGTGGGTCCATCAGTTGTCCGTATACCAAGACGTCTAAGGGCGGTGTACACCCTTTTGAGCAATGATTGCACAACCTGCGATCACCTTATACAGAATTATCAATCAAGCTCCCCGAGGAGCGGACTTGTAAGGACCGCCGCTTTCGCTCGGGTCTGCGGGTTATAGCTTTTCAGTCTCGACGGGCTAGCACACATCTGGTTGACTAGGCGCATAGTCGCCATTCACAGATTTGCTCGGCAATCAGTACTGGTAGGCGTTAGACCCCGTGACTCGTGGCTGAACGGCCGTACAACTCGACAGCCGGTGCTTGCGTTTTACCCTTAAAA

>4 ERCC-00096 564 1107

AGATAAAACGAATAGCTCGTAACCAAACATGCACAGCGGTCAAACAGTATGTCCCAAGGGGACTTAAGCGCGGTGGCCTCCCCTATCCCCTACGAGGCTACCCGGATCGATGACGCGAATTGGGGACATTCAAATGAGCATCCTAGTCACCGCGTTTAAAATGAACCTGCCGGCTGATCGTTTTTAGGATATTGTGAGTAATATAGATTGGCGCTAGTAGATCACAGAACAACCGCCGCATACGGCCGATTGTCGCAGCCCGGGTCGATTATAACAACGGTGCAATCTCAGCTAAACCGACGCAGTTTTGCTCCTTGGATTCTGAGCCCGGGCATCGCCCCTCGTTTATGAACTAGCCTATCGCAGACGGTATCAACAGGAACATCCTCGTGTTAGATATTGAGGCTGCTTCGTGTCGGCACGAAGTGTCTTCCGATGCAGTGTCCAGTCATGACCTCGATCCATCGCGTATAGGGACGCCCCCTGCTCGCGTTACTGCCAAGCGAGCGTGGTGTGGTGCCCCCGACCTACAACTTGCGCCAATTATCGAGCTGGTAGACGACCAGCGCTGACGAGCTGGCGCAATGACGACCTAATTGGCGCACAGTACTAGGCATCGTCATCCAATGCGACGAGTCCTACACTATCTTGGATATGATATGGCGCACTACACATGCTAGCCGCTGGGGAGATTAGCTCGAGTTGCCCCTTTGCCCGATCCCGGAAGATACGCTCTAAGCTCGGCAATCGCTCTTGCCGTGCGAGATGCTAGCAAAAAGGTGTACTTCTCAGCGGAGCAGAAAGATCATGTTTATTGGAAGCATCAACCTGCGCCGTCTTGTTAACTTGTCATATCGCGCACGTAGTAGCCTAGAGCGCCAGGGGCGGAAATTCGCCTGAAAAGTTTTGCCGGCGCACAAGCACGATCGGCTCCTAATAGGAGGTGAATTAGATAGGGAAAAGATCGGGATGCTACTAGTTTACTGCGTCACGCTGAGGGACTCATCCTGGGCTACAATCCTATTGCCGAGATAGTATTTCTTAGCTTCCTGAGGGAGGTCAATTTGAATGTGGTTATATGCGAAAA

>5 ERCC-00004 180 523

TCTTGCTTCAACAATAACGTCTCTTTCAGAAGGCATTGGTATCTTTTCCCCACTTCCAAGCATTTTTTCAACTAATCTTATGTTATTAACCATTTCCTTAAATTCTTCTGGGTCTGCTGACAAAGCATGATCAGGACCTTCCATATTTTTATCTAAGGTAAAGTGCTTCTCAATAACATCCGCTCCTAAGGCAACAGAAACTACTGGGGCGAGTATTCCCAATGTATGGTCAGAATATCCCACAGGGATATTGAATATACTTTTCAAGGTTTTAATAGCGTTTAAATTGACATCTTCATAAGGGGTTGGGTAAGATGAAATACAATGCAATAAAATAATATCCCTGCATCCATTATTTTCTAAAACTTTAACTGCTTCCCAAATTTCCCCAATATCAGACATTCCTGTAGATAAAATCACCGGCTTGCCTGTTTTTGCCACTTTTTCTAATAAGGGATAAAAGGTTAAATCACCAGAGGCAATTTTAAATCAGGCACATAAAA

>6 ERCC-00171 241 505

CTGGAGATTGTCTCGTACGGTTAAGAGCCTCCGCCCGTCTCTGGGACTATGGACGGGCACGCTCATATCAGGCTATATTTGGTCCGGGTTATTATCGTCGCGGTTACCGTAATACTTCAGATCAGTTAAGTAGGGCCATATGCCTCGGGAATAAGCTGACGGTGACAAGGTTTCCCCCTAATCGAGACGCTGCAATAACACAGGGGCATACAGTAACCAGGCAAGAGTTCAATCGCTTAGTTTCGTGGCGGGATTTGAGGAAAACTGCGACTGTTCTTTAACCAAACATCCGTGCGATTCGTGCCACTCGTAGACGGCATCTCACAGTCACTGAAGGCTATTAAAGAGTTAGCACCCACCATTGGATGAAGCCCAGGATAAGTGACCCCCCCGGACCTTGGAGTTTCATGCTAATCAAAGAAGAGCTAATCCGACGTAAAGTTGCGGCGTTGATTACGCAGGATTGCGACCAAAGAACGAGAAAA

>7 ERCC-00046 182 522

ATCTCCTCTAACTTTGGAGAGGTAGGAATGGGAGTATTTGCACTTGTGGTAACGGTATTTGCATTATTGATGGTTTTTACTATGTTGGGTATGCTGTTCGATTTCTTAAAGGACTGAATATTCGGTGGCAGTATGGGATTTCTAAAAATAATGTTAAGAATTTTTGCTGGTTTTTTGCGACGTTGGTGTTGTATTCTATAGCTCCATTATGGCCGTTATATGGAATCATTGGAGTGCCAGTAATTCTACCACGCCTTATATTTAAAGACAAAAAGAAGTGTCTAACAACAACATCCACACTACTACTCCTTGTCATATTTCTTCCTGAATTGCTGATTCTTATTGGATTTCTGATATTTCCTATTGTTATGGGCTATTACATCTCTAAGGAATTGGTGAAGTAAAATGGTGAAGCTTATGAATTTGTGGAGTGAGAGGATTAAAGATAGGGAAGTTGTTGAAGTTATTGGCTGTGAGAGAGTGCCATTGATGAAACGTAAAA

>8 ERCC-00113 424 840

ACACCGGCGCACGCCACAGGCGTCATACTTCCCAAGAAGCGGCCATAGCCCAGATGCGAGGTGGAAAAGTCACACTAGAGCGACACCAACATCGTTACGCTTACACACCGGACGCTTGGATCAGTGGGAAGTGCTCACGCGCGGAGCCCACTGGGCGAACAGCAACGTTATAACGGCCACTCAGTGGTTCGTCACGCGCAGCCCCGGGTTCGTCCCCTATAAGGGCCTAGTACCTTTCGAGCCCCGCGCGTACTAGGCAGATAAGAACCCTCCAGCTCGGGGCCTCAAACCGATATTCCATGTGGGCCAACTGCCATGTTGTGTCCAGTCGCTATCGGAGTAGCCGCGCTGGTGCCACACGACTACAACCCTCGTAATAGGGCTGCGTGCGTCCTAAATACACTCGCTGTTGAGATACTAAAATTATCTGTGGATTGCCGGCATTGAGCCCACGGTAAACCCCAAATACATAAGTGTATAATGTCTCGGACCCGTCGCAACGGTTGTTAATATGACAGGCCGCTAAAGACGTTCTACTCCGCCATATGAGATAATATCCTTATCTTGAGACGCATAGCAAATGTAGGAGAGAGAGGTTAATAAGGCCTAGCCTAAAGGTTCTTGCAGAGCAACATCATATACCCTGTAGAACCCGACTTTTGGGTTTAGGGGCCTGCCGTCAGCTACAATTCATGCTTGAAGGTTTCACTAGATCGTGTTATGGACGATGACTATAGTGTAACAGGTGCAGAGCTTAACTTTGGACACATGACACTCAGTTCTGTACCAACTAGGAAGAGCGCCGGGGTAGAAGAATAAA

>9 ERCC-00136 429 1033

TTTCGACGTTTTGAAGGAGGGTTTTAAGTAATGATCGAGATTGAAAAACCAAAAATCGAAACGGTTGAAATCAGCGACGATGCCGAATTTGGTAAGTTTGTCGTAGAGCCACTTGAGCGTGGATATGGTACAACTCTGGGTAACTCCTTACGTCGTATCCTCTTATCCTCACTCCCTGGTGCCGCTGTAACATCAATCCAGATAGATGGTGTACTGCACGAATTCTCGACAATTGAAGGCGTTGTGGAAGATGTTACAACGATTATCTTACACATTAAAAAGCTTGCATTGAAAATCTACTCTGATGAAGAGAAGACGCTAGAAATTGATGTACAGGGTGAAGGAACTGTAACGGCAGCTGATATTACACACGATAGTGATGTAGAGATCTTAAATCCTGATCTTCATATCGCGACTCTTGGTGAGAATGCGAGTTTCCGAGTTCGCCTTACTGCTCAAAGAGGACGTGGGTATACGCCTGCTGACGCAAACAAGAGAGGCGATCAGCCAATCGGCGTGATTCCGATCGATTCTATCTATACGCCAGTTTCCCGTGTATCTTATCAGGTAGAGAACACTCGTGTAGGCCAAGTTGCAAACTATGATAAACTTACACTTGATGTTTGGACTGATGGAAGCACTGGACCGAAAGAAGCAATTGCGCTTGGTTCAAAGATTTTAACTGAACACCTTAATATATTCGCTGGTTTAACTGACGAAGCTCAACATGCTGAAATCATGGTTGAAGAAGAAGAAGATCAAAAAGAGAAAGTTCTTGAAATGACAATTGAAGAATTGGATCTTTCTGTTCGTTCTTACAACTGCTTAAAGCGTGCGGGTATTAACACGGTTCAAGAGCTTGCGAACAAGACGGAAGAAGATATGATGAAAGTTCGAAATCTAGGACGCAAATCACTTGAAGAAGTGAAAGCGAGACTAGAAGAACTTGGACTCGGACTTCGCAAAGACGATTGACTAGTTTCCCTTGTGAACTAGGATTTTCCCGGGTACAA

>10 ERCC-00009 465 984

CAATGATAGGCTAGTCTCGCGCAGTACATGGTAGTTCAGCCAATAGATGCCTAGTACGCTGACGGCATTCAGAGTACGCTGATCGGCTTATGACGTATGTGACGCAGCTCTTAGCGCAATGTATGTGCTGTTATCGAAGCCTATGGCTGAGTATGTAACGCTATGGCGTGCTAGTCGTCTCATATACGTCTGATGACCTCGTATCATGTTATAGGGCTGCGAACTGTCGATGATGGTCACGACTCTGTCGATAGCTGTGTGACTCATTCAGAAGGTGTGCAGCCTATATGATACGCAGTCGCATCCTATCTTACGTGTCAGTACTATGTGTGAGTGCTCCGCCCTAGTGCTGATGTATGCCCCATAGTGCTCAGTGGAGTCTCTCTTAGCATAGTGTCCGCTCATACATTAGATGGACGGCTCATTAGTATCATCGTCGGCTGATATAGGTCGTGGCTCCCTGTATATCGAGGTGAGTCTATCTGGATCAACGTCGCACTATGATGTGCAAAGTGTCGTCCATGTATAGACAGTGCGCGTATCATATAGGATGCGGCGATCTCATACAGCGTTACGGTCGCTGCGTACTGTATAAGGATGCTCTGTGAACTGTCATCGGTCCGATCAATTAGTCTAGTGTGCGTTATTCAGATCGAGTGAGTACATGATTCGTCAGTGTGGATCAATTACAGTTAGGCCGCTGACACATTAGTAACGTCGGCAAGCACTTAGTCGTGTCGTAAGCCAGTGTGTCGTGTCTTAGACGACTGTGTGTGATTCTCGAGCGATTTATACATCCGTGACAGCGCTTATAGTGTGCTGACAGACTGGTTGGTTATCCAATGATCGACCTGGAGTCTAATATCTGACCACGCCTTGTAATCGTATGACACGCGCTTGACACGACTGAATCCAGCTTAAGAGCCCTGCAACGCGATATACAGGCGCTGCTACCGATATAAAA

>11 ERCC-00108 501 1022

CTCTGTGTCATGATCGTGAGTTGTCGCAGTGTCTGTACCAATACTCTGGTGGAGCTATATAAGCCGCTGTTGCGTAAATCAACGGCATGATCCCTATGACCGCGTCATGCTAACTGATACACGCTGCTCGAACAGTGATACGCACACTGATAACTATGCGCAGACGCTTGAAACGATGTGACATCGCTTCTAGAGTATGAGCCGCAATGCACGACTGATACTCGATATGAGCAGCAGTCGGCTATGATTTGCAATGCTTGCAGTATGTATCCTGATCGTGCGTGCGATGTCTGATAATACGCTCGCATGATATGTATTGCGCTCAGATGCTGGAGATATGCCATGCGTGCTGTCAGTATGCCATGTATGCTGATATGTCGCGATCTATGTGGTGACTATGAGATCCATGTGATGACGTTGCAGTCTCTGTGACCTTATCGACGCGCATGTGAGCCTATAGACAGCGATGTGAGCACTCTCATCTGCGGATCAGTCTATCCTCGCTGATGCTCAGTGATACACGCTGATGCACGTAGTGAGCATCCTGTGCTCGCATATACCGCTGCTGCACTGATATGAGCCAGTGCTGCTGCTCTCTACGGAGTGTGCTCGGCTATAACAGCGAGTGCTACGCCTAAACTGGCTGTCTAGCACTGTAGCTGGTGCATGTACTCGACTGCCGCTGCATCTACTATAAGACTCTGACATTAGCGTATAGGCTGATACATTAGCTCGGATGCTATCAGCTTGCGCCTATTATATGCCTGACGCGGGATCTATCAGAACGACTCGGTAGCTCATATACTGGATCACGGTGCCACAACATGCTACACGAGGTCTCAGACTCTATCCCGTGGACTCAACGTGCATCTGCTATGCTGAGCGCGTATCTGTGTACCTGTCCGATGCTCTGATCTACACTGCCGTGATCGTTATATGACGAGACTGTGCGCTCATAGCCGACACTGTGCTCGATAAGACCACGCTGTGCGGATATAAAAA

>12 ERCC-00003 334 1023

CAGCAGCGATTAAGGCAGAGGCGTTTGTATCTGCCATTATAAAGAAGTTTCCTCCAGCAACTCCTTTCTTAATTCCAAACTTAGCTTCAGTTATAAATTCCCCTCCCATGATTGGGATTTTATAAACTTTTCTTCCATATAATTCATCTTTCTTCTCATAACCGTCTCCGAAAAACTTCAACTTAAATCCAACCTTTAACTGCTCATCAGCCATGTCTCCCACAGCATCAAAAATAGCAGTTGTTGGACATGTTAAGACACACTGCCCCAATCTCTCTAACATTTGATGCTCTAACTCTGACTTTTTAGGGTGGCATATCTGTATTATAAATCCTGGTCTTCCATCTGGTGTTTTTGATGGAGGGACATATTTCTCAATTCCTGCTTCTGCTGGACACATTATAACTGAACAACCAAAACCTGTTGCCTCTGTAGCTGCAATCTTAGCCCACTTCTTTGTAGCTGCTGTTATTAAAACTCTTGAAACCCATATTGGGAATGCTTCTGCAAATGTATCTTCAATATATACTCCATTTATTTCCATAGTTTCCCTCCATTAAGATTTTAACAATTATAGTTTATCTTAGGGGCTATTAATATCTTATCATTTGGTTTTTAATATTCGATAAATCCATAAATAAAAATATATCAACAATAATTTTAAATAATCTAAGTATAGGTAATATAACAATTAAAAAGATTTAGAGGGATAGAATTGAACGGCATTAGGAGAATTGTTTTAGATATATTGAAGCCGCATGAGCCAAAAATAACAGATATGGCATTAAAATTAACATCATTATCAAACATTGATGGGGTTAATATTACAGTCTATGAAATAGATAAAGAGACTGAGAATGTTAAAGTTACAATTGAAGGGAATAATTTAGATTTTGATGAGATTCAGGAAATTATTGAAAGTTTGGGAGGGACTATTCACAGTATAGATGAGGTTGTTGCAGGTAAAAAGATTATTGAAGAGTTAGAACACCACAAGATAAAA

>13 ERCC-00145 462 1042

ACTGTCCTTTCATCCATAAGCGGAGAAAGAGGGAATGACATTGTTCTTACACGGCACAAGCAGACAAAATCAACATGGTCATTTAGAAATCGGAGGTGTGGATGCTCTCTATTTAGCGGAGAAATATGGTACACCTCTTTACGTATATGATGTGGCTTTAATACGTGAGCGTGCTAAAAGCTTTAAGCAGGCGTTTATTTCTGCAGGGCTGAAAGCACAGGTGGCATATGCGAGCAAAGCATTCTCATCAGTCGCAATGATTCAGCTCGCTGAGGAAGAGGGACTTTCTTTAGATGTCGTATCCGGAGGAGAGCTATATACGGCTGTTGCAGCAGGCTTTCCGGCAGAACGCATCCACTTTCATGGAAACAATAAGAGCAGGGAAGAACTGCGGATGGCGCTTGAGCACCGCATCGGCTGCATTGTGGTGGATAATTTCTATGAAATCGCGCTTCTTGAAGACCTATGTAAAGAAACGGGTCACTCCATCGATGTTCTTCTTCGGATCACGCCCGGAGTAGAAGCGCATACGCATGACTACATTACAACGGGCCAGGAAGATTCAAAGTTTGGTTTCGATCTTCATAACGGACAAACTGAACGGGCCATTGAACAAGTATTACAATCGGAACACATTCAGCTGCTGGGTGTCCATTGCCATATCGGCTCGCAAATCTTTGATACGGCCGGTTTTGTGTTAGCAGCGGAAAAAATCTTCAAAAAACTAGACGAATGGAGAGATTCATATTCATTTGTATCCAAGGTGCTGAATCTTGGAGGAGGTTTCGGCATTCGTTATACGGAAGATGATGAACCGCTTCATGCCACTGAATACGTTGAAAAAATTATCGAAGCTGTGAAAGAAAATGCTTCCCGTTACGGTTTTGACATTCCGGAAATTTGGATCGAACCGGGCCGTTCTCTCGTGGGAGACGCAGGCACAACTCTTTATACGGTTGGCTCTCAAAAAGAAGTGGATAAGCTGTACAATCGTTTCATCATTCGGCGTGCGAATTAAAAAA

>14 ERCC-00111 468 994

GCCCTAAGTTCCGGTCCGCAATTTCGTTTCGTTGGGACGCTTGAAGCGCAAGTAGAAAACGAGATAGGGTGTCCCATCTAAACCGCCGTGCCAATAGCTTTAAAGGCCAGGAAACATTTAATATCCCTAACAAGCACGGTCCACCAACGGCATTACGACTTTACGAGTTCGCAGAACAAAGACTTTGCAACTGCAGGGGAGCCCTTCGATGCATCCGAATGAGGGCGAGCGTCGCAGATTAACTTCGAGCAGTTAACCAGGCAAGGATATTTCCGGCATCAAGCGTTTCTCCCTACTAATATCCTTCCTTATGTCCCGCATTAAACAGCACTCAGCATCTCAAACAATCACAAAACAACCCACCTCATCGTTGACCAGATGATAACGTGGGACATTATCTTTGGGCAATACCACCAAAATCGTTCTTTATGGGGTATCGCTCTTAAGCAGCGCACTTCTGCATAACTATGCCATACAGTTAGGTGCTACCATGAACATCCCGAGCTGCGTTGCATGTATCGGGTATGCCCAAGACCATACCTCCAATGCTGCAGGGGTTAAATTCTCCGTTCTCGTCTAATCTAAGAGAATTGTATAGCTTGCAGGCTAACATCCTGGGTCCAACCCCACATTGTAACTTCGCTGATTCCCACCACTAATTTCTAGGGTTAGTGAAGGTTCGATACATCGCGCATTGGGGGTCTCGGTCAAGAGGAGCGCGTAAGTAAAGACGCCTATCTTCCAGTTTGATCGGGAAACTACCCGAGGGGGATGGTGCTCAATTGGGCCTCGTCTGAACACAAGAGAGATCGCACGCGGAAACCGTTAACGCAAGAATATACAGCATATGGCATGTAAGCGAAATAACATCCTCCCACTGGGTGCACAGCGAATTAGTATCATAGTAGAATTAAGCGAGATCCATGACGTCACCGCTACTCAATCATCATCCACGTCTCGCGTCATACCCAAAA

>15 ERCC-00042 401 1023

ACCAACTCTGGCCTTTGTGTTGCTATTCCCCAAGCACACAATCCAGTATAACATCTTCCACAAACTCTACAGCCAAGAGCAACCATTGCAGCAGTTCCAATATAGACAGCATCTGCTCCTAAAGCTATAGCCTTAAATACATCTGCTGAACATCTGATTCCTCCACTTGCTATGATGCTAATTTCGTTTCTCAAACCTTCCTCTCTCAATCTTTGATCTACTGCGGCAATAGCCATTTCTATTGGGATTCCAACATGGTCTCTGAATACCTTTGGTGCTGCCCCTGTCCCTCCTTTATATCCATCTATAACAACTGCGTCAGCATCACTTGTTGCTATTCCAACAGCAATAGCTGGAGCATTATGGACAGCTGCAATTTTAACAAACACTGGCTTTTTCCATCTTGTTGCTTCTTTCAAACTTCTAACTAATTGAGCTAAATCCTCAATTGAGTAAATGTCATGGTGAGGAGCTGGTGAGATAGCATCACTTCCCTCAGGAATCATTCTTGTTGCTGAAATTTCTGCTGTAACCTTCTCTCCAGGTAAGTGCCCTCCAATTCCAGGCTTAGCTCCCTGCCCTATTTTAATCTCTATTGCAGAACCTTTCATAAGATACTCTTCATTAACTCCAAATCTTCCACTTGCAACTTGGGTAATTATGTGGTCTGCATAAGGGTAGAGAGCTTTTGGCAATCCTCCTTCACCAGTTCCCATGAATGTTCCACATTCTTTAACTGCCTTAGCAAATGATAGGTGAGCGTTTAAAGACAAAGCTCCATAAGACATATGGGCAATCATTATTGGGGTATCTAACTTTAAGTTTGGAGCTATTTTTGTTTTTAACTTAGCTTTTTTAATCTTCTTGCCATCAATCTCTTCTTCAACAAATTCAAACTCTAACTGCTTTGGTTTTTTACCAATGTAAGTTCTTAATTCCATTGGCTCTCTCAATGGGTCGATGGATGGGTTTGTAACTTGCATGCATCTAAAACAATCTAAAA

>16 ERCC-00043 335 1023

AATACCTTTACAAATGCTTTAACAAGAGGAAATTGTGTTTTTGCCAATTTAAGACCTAATTTAATAGTTAAACCATTAACCTTAGTTGTTCCAAGGCATAATATAGAGAGTGAGATACAGGATGAGCTATTTCAGGGAGTTATTCAGTATGCAGTTGCCAAGGCAGTTGCTGATTTAGATTTAGATGAAGATTTAAAGGTTGTTGTCTCTGTTAATGTCCCAGAGGTTCCAATAACCAATTTAAATAAAAGAAAACTCTTCCAATACTTCTATGCCTCAGCAAAGTTAGCTATAAACAGAGCTTTAAATGAATATCCTTCAAAAGAGAAGGTAAAGAAAGAGAAATATAGAGCTTTGCATCCATTAGTTGGATTTAGGGATGTTAGATTGGAGTATCCTCCATATCTACAAATTGCTTTGGATGTCCCAACTATGGAGAATTTGGAATTTTTGTTACAAACAATTCCAAATAGCGACCACATCATCTTAGAGGCTGGAACACCACTAATTAAAAAGTTTGGTTTAGAGGTTATTGAAATAATGAGAGAATATTTTGATGGCTTTATTGTTGCTGATTTAAAAACCTTAGACACTGGAAGGGTTGAGGTAAGATTGGCATTTGAAGCAACAGCTAATGCAGTGGCAATAAGTGGAGTAGCACCAAAATCAACAATAATTAAAGCTATCCACGAATGTCAAAAATGTGGTTTAATCAGCTATTTGGATATGATGAACGTCTCTGAACCTCAAAAATTATATGATTCATTAAAATTAAAGCCAGATGTTGTTATCTTGCATAGAGGGATTGATGAGGAGACATTTGGAATTAAAAAGGAATGGAAATTTAAGGAAAACTGCTTATTAGCAATTGCTGGAGGAGTTGGTGTGGAGAATGTTGAAGAGCTTTTAAAAGAATATCAAATATTAATCGTTGGTAGAGCAATTACAAAATCAAAAGACCCAGGAAGAGTAATTAGGATTTTATAAACAAGATGGGTTAAAA

>17 ERCC-00116 998 1991

AAGCGTTCACAGCTCGGCAATACCTGTGACGAGCTGCTCGCAAGATTTACGCAGTGTGGCTATACTTGACAGTGATGGCGCTTACTTCAGATGTATGGGTGATACTTCGCTATATGGGTGGTCACTTCTCTATGGCGCGTGACAATGTACTATGGAGCGGTCAATGTCAGTACGGATCGCGTCGATCTAGGTGACTACGCACGCCTCTGGAGTAAATCGAGTGCTCCGTGCGAAATACGCGGTCATCGTGCGAATAACCGAGTCATCGTGAGTAGTATGAACGTGTCGTGTTATGCAGCGGTATGTCGTGCTATAATGGCGTCTGTCGTGCTCATAAGGTTCCTCTGATGTGCTAGACGTGTCCATCGAGCTGCATAGCTATACTTCGAGTCACTTGGGATACTTCGATAGCGTTGTGAATAGTGTCGTAGGCTCCCGGGCACGTTGTTAAACTGTTGCCGCCAATTCAAGATTAGTCCAGCTCGTACTATCGAATACACCATCGTCGTATCGAATAATCGCACCTCGTAGGAGTCGGTTGCCACTCGTTGATAGTCAACCAGGCTCGTTAGATAGTAGCCCAGATCCTACGAGATGAGCTACGTAACTACAGTGATAGCATATAGGGTACGCTAGAATGCCAGGTCGTAGTCGAATTAGTCAGGTTGGATGTCTACTAGTTGACTTGGAGTATGCCATGAAGACTCGTCCCTCGATATCAATACTCGTCCGCAGGTGAACACTGTAGTCGGTGCTAGTGCCCACTTCTCGGTATGTGTCCTCAATTATCGAGTAGGATTCTAATCAATCGTCGCGGCTCACTAATTGTCTGCGGTGGCTACTAATGGTTACGGTGCCTGACTAATCGTGTAGGTGTCTAATACATCGTGATACGGGCGATATAATGCTCGATACGGCAAATATAGCTCCGTCCGGTGGATCCAGATCGCAGGGTATCGCATCGACAGACCTGGTATCGTCGTGACGAACGTGCTACTCGCTTATCGGGCCTGCTACATCAGTGGCGATGTTCGTAACCCTTAGCCGATCTTCTTACTTACGAGGCTACTATTCGATCAAACTCGCCTATCTGGTAATAACTGCGGTGATCTGGTAGCCACTACGTGCGCCTGGTAGCAAATACGGCGAGCTGGTATCACTATCGGCTCAGTGGTCCGACATAGTGCCCAGTGGTTCGCATAACTGCCGCTGGGTCCAATATAACACGCAGTCGTCAATCATACGAGCCGATGGTCGGCAATAGCGCCTGTGGTGACACTATGCCACCTCTGGTCTAATATAGCGCCCTGTGGTCGTATAATCGAGCGCGTAATCGTATATCCGACTGTAGGTGCGTAACTCGCGACTAGGTGGCTCTAATCTGCGTTGGTTGTCGCTCACAGTGTCTGGTGTTCGATACCCGGATCGGGTTCCGTAATCTTGGCATCGAGGTTTCGTACATGTCACGCGGTCTCGTTCATTCTCGGTGGTGCTCAGTACATCCAGTGGTGAGTCGCTACATCACACGGTGATCCGGCTAAACCTCTGGGCATCCGTATTAAGCGACATTCCTACGACTTATCAGCACGTCCTACGGTATAACAAGGCGTGCTACGGTCTAACGACGCTGGTAGCAGTCTATCAGATCGCTAGTACGAGTTAGAGATGCTTAGTACGCCTTCGAATCTATGATGCTCGTGCTCACGCGATGCACTCGGATTATGGCACATGCACTCGCGTAATGACGCTGCATCGCTCAGTATGATCCATGAGCGCCGTGAATGACGCATGAGCCTCGTATCGAGTGCATGAGCTGTCTTTCACATGATACATCGCTCTAAATCATCATGCGACAGTCTCGACAGCAGCTCAGCATCTATGCATCATGTGCCTCACTAGGACATCATGCTCGACTCTGAGACACTGATCGAGCATTAAGACTCTAGAGCGGCCGCCGACTAGTGAGCTCAA

>18 ERCC-00060 163 523

ATCGACAATAAACAAAGAAATAGAACTTTTAACACCAGAAGGAAAAATATTTTTGAAGAAAATTGGTTATGTTGATTACTATGAGGCAGGTAGTTTAAAATTAGCTGAAGAAACAGCAAAAAGAGATGAGGACGTTATTATATTAAAAAATCATGGAGTAGTTTGTTTAGGTAAAGATTTGATAGATGCATATATAAAAGTGGAGGTTTTAGAAGAACAAGCTAAACTTACACTTTTAAACCTTCTTGTAAAGAAATAGTGGGGATTTTTAAATTTATTTAACTCTTTCTAATTCACTAACAATACTCCAAATTTGTGTTCTTGTGTGAAGTGGCATGTTTGGGTCGTTGCTAATCTCATCTAAGATGTGGATTGCTGTTGCACTTCTAACGATTGGCTCCTCCCCCTCTTTTAAAACAGCTTCTTTAGCCTTTTCAGCAGCAGCTCTAATGTTTCTTGGAACGGTTGTATCGTTAATTATCTCATCCAAATCAAAGCAAAAA

>19 ERCC-00076 319 642

CTCCTCATGTGAGCGGATCACTATCTACAGCTGGTAATACTCTCAGAATTTCTAACTACCAGTCGTAAAGTAGGCGAGCGTTGGTTGGTTCCTCTTGAAGCGAGAGAACCAAGGTGCTGGCTATTGTCCGCCCCGTCTACGGCTAACGCGGGGTTCTGCCATCCCGGTTTAGCGCAGAATAGTATTAGTCGGTCAGTGGACCCCCTCCTGAATATCTATACTTAGCGGCGGATTACCCGGCCCTCTTCATTGTCGAAAGATTGGGAACCGCCTCTGGCTGTCACGCCCCCAGGCCCTAGGTGAGGTACTATGTGAGGTATTTTTTCAATGTTAGATCATCTATGTAGTGGATCTGAGAGATCACGTGGACCAAAGCTGATTGATTACGGGACTGGCCGTAAGTGCTGCCCGCGAGTAGATCGTCTAGATCCGGCTAAAATTCCCTGCGGTGCCTTAGCCACCACTCCTACGACGGGCCGCATCTTGGTTATTCTCGCTAGACACGGTTCGGGAAGTGGAGCATCGTTAGCTGCCAAGATGTGTATGTTGTCGCCACTCTCGCACCGTTTTTATAGTCGTTCTATTAGAGATGGTTGCACATGGGCGTTTCTCATCGGTAAAA

>20 ERCC-00022 355 751

CCCGGGCCAATTGCCTCTATAACTAGAGCTGAGCCCACCATTAAAGCGATTTTTTCGCACTTAGCCGTAATAAATATAATGATCCCGCGGTGTAGTAATTCTACGGAATGCACGGAATGTCATAAGCAGAAGGACGTGATGTGCAACCTACTCCCCTTTCCCAAGTAAATGTACGGGAATTATCGTTTCGTTACCGACAACCATGGGGCCACGTGGCCAGTTTGCCCCTATTAGGTGGATAGGCACTGAGTACAGAATATATAAAGCGTGACGGATGAAAACGCACCCATTGTCACCGATTGTGACTAGTTGACCCTATCACCCCTACTGTGTTCAGACGTCGTTCTACTAAAGGCCCGTGCCGCCGGAAGCTCATTTAAAAAGAACTCGTAAGTAAGCCGGCGACATATCTAGCAAAACATAGTCCCCCTTCTGCTCAGAGGTTATCCATAAGTGACTTACCAGATGGAGTGCCAAGGTACAGACCTCCCTCCCAACTGGTTCCTGCAGGACGTTGCTATATCACTTCTGGCCGTCCTTATGGGTTACCCCTCGCGAGTGCCATCGCATCGACTGACACACCTGCATTCTATTTTATGCTCTACTGACGGCGACGAGTTTTTTGTAGCGTCGATCGCGGAGTTAAGGTCATTGGGGAATAGAACCATAGCGCTTGGGTTTGTGACTTTCTCCCTAGATACGCGTTTGCTGACTGCGCTACATGGATAAAA

>21 ERCC-00092 564 1124

AGATGTATATATGATGTCCTTGGACGGGGTGGCGCAGTATTACTGCAAGAGAGCGGACAGATTAGTGTGTTGGAGCCGACACATCAAAGGTTCGTCCGGGGACCGATCTGCAGCCTACGGGACATTTATCCGTAAAAGCATGGCGCTGTTTCGTACTTATCGGAGGCCAGGTATCGTCGCGGCGAGTCTCCCCGACGACGGAGATGGGCGTTACTATCTGGGCCGTCTCGTACTCTGTTACTTGGCACAGATGCGAGCCCTCGTAATGTGCATCAGCTAAGGGCGATATTATAATGCGACGTTTGTACGGATTCGTTACTAACGTGTTGGACGCTAGTGGAATATGTGTCGTTGGTTAGCCTACCCATGGCTTTCGCGGCGACACATGCTTAGACTCTTTCAAAACTTCGGTGAAGTTCACTCAAGCCGCGGAGCGCCGTCGTAATTCACTAGGGATGGCGGTACCCGTGCCCGTCCGATTCGTAGCAACCTGCATCACGATTTTGTCTTCGGGCGACTTATCAGATACGGTAATGTAAATACCTGGCATTTGGGCACTTCTTGCGTTTAAGCGGGAAAGATCGCGAGGGCCCGCTATTTGCGATACTTCCCATGTCGGTGCCGTCGCCTCTATGTACTCGGAGACGTTAATGCAGAGGCTAAGGACAATTTACCATGACTCGGTAATCCGTTCGTCAAGCAGGTAGCTCGAGTCTCCCCACGGACACGTAGTGGGTTTGTAACGATCGATACCGAGTCTTTTTGTCTAGTAGAACCAACCAACCATTAAGGAGTTCACTAGCACATCTTTGCGACCCGATCGTCCGTGTGTCGCGTAATACTTTTGTTATGACGAGACATACGCTCAAGCCCTGGGTAGCTAGTCGCGGAGGCACGTTACCGCGCACAACCCCTATTCGTTTACATGTACATCGCATCTGAGGTAGTACACTTCCGGCGTACGTGAGTATTTGCGCGTAATAAGCGCGTGTTTAGCTGATCCCCTCTCGTATCGAGGTTAAGGCAGATTAGTGCCCAGTAATTGCGTTTTTTTGTCGTTGTCGCAGAACGCGATTTGCTCCGAAAGCTTTAAGCCGTGGAAAA

>22 ERCC-00095 194 521

TTGATGATGATACTTAACCAAACCTACCACTAATGTAGTCATCTGTCTCCTTCTTCTGTGGATTTAGGAATATCTGCTCTGTCTCTCCAAACTCAATTAATTTCCCCATTAAGAAAAAGGCAGTGTAATCAGAAACCCTACTTGCCTGCTGCATGTTGTGGGTAACAACAACAATCGTATAATCTTTAGCTAACTCAACCATTAACTCCTCTATCTTTAATGTGGAGATAGGGTCTAAGGCAGATGTTGGTTCATCCGTCAATAAAACCTCTGGCTTAACCGCTATCGCTCTCGCTATACATAACCTCTGCTGTTGTCCTCCAGAGAGAGATAGAGCGTTTTTATGCAGTTCATCTTTAACCTCATCCCACAAAGCCGCTTTCTTTAAAGCCCACTCAACAATCTTATCCAATTCTTTTTTATCCTTAATTCCATGAATTCTTGGGCCAAATGCAACATTATCATAGATGCTCATAGCAAAAGGATTTGGTTTTGAAATAA

>23 ERCC-00131 366 771

CGCCGGATAAAGGCTTCTTCGGTACTATACAAGTTGTCTACGCACATTTGCCTTGGGTATTAAACTCTAGTAGGAGCGAAACTTGACATATCAAACCTCTCTGGAGAGGCATCTCCCAGACATTACGGATGATCAGGTCTCAGCGAATCCTAGCTGTGCGGATAATTTGTGGCCCGCACAACCGCAAGACACCGCCGTGTCTATTCCACAAACTTCGACATATCAATAACGCAGTAACAACGTTAAGTAGGGGGACAGGCAGTGCGTCCTAAGGGGCTTGAGGTCGCCCGATTCGCGATCTAATCTTAACAGACGAGGTGGCGTAAGATGTACCTCGTTCAATCTAAGTATGAATTTGTCCGCTGGGTAATGGTGGAATTAAAGAACATGCGTAAAACGACCTCGTTGTAATTTGGGTCGGCCCGTCTCGGCAAATTTGCCCTTGCAGTGAAGCTCGGATTGGATGTCTCGTAAGGAATACTTATTGCGGGTGGTACGCAAATTCGCAACCATGTGAAGTAATGTGAGCGTACTTCACCTGCTCCGGTACAAGCCGCCATGTGCTCATGTTGGGAACCTCCTGCGTAACAACCGGACGCTTGCGAAAGTCATTCCCCTATCGGTACACTATTGGAAAACATGACAAACGTATAATGCGTGATGACCGCAACAGGTGTAAGTGCACCAACTAGTATTGGCTCCTGTCCACATGGTCGGGTTTTCCGCCCCCAAACATGCAAACATCCGAAAA

>24 ERCC-00035 580 1130

GGGAGAGGTATCGTAAAGGTGAATCCTGTGGCTGTAGGAAGCAGTTCATATGACCAACGGGCGCCCTAGGGTATACTCCAGAATCGAATGTTACCAGAAAATAGGGGAACTGGTCGCTCCTAGGTAAGGTTGCCTGTCCCCGCACTGTACGAAGGTCCGGTCAACGGAAGCAGGCACACGCGCTATCTGAGTCCAAATACTTGATCCGGCCGGAAAATTAGAGGTGACGGCTTCCCACTACTGATCTCACTGACGTGCAATCATCCTGTGTCCGGAGCAATGACAGTGTCCAGTCTGTTATGGACGAGCTGGACGATTCACAGATAAGGACAGTCGCCCGGATACCGCAAACTAAATCTTGCCTGCCTCCGAGGGTGTGTGAAGGATAACTGTCGCCCTTAAGTAAAGATCTACCATTCGCCGGGGCAACGCGGCTCTCTGATGCTACGACCAAAGTGGTTGACATTACGCCAGTAATGGACTAAGGTTGAATTTGAGCGGATGGGCTCAACTGCGTCGTAACCGGTAGATACAGGGCATACGAGCCTCCCTATTTAACGGCATCATCCCGCGTAGTGCTGGTCAACCGACTGTCTATTGAAGTCAGGTTTCGGCTACATCAGCTGGAACGTTCCCGTAGCTACTATAATCAGGCAATCTGTCCCGAAGAGCCCACACCCTGACTTGGCCGTAAGGGAGCCCTAAATCAGTTAAATACGGGGAAGGAGTCTCGCCCTCCGGGTGATCCATCTTATTTACCAAAGAACATGTCCACGCCTGTGGGCTCTGAAGATGGGCGACACATGTCTACCCTGGCATAACGCCCAGCTTGATAAGAGTGAAACGACCCATCTGACACAGGCCGGCATAGAAGACCAGCGCAATGCGAGGGCCACACATAGGAGTGTGAAAATCTACATCAACATACTTCGGCCGTGGCTGAACGAGGGAGTGCTTGTACAATCCCGTTGTTTGGTGTGGCAGATTCCGGGAAATGAGCAAACGCTTCTCCCATGAACACACTCACAGCCATAAGTCCTACCTTGCTAACATCGCCCCGAATGCAGAGCCACCTACTGGGGTTGTCTGAGAACACTCCATGGGTCAAAA

>25 ERCC-00112 535 1136

AGATGCCTGGATAGGTTCAAGTTACGTTTACACCGTTCCCACCCCACTAACCACGACCTTTCTCGCCTTTGTTTTACCAACGCCGGCTAACAGGTCGGGCCCAACTAATATTGTTAAGGCAGGCGTTCTGTACTTACTCGATCGTTCATAGCCCCACGACATAGTTTTTAGTCATTACGTAGCCGTTATAGAAACTCACCTGTTGTAGAAGGTTACAACCAGATCGTCTTACTTATGGCATGCTGGCGTAGACAGGTGCCAATTTGCGGCCAGTACTCGGATGTTCGTGGACTGATCTGGTCCTCTGTTCCCCTGGTGAAAACGTCGCTTCACTCCGGTAAGGGCGAATGTAATGACCGTATTCTAAGGTACGGATCTACACTATGTGCGGGCGAATAGACTCGGGCCCGTAGCGGGGAGCAAATACTTGATCGCAATCTCTGACGTCAACTGATGTCGCGGAGACTGCTGCGCACCCAATAGGTACCACTAACGTCCGTGTGATTTTAACCGGTAGGAATAAACACCGTCTTATACAGCTACGTTTTTCGATTTACTACCGAGCCGTTTGCGAGAACATATGGATTGGGAAATAGGCTCGAGTCCACATACAGCCTACGGTAAGTTGCTAGGCTCGCAATTCTATCCGGGTTTGTGAGTCTTAGTTGTGCTCCGGCTTTTGAATGAGCATACTCATGAAAGCGCTGCTACTATGATAAGAGTACACGTACAGGTCTCGCCCGATTGGATTATGGCGAGCTGCCGCATTGACGGACATACCTTTGAACGTAATCGCGCACGAGTGCGGATTAAGATTCTCCGCTTCAATCATGCAATGTGGTACAGCTGACTATCATTGACACCAAGCCGCTTGCAGGTATCGCCTGTCGGATAAGTTAAGAGTGAGACGAAGAGTATTCATCGAGCGCCAGGTAAGATAGTGCCACTCTAAGCATCGGTATCTAGCTTTAGTAACCTTCTCGATGGGGAATACACCTCTGCTTAACGGTCTTAATAATTGACGCGTCTTGGACGTAGTACTCTGCAGTGCCTAAACTCATAGTAAACGATCTGGTAGGTCCTTTTACACACGGTTTTTATCGCTTAGTGCTAAAA

>26 ERCC-00044 578 1156

AGATAGTGACTAGGGTAAATGCCAAGCCGTCTTATAAAAGCGGTAGCGAGAGATCTTAGAACTCCCGGCGAACGCCCCAACTTATGCGATGTCCAGCCCCAAACGGTTGATTAGATCGGTAGCTCCCGAACTAAGAGACCCTAAACGCTATCCGTGGACTGTGTAGGTAGGCCGTCGTCCTAAGTGGCCTCGTGAACTAACGCCTGCTGCTATGTCCCAATGAAGGGTACATGCCCTCTTCCCGTACTACGCCGATACAAAGTCCCGAACGAGCTTACGAAATATAGCGCTTAACCATGGGGCCACCATAGGTGCCTGGTACAGACGTGTCAGACGAGCCCTCGGCTAGCTCATAATTGCGGCCGTTCATATAAACCTCAGCGTCTGGTCATGTCTGTACCACAACGCTCCCGGTTCTTCGACGCTCAGTTCGGTACCCAAGATAGCGGACTCTCAAAGAACGATTCAGTAGTCGCCGTACCCATGTAGGCCTTAAGAGGTCTAATTAGCTCTAGGAAACACAACCCCGGGATTTGGGATACGCCACCAACACGAATCCAGCCGCATCGCACGCGTCGAAGTCCTCCGTGCTTGTGTGAGTCGACACATAGGATCGGGCACGGAGAGATCGGCGGTTGAAGTATGGAGCGTAGGTCTCGATCTCGTACAAAACGACTATGACCATTGGGCGTTGTACTCATTATCGTTACGATTTTACGGTGGCAAACGAAGCTACCAAGTAGATGCCGACGTCGAGTGGATCCACGCAGTCCGAGACAGCGCACACTCGCTGTAGAGGGTTTCAATCCGACTAAGACGGCGGGATCTACTACTTCTGCGAGTTTAGATTAACTGTATCTCCCGAATGCGCGTTCTAAAACCCTGACGTGATGGGACGGGTGAAGCACCGGGTTTTCGCCGGTTAGGTAAGGTAAAAATATGACCTTACCCTACGTTGATTGTGGTCATTACAGCAAGAGGTGGGTTATAACGCAATCTATGTTCGCATGCTATGTTATTTGACCCCTGCGGTAAGATTATTCACTCGTCATGGATAACCGGATAATCCCGTATAACCTGTGGGTATTATAGGCGACAATTCGAAAAAGGGCGAATTCAGGCCTGAATTCTGCAGA

>27 ERCC-00051 94 274

TTACAATAGATGCGTTTAGAGTAGCTGGGGGAATTTTGCTCTTTAAAATAGCTTGGGACATGCTTCACGCAGAAATTCCAAAAACAAAGCACAAACCAGATGAAAGATTAGACCTTGAAGATATTGATAGTATAGTTTATGTCCCATTGGCTATTCCTTTAATCTCTGGCCCTGGAGCTATAACAACAACCATGATTTTGATTAGCAAAACCCAGAGTATCTTAGAGAAAGGGGTTGTTGTTCTCTCTATAAAA

>28 ERCC-00162 189 523

CTCCACAGATTCCCATGTGTCCAATTCTGAATATCTTTCCAGCTAAGTGCTTCTGCCCACCAGCAACAACTATGTTGTATTTGTTGCTTAATATACCTCTAAATTTGCTATCTTCAATGCCTTCTGGATATTTTGCTGATGTAACTGTTACTGACCTTGCCCTCTCCTTGGCAAACAACTCTATTCCCATTGCCTCCAAACCAGCCCTTGTTGCTTTTGCTAATCTCTCATGTCTTTTAACCCTATTCTCGATTCCTTCCTCTAAAACTAAATCTAATGCAACATTTAAGGCATAGGTTAAATTAACTGATGGTGTGTATGGGGTTTGTTTTTTCTCTTCATAGTATTTTTTATAAGCCAATAAATCTAAGTAGAAACCAACTTTGTCATCATTCTTCTTAATAACTTCCCATGCCTTTTCACTGACTGTTATTGCAGCCAATCCTGGTGGAGCTGCCAAACATTTTTGAGAACCAGTAACACAGATATCATGTGGAATAAAA

>29 ERCC-00071 311 642

CATGCGAGAGGCCTTGAGCCGTTGGCCGATCGCATACAGAGACTATTGCATCAAGGTAACACTTATTAATCCCCATCGTCATAATCGCTAGATTCTAGGTATTTCGGATCTTCCTAGTTATCAGAAACATGTTAATTGGGCTCGGGCGTCTGCGCCAGGCCTTCGTAGTCCATACCACGATCTGTATTTTGCACCTTTCGCTATGCTGAGGTTGTTGACATAAGGATTAACTGCTGTGGTGTGTCATACTCGGCTACCTCCTGGTTTGGCGTCAAACAACTTCCAAGATTATCTCATACTATAAAAAGACGACATGCACCGCCGTCCTTAAGTGCTTACGACAGAGGGTCGTTATCTCTCTCGGTCTCGGTGCGCCTCTTCACGGAGCAGCATGACCTGCTAACCTGCATCAGCCAATGTCCCGTCTCGAGCTGGCCTACGGATGCGTGAGCAAAGGTGCTAACTCTTTTCATAACCCGGAGATGAACTACCACGCTTCCTGCGTGGGTCCCGCGAAAACGAAGCAAGGAGATTCTTCGCGACCTGGCCCATTCACTACATAACAGAGTTAAGACTTAGATCAGCGAGCAGGTGTACGCCCCGGACCTTGGGCTACTTAAAA

>30 ERCC-00079 318 644

GATGTTGGAGTTAACGGAGACCCGCCATCGTTTACGAAACAGGTCGCAGATAAATGTGGAGCAGATTTACGTTGAGGGCCTTCTGGGGACCCAAAGGATGAACGGGGTGTTGTTTCAGAGCGAATAGAGCGATCACCAACGCGCACGTCTCTTTTTAAGATTGAGCGGCTAGGTGTTTCCTACAGTAACTCAATTAGCGTGAGCCAAAGGGCGGAGCCACGGGCCAGTAAGCTATTTAGGGTTTACATGGCTCGATACCGAAACGTGACCGGTACGACGTTCATTTCCTCTGCTTTGGAGTTATCAATTCGTGACCCCGATCATCCAGTCCAGAAGTCGCGGCCCGAAGATCAAAGACGCTACTGACTTGGACTGGTACGAGAGCCCGAGAGTTTAGTGTGCGCACCCCACGTATTTTTTCGCGTCGATCATGCTTAGATTTTAACAACCCGCGGGCCGAAGTTTGATAAGCGTGTTCTAGATTGGAACTTACCACTGTTAAATACACGACGCCCATCTCCAAATCGCACGCAACAGGGGGCTTCCACCAGATACTCCCCAGGACAGGTGGAGATACCAACCACGAAGGAGGTCTCGTGCTGAGCCTTCGTCGATATACCAAAA

>31 ERCC-00165 436 872

GATATGCGTTACGTGAGTCTGATAGCAGTTCACTACCTGGATATCTGATCCACTAGCTCGATCATGCTCACCCATAGTTTATCTGCATCACTCGTACTGAAATGCTCACATCGCAGGTAGAGCAGCATCGTAGAGCGTCAAGCTGCATCCTAGCGTCATGAGTCATAGTACCTCATGCTCACGTGATCTACCCTAGCTGACCGCTAATGACGGCAGTGCAACCTGAGATACCGACGGCATACTGTCGTCAACGTCAGGCAATGTGTCCGAACGGCGAGCTACGTCGCCTCACGGAGTAATCGCGTCCCTCTAGGTATAGTGCCGTCGGTTCAGGTCATATGTCGCGGGTTCTGCACATATCACGGACGTATCGCTATCAGACGGACGCTCTCGGACCTAAACCGTAGCTCTCGGCAAGATCGTCCTCGTCTCGAATATAGCGCCCTAGTGCTGCAAATGTCACCGCTATCTCGTAAGGGGTCCGTCTGTTGAGTTAGGCCTCCTCTCGTTGGATGTGAGCTCGGTTGCTTGGATGGTGCAGCTTACTTCGCGTACCTGCTGTTTGCATCAGTCCTCTGCATCTATAATCGCGTATCTCTCTCTAGTAGACCATATAGCCATCTAAGCGCTCGATATTCCACCTAAGTGGCGCCTATTGAACTAAGTGGCAGCCGAATGGACTATCGCTCCTCGATATGTACGGATAGGCCACGGCATGTACGAGCATAAGCCGAACTGCACGAGCATACCCGACACTGATCTGAGAGTCGCTTAAATCATCTGCGTGTCTTAGAGCTTATCGCCATGTCTGTCAACTGTACTGTCATCCTGTAACTGTAGCGTATGTGAAAA

>32 ERCC-00062 315 1023

ACTATTGGAGAGAAAGATATCAGCCAGACCTATTTAGTAGCTCCACAGGCGCTAATAAAAGAGGCAGTATCATTAATTGGAAAGAGTGCTGTTGAGGGGATGATTAGAAGAAGTTCAAATAAATTATTCAAATAATCATAATAAAGTAATACTTATACTGAATATTCGAAATTATTATTTTGGAATTTTACAATATAGGTGATATTATGGCATTAAAATTCACCATTGAAGAGTTATCAAATCAAAAAAGAGATACATTAGGAAGAAATATTGACGTAACTGTTTTTAGATTAATAAGATTTATGGATTTGGAAAGATATTTAGGAAGAGGGGCTCATGGAGTTATTTACGAATGTGGAAGAGAGCTTGGACTGGCATTAAATCCAAAAACTATTGAAGATGTAGTTAAGTTTTGTGAGGAATATAAAATTGGAAAGGTGGAGATAGTTAATAAAGAGCCATTGTAAATTAGGGTTTATGAATGTATCTCTTGTTCTGGACTTCCTGAGGTTGGAGAGACATTATGTTGGTTTGAAGGAGGCTTTATTGCTGGATGCTTAGAAAAAATATTAAACAAGAGAGTTAGAGTGAAAGAAACTCACTGTGCAGGTTTGGGGCATGATTTCTGTCAGTTTGAGGTAAAAGTCCTTTAATGATATTTTTTCATCTCATTTATTATATATTCTAAAGCTTCAAAACCCTCTTTTCTATGTCTTGCAAAAACGGCTATTGAAGCAATCCTCTCCCCAATACTTAAAAATCCAGTGTTGTGGTAAAATAAGATATCAATAACATCAAACTTATTTTTTGCCTCCTCAATAACTAACTTCAACTTTTCTAAGATGTCTTCATCTATCTTCATTCCTTTTGATGGAACTTTTTCTCCATCTTTTAAATCATACTCCCTAACAAATCCATTGAAAGTTACAATACATCCAAATTTCCCTTTGTATTTTTCAATACATTCATCCATCTTTTTGAAAACTCTTCATACTCGTTAAAA

>33 ERCC-00025 1000 1994

GGGGTCCATTATAGTGCAGGCGTGGTAAAGTAGCATTAGAACCCTACTACTTAGCCCGCGCAACTCGTCCTATTAAAAGTCGGAAGGATTAGGGAAGTTAACCTCCGTTAGGGCCTCATTGGCGCGCCTCCACGTATCTGTATTCCCGTGTCCCGACTGGCTAATAAGAGATATGGTGCAGCGTGAACGCGGACCGAACTCCGGTGTGCGCCGGTTCATGTTACTCGAAAAATAGACCCTACTTGCGCCATGTCATCTTTCACTACGGGGTAGTCTCTCGGCCAGGCTTTGAATCGCCTCATCCGCTTCTCAATGGTTCTGTCCTGACCTCTGGAGTTAATCTTCGTCTCATAGAGTAACCAGGCGACGCACCACGCCTAGTTGGTACCACCACTAGAAGCCCGGTTCGCTGGCTCAGTGCTAATATTCTGGAGGTCAGCCGACAGGTCACATGAGCCTACTCGTCTCGTCAAACACCCGCCTAGCCATGATTAACAAATACAGTGATTAAGTGTGTTTTGACCCTAGTGTTAGGTCGCTGGTACTTAGCAAGGGGAGTTGCATATGTGGTCTCTGTTTCGGGTTATATCTATCATTTACTTGACACCCTATTGGTTTATCACAGTCCTTCCACACGACCATACGCGTTGTAAGAATACCCTCTAGCGTTGGAGAGAATAAGTTCGATGTTCAATTCACAAAACCGAGCAAAGGCTTGTCGACAGCTTCTACTAGTATTCACTACCAAAACGCGTACCGACTTTAGGGCGGTAGAGAGAATTGCCATTGCCACGAGGTTCTCCAGAATACAGGGTCCAGGCGGCCCCCAGCATCGGAGTGCCTGAACTGGCGGGGATGCCCAAGATTGTAGAGGGCCTAACGAGTTTGATACGCCCGGGACTACGGGGCATTGTCTGCCGCGTCTGTTTCAGGTAAACGATCAACCGGAGACCAGGTTATTCCCATGTCGGTCCCCAGGCTTATACTGCGGAGATAGGCTGATGATGGTAGGTGCCTTGCTCTAGCAAAAACGCGCACAGCTTATGAGCATAGCGGCTGGGCGTAGGTCAGAGAGGGTATGTAAGATCTCTCCTCTATCGGTGTGCGTCTACTCTGCCCCCTTCGACACAAATGTACATCGCGGGAAACGGATACGCCTTACGCCCCAAGGTTTAGCGTATCAAAAAATGCAACGATTCCGATGTCGAACCTTGGATAGGAGCGACCGATTACGTGTTAATTGGCCCTGTACCTTTCTGGTCCGTTGATTATCACTCATCCCCAGGTTGTTATGGTCTACCGTGGAAGCCTCGTCAATGAATACTTTGAGCGCGATTGAAGTGCTCCGCTACATCGCGGCTATCATTAAAAATTCCCCTTTAACAAAGGTTGTGGACGTCAACGGCCCTGCAGTAGCGTTCCTTGGACCCGGTTCGAGTACCCAGAGCAGACTACGTATATATCCAAGTGGTTATGTCCGACGGCATTTTGCCAGGTTAGTATCTTCGGCAATGAGTTGCGTCTACGGCTAATCGAGAGCACTTGTCAGTACGCGCAATTACTACATGGAGTGTATTCGTTTTGAGCGCTGATACAGTTCTCCTATCAAGGTCGCAAAAGGTTGTCTAGAAAAGGGCCGTGCGCGCAGTCTACTGCTACCGGCGACTTTACGACGAAGTTAGGCATCAGACGACCACATGACGGAGAATTACGTACGCCACTTCAGACGTTCTGCCTGCGTGCTCTGCAATATAGGCGTAGCAACGAAGCTCGGTCGTGAACTGCTATAGGAATAGCCTGATACGAGCGCACAACAAGAACGAACCAGCGAAACTACCCAGATAACATCGCTCCCCGGCATTACTCTGACGGGGGTTGTCACTAGGCTGGTGGGTCCTTTCGCCTCTCATGTCAATTCCCTTTGTTGGGCTGCTTGGAAGTACTGATACTAGAGTAGCCTAGTAATACCTAAAA

>34 ERCC-00144 245 538

AGCTAGGATAAATTGACCCGTATGAACTGTTGCCGGCTCGGAAATGTTAAGGCTCTGCGCACGCACTTTATCATTCGCAGCCTGTTCTGTCAGCGGGTCAGCCTAGGTTACGGTGGAACCACTCGGTATCGTGCAGACAGGGATCGTAAGGCGATCCAGCCGGTATACCTTAGTCACATATACTATCGTAATATTGGCGGTTGCTGACAAGTAAATACGGCTAAACCGGTCGTTGACCAACCACTCTCGCGGGGGTCATAAATATCACTGAGCCCGGGAAGTACCCCGTGACAGACATACGAAAAGCGTGATAACGTATTCGTAGGTATTATTTCCGTTAGCTGGAGGTAAAGGGGTTCTGGTCCTAGCCGTGTTATGTCTATTTATGAGATGGTAAGCTCGTCACCAACTCGTCACGCGATCGAAATAGCTTGGACTAATGTCCGGCACATAATCAAGTCTACATCAATCATGAATGGTTTCTGATTTGCTACCATCAGATATCATGTGAGCAAAAA

>35 ERCC-00019 314 644

CTTTAATGGTTGTACATACTTGACGGATTGCAGTGAGTGTATTCCCGTCCCATCGTATGGGTAACCCATAGGGGCGTGTCATTCAGCACTTCGGATAGTGTTTGACCGCGTCGCTCCCATGTCGCCTTTCAGGAGAAATAGTACAGGCTGCTGGCTCATGTTTCCTTCTACGCTGCACTTGCGGGCATAGAGGTCGGTTGCGATCTATATTCGGAGATAACTATTCACCCAGCGCCACTCGAATATCCCCTCTTCTGAGCAAGAGGCCAATAAATGCTCAAAAACGAGCGATTGTCCAACGACATAAAGGGAGACTGTAAGGTCCTAGCGCTCTGTCTGTTAGTGAGAGCCCTAGGTAAACACGGTCGTTATCCCCTAGAGCGTGAAGCGGCTGTAGATATCTCTGAATCTTCGACCTTGGTGTAGATGGGGCTAGGTCAAAAAGCGGTAGCGATTAGCACTTGTATACACTCTCCCCCTACTAAGTATGTAAGGCCTGACCGGAGATTTGTCCATGCTCACCAGGACCGATAGTTGGGCCCCGGTAATCTTGCCGCCGTAGGGAGTACGAGCAGTGCACCGTTGAAACAAGCACAGGAGGTATGAAGCATCAGACCTGAAAAA

>36 ERCC-00078 497 993

GGGGAATTGGATTTGCAGAGCATATTAGCAAGTTAACGCCGATCACTCTCCGGCAGTTGCTCCATTAAATACGGGCCTTCGCAATCGCGTGTTGTTACACATAATGCCGGTATCCATCTACTATCGCCGCCAGGTGCAAGAAATGCATATCCCGGCCTACCCCCTTAGCAATCGACATTTGTCTTTGCTGGACGCGCATGATTGAGTTATACGGAACTTCGCAAAAGTATTCCCTTTGTGGTCCGTGCGCCATGCTCCTCTGGGCGTAGCTTACAAGGACTAGGCCGTAGCCTGTAATTAGGGAACCGAGCACCAGAGAATCAGGGTCACAGTGTGTGGTAGACGAAATGATCGTGGGCTGAGGGAATTAGGAGGCGCCGGTCCAATAGTCGATAACACCTGACGGAAGTACGGCTGCAGGATCTAATCATATAGTTCAGAATTGCACGCGCGTCTAAGAACGGACCAGTGTAGACACAATACAACTTAAGCGCAGATCTGAGAGAGGGGTGCACAATCCAGGCAAAGTCCGTTAGAAGGTCAACCTTGTGGGTGCAGCGCTTTGCTCCTAAAGATTCGCTCCTCGGGAGCGACGCGACTGCTAGGGACTGGTAAAACGTTGGGCGCTGTCCAAGGTACCCTCAGTCTGTATTTCATGCCCTCAAATATGCAATCCCGACCCGGAGCCGGCTTTAGATTTATAGCAGAGGGTCTCCGAGGACTGTAATGTCTTTTGCGAGACAGGAAAGGTTGGGCGGTGTACGTCACCTCTACGGTCGAGGCTTGGGTGTATCATGGAGTAATCTATATTCCTTACACGCTTGCTTGCTCCTACCATACAACCACGCAGTCATTACGCGTACATATACCAGCAATTCTTCTACGGACCGTTAGGAAGAAAATGAACGGAGTGGCTGGCCCATAATTTCGCACCCGAAGCTACTGGTCGGACCCATCAGCGCGTGTCGTAAAA

>37 ERCC-00084 500 994

GCGAATTGTCTGGGGCCTCGTTGTGACTATCCTATTACGGGGATCTCAGGTGTGGTATCCCTGGTTGAGACATTGGACTAGTGTAATTGACAGTCATCGTATGCGGGATACCTCAAGTTGTATTCGAGGGCTTATCGGTGATACTGTGTAATCCCTTCGGCGAAAGATGTATGCGTGAGCATTAAATTTACGTGTTTCCTATATCGGACGCCTAATCTTTACCCGAGAAAGATATCTCTGTAAGTTTTCAAGCAACCGCGTCCTAATGTATATAACTTGGATCATCAGATAGGCCCATTACACTCTTCGGTGCTGCCGATATCCGACGAGCGGGCTTTTCGACTTGATCAGCGCTGTGGGTAGAGCTTGGATAAGCGAGGTCAGTCAAGCGATTCGTTGCCTCCGGGTCCCACGTAGATCGTTTGCCTGCATTTTATAGGTAGTGGCCTGCGTTCGCACTCCGAGGCACTGGGAACGATCCTACCAACATAACGGTCGAACTCGGTGTCGGATGACCCGGACGGGACGCCCGCGCTCACACAGGTATCTGGCGTGTACACCCGAATCGGCGGGGCTCGGCGACACAAAATGCTTCTAACTCGCTGTGAATCTACTGCAGAACTATGGGTTTGCTAGCGCGCCGGTATCTACCAGCAAAACATAGGAGCGTGGCCGAAACTGGGTCACTGAGTGGAATATATCCGAGTGCAGCTGCCATTAGTGGGGCGGTATCGGGCGTGATTGAAGGTAGGCTAACATTAGTCATCTGGTAGGGCATATTTTACAAACGGTCTAGGCTGCGGTTCAGATAGGGACTGATATACTTGATGTGCCCCCGTTCTTCAGCGTGCAGCTATGCAGCGACAGACGTTTGGAGCCTTAATCAAGTACGCATCGGAGCATACGGTCACTGGGGCATTTGCAGTGAAATTAACCATCCCCGTTCCCGCTTCTCAGTTGCAGGGTGGGGAAAA

>38 ERCC-00053 319 1023

ACATCTGTATAAAAAACACTACAGGGTGGTAACATGGTTCTATATAAAATTAGAAGATCAAAAAACGATCCCTGTCCATCAATACCTTCAGCGGTAATTATAGGATATTCTGTTGGATTAAAATTAATTACTGGACATGGAGCTCAGAGCTTAAGCAATATGGCTGGTTCTTATGCTGGAAAGGAATTGGGAATTTACGCAATGAATAATGGTTATGAATTTAAGGATATTAAAGATATTGAAAGATTTCTTAACCAGTTAGATTTTGCAAAGATAGAGATGAATGAGGAAGAGGATGAAATTATAGTAAAGATATCAAAATGCAATCTCTGCCCAAAGAGAATTTGTGGCTATGAATTTGAAGGAACAGCATGCCCTTGGGGAGGATTGTTAATTGGATTTATAAGTGAAACTTTAAAGTATAATTTAGGCTACCAAATGAATTTAAAGCCAGCTGAAACATGTATTATTAAATTAAAGAAGAAATAAAACTTATCTAATATTAAAAAACTCCAAAATCTCATAAGGATGCTTTAATACAATAACATCCTCCAACTCCATCAATTTTCTTGTATTTTCAGCATCTATCTTTCTAACCCTCATCTTAATCTCTTTTCCCTCAATTATTGCATTATAAGGGCAAACTTTTTTACAATTTCCACATCCTAAGCATTTAGATAATAATATCTCAACAAAATTATCCCTCTTAACTATAGCTCCATTTGGACAGACGTTTATACATTTTAAACAGAGTTTGCATTTCTTTTTATCAATTGCATAAGGAAGTTTTGTTGTTACAATCCCAGCTTTATAATCAACTGGAACTATTAAAGATTTAACAAATCCTTTCCCTGCCTGAGCTATAGCATTTGTTACTAAGCTATCTGCAATGCCATTAACAACCTTAGCAACGGTATTTCCAGTAGCTGGTGAGCAAATTAAATAATCATACTTTCCTAAGCTCAATCTTCCAGTGATTGTGATGAGTAAGGATGTTCTAAAA

>39 ERCC-00054 102 274

GATAAAATTGGTTTTGCCTTTCAGCAATTCAACTTAATTCCTTTATTAACTGCCTTAGAAAATGTTGAACTTCCACTGATTTTTAAATATAGGGGAGCAATGAGCGGAGAAGAGAGGAGGAAGAGAGCTTTAGAATGCTTAAAGATGGCAGAGTTGGAGGAGAGATTTGCCAATCACAAACCAAATCAGTTGAGTGGAGGGCAACAACAGAGAGTTGCTATAGCGAGGGCTTTGGCAAACAACCCACCAAAAAA

>40 ERCC-00148 240 494

CATCTCCATTTGGTCACGTTTACAACCGGAGTAGACGGCCATAGCAGGAGGGGTGTGCGACAGGCAGGAAGCTCTCGCGGGGTCCAAGCATTGCTGCAATGGGCGTCCTTGCTCGATGTTGACCCGAAGCTCTAACTGCATTTCAAGTCCGAATCTCTAGATATCTCGAGAGCCAATGGTTAAGGAAGGGGCGTCAATTTTGCGCGGACACGCTGCAGGATGTAATGATTAAGCCGTAGTTGAATTTATGGAGCGGTGCCCCGGGAAAGGTATAAATCGGAGGCAGGGGTTTACGGCGTCAGGATAGTTCATAGCGTACGCAGAACGAGAACAAGTGAGACGTGTATAGTTGCCATGCTGAGTAGACCTGGCGTCTACCCCTCCAAACGCATTCTTATTGGCAAATGGAAGTAGCTTCCACAGTTTGTAACAAGCCGTGCCTGCGCATGTTATTTACCAATGGAGAATCGAAAA

>41 ERCC-00059 252 525

ATTTGATCGTAACTCGGGTGACCAATGACCATATACGGCGTATTAAGGTCGTACCCTCGGTCTCAACTTGTCGTATGGGACTTTCAAGTACCTTAGCTCGTCGGACGCTTTAGATGACTTATCCATAGTCCTAAGTCCGGCGCCGGTTAAGCCGCTATTAGCGTGTGTGGACTCTCTCTAGGAGCGGCTTCGCACAAATTACTGCTCAATCCTAGATACGTTGCGCTCTTTGGTAAACGGCTCAGATCTTAGCACTCGTGCAGTTCTACGATGGCAAGTCGTGCCTCGTTCTCGTGTAGAATATCAGCTAATAGGGTCGGCTCAACAGTGTATCCGGTGGACAAGCACTGACACGCGATGACGTTCGTCAAGAGTCGCATAATCTCAGAATCCGTACAGCCGCATCGGGTTCACGGCTATAAAACAGCGTCATCAGCGTAGGGTATCGCTTCGCGTGTCATGACTTGGGCCACGTCTCTTTCTCGCACATTAGGCTAGATTAAAA

>42 ERCC-00163 253 543

ATAGACTAGCCTGCCGGTCAATAACTGATGACGCGGAGTCAACCTGATAACCCATAGCGGAACAGTCTAACCTACGCGAGATACGTCTTACCGCACATAGGTAACCTATTCGTGACTAGCAGGCCTTATTCCGGTGCTATGAGTATCTTACCTGGTCTAGGTATCTAATTCGTGGGTCGGGTACTACATTCGTGCGATGGGTCCTCGCTTCGTCTATGAGGTCTCGTCTTCGTGAGTGCAATGTATCCGAAGTCGTAGTGATAATATGGAACTAGGCGCGATTTGACGAACGTATGCCGCATATTCGGAACGTCGCCTGGAAATTCGCCACCTAGATCGAAATTATCGGAACTCGTCGCTTATTTACGAACCTTGGGAGCCGTTCCTAAAGCTGAGTCTGGTTTCTTATTAGCGAGGAGCATTTCGTGAATACTGAGCCGAATATCGTAAGACACCCGCGAGCGACTGTAAACTAATCGGGGAACTTATTATAGGGCCGGTCCAGGTCTTGAACGACGTAAAA

>43 ERCC-00170 345 1023

TATTGGTGGAGGGGCACAAGTTGCTGAAGTTGCGAGAGGGGCGATAAGTGAGGCAGACAGGCATAATATAAGAGGGGAGAGAATTAGCGTAGATACTCTTCCAATAGTTGGTGAAGAAAATTTATATGAGGCTGTTAAAGCTGTAGCAACTCTTCCACGAGTAGGAATTTTAGTTTTAGCTGGCTCTTTAATGGGAGGGAAGATAACTGAAGCAGTTAAAGAATTAAAGGAAAAGACTGGCATTCCCGTGATAAGCTTAAAGATGTTTGGCTCTGTTCCTAAGGTTGCTGATTTGGTTGTTGGAGACCCATTGCAGGCAGGGGTTTTAGCTGTTATGGCTATTGCTGAAACAGCAAAATTTGATATAAATAAGGTTAAAGGTAGGGTGCTATAAAGATAATTTAATAATTTTTGATGAAACCGAAGCGTTAGCTTTGGGTTATGAAACTCCATGATTTTCATTTAATTTTTTCCTATTAATTTTCTCCTAAAAAGTTTCTTTAACATAAATAAGGTTAAAGGGAGAGCTCTATGATTGTCTTCAAAAATACAAAGATTATTGATGTATATACTGGAGAGGTTGTTAAAGGAAATGTTGCAGTTGAGAGGGATAAAATATCCTTTGTGGATTTAAATGATGAAATTGATAAGATAATTGAAAAAATAAAGGAGGATGTTAAAGTTATTGACTTAAAAGGAAAATATTTATCTCCAACATTTATAGATGGGCATATACATATAGAATCTTCCCATCTCATCCCATCAGAGTTTGAGAAATTTGTATTAAAAAGCGGAGTTAGCAAAGTAGTTATAGACCCGCATGAAATAGCAAATATTGCTGGAAAAGAAGGAATTTTGTTTATGTTGAATGATGCCAAAATTTTAGATGTCTATGTTATGCTTCCTTCCTGTGTTCCAGCTACAAACTTAGAAACAAGTGGAGCTGAGATTACAGCAGAGAATATTGAAGAACTCATTCTTTAGATAATGTCTTAGGTTAAAA

>44 ERCC-00126 565 1118

GGAGACCGACCGCGCATTAGTCAATCATATAGTTCGATAAATGAGACGTCACGGATTTGAGTATCATTTGCTAGAACTGCTTACCTATCTAAACGCTCAGGTATGCCTGCGCTGAGACTCAGCATCTCAATGTGACCGAGCTTGTGACTCCGCATCCTCCTGCAATATACCCATCGGCCCTCACGGGGTAACGTAGCTCTTCTGCGTACACCTGGCTAGAGGGTCCTTACCGGCTGTAAGCTCACTACAATCCAGGTACAGAGTGCGTTAACCGGCCATTAGAGGGCCGCTACACCCGTCAGAATTTAAACGTATGGGCGGCGAAGCGCGATAATGGTTAGACCTTCTTGCAGGGGCAAATAACGATTTTGCGACTGCCCCTTTAAGATGGAGAATGGACAAACGCTCATTCCTAGTGAGGACATCGACATCGTTACGATGCAACGTCGAGTGGCGGCATAACACTTGGCTCGGTCCTACCCGCGACAGTGCAACAAAAACAGTTGCTCTACATTCGCTATGCATTTTAACGGATATGCCTTTGCCCCCCGTCCGTCATGGACGAAAAGTTTGGCTATGCGCGGAACATTATGCCGGATTACTCGTTATCGCTGGGTAATCGTCCGTTGGGCCCGCTCTTACGCGATCAGTGCCGTTAGCACATGCGCGAATAGGGCGGATTCCGAAAGCATGTGCCAAATTCCCCATGACCCTGTTCCAGGTGAGCTGACACTATCAGTCGAAGTTGTTTCCTTAACCAGGAAAAACGCTGTTTCTCCGTTTGCTTGTACCATGGGGGCAGGGAATTCTTCGAGCGCGCGAATGTATACACTATTATCTCATGTGCATGTCCAGAGCGGGCTAACCTAATATACCTGGTCGCATCGTCCGAGATCAGTTGGGAATAGCGGAGCGGATACGCCTGGAACGCTGGGGCGTGCCAGTAAACTTACTGTTCTCCAGTTCCTCCATCATGTTCCGTCTAATCCGGAGATGTGTAGCTGCATTGTGCTCCGTTGCGGTACTCCTCGACTGGGTGCCATATGGGCTACTGGCGAGAGGAAATTTGCTTTTGTGTATTTAGGCCCGTGGCACCTA

>45 ERCC-00099 558 1350

GGGGATGCAAATGAAAGAGGAGACATTTTATCTTGTCCGTGAAGATGTATTGCCCGATGCAATGAGAAAAACATTAGAAGTCAAAAAGCTGCTTGATCGAAAAAAAGCAGATTCAGTAGCAGATGCCGTTCAAAAGGTCGATTTAAGTAGAAGTGCGTTTTATAAATACAGGGATGCTGTTTTTCCATTCTACACCATGGTAAAAGAACAAATTATCACACTTTTCTTTCATTTGGAGGATAGGTCAGGTGCGTTATCTCAGCTTCTTCAGGCGGTAGCTGATTCTGGAAGCAACGTTCTTTCCATTCACCAGACCATTCCGCTTCAAGGCAGAGCAAATGTGACACTGTCTATCAGTACGTCGGCATGGAAGAAGACATTCATACATTAATGAATAAGCTCAGGAAGTTTGATTTTGTAGAAAAGGTTGAAATATTAGGTTCAGGTGCATAAGGGAGAGAAAATCGTCATGAAAGTCGGTTATTTAGGTCCAGCAGCTACATTTACACATCTAGCAGTCAGTTCTTGTTTTCAAAACGGCGCCGAACATGTTGCTTACCGCACCATTCCGGAGTGTATAGATGCAGCCGTTGCAGGCGAAGTTGATTTCGCTTTTGTTCCTTTGGAAAATGCTTTAGAAGGATCTGTTAATCTAACAATAGACTATTTAATACATGAACAGCCTTTGCCAATCGTGGGTGAAATGACGTTGCCGATTCACCAGCACTTGCTCGTCCATCCCTCAAGAGAGAATGCATGGAAAGAGCTCGACAAAATTTACTCACATTCACACGCGATTGCGCAATGCCATAAATTTCTTCATCGACACTTTCCTTCCGTTCCATATGAATACGCCAATTCTACCGGGGCGGCAGCAAAGTTTGTCAGTGACCATCCCGAGCTGAATATCGGGGTCATTGCCAATGATATGGCAGCTTCTACATACGAATTAAAAATCGTGAAACGGGATATACAGGATTATAGGGACAATCATACAAGATTTGTTATCCTGTCTCCCGATGAAAACATATCTTTTGAAGTGAATTCAAAATTGAGCTCTAGGCCCAAAACGACCTTAATGGTCATGCTGCCGCAGGATGATCAGTCCGGGGCGCTGCATAGAGTGCTGTCTGCATTTTCTTGGAGAAATTTAAACCTGTCAAAAATTGAGTCACGTCCGACTAAAACCGGATTAGGCCATTATTTCTTTATTATTGATATTGAGAAAGCGTTTGATGATGTATTGATTCCAGGGGCCATGCAGGAGCTCGAAGCACTCGGCTGCAAAGTGAGGCTTCTGGGTGCATACAGTCTTACCAATTATAAAAAA

>46 ERCC-00154 269 537

TCACTAGATCAGAAGTCTCCCACTCGAGACTAATCTTGGACTATCTATGAGCACCTATTGCGCTGTGGAAGATTGCCCCTAGGTCTCTGGCGGCTCCGATTGCGGGATGAACTGGTTGGTCCGAGGAGGCATATAGGAAACGATGGGCACGCGCTATTCAGACGTTATTTGGTATGGAGTAAGAGGCCGGAAACTGGGCTCGATTGATGGATACTGATCAGTCAACTCAAGCGAGGATATCCATACCCACCGACGGTATGGTCATTAATACCCAGTATTGACTAGTCGGAGGTCTAATTTGGAACGTATTCCGCCGCACACACGAGATTCACTTACATGGACGTGAAGATTGATCGCCGGGCGACTATTATTGCACGACCTTCGCCTGCCCTCAGCTGCGCCCTTTTTGTCCACGACGCAGGCTGGACCAAGCAGCCGAGGCTGCGCTCCGGCATCAAAGTCCACGAGTTACAGCCAGCGGGTTTTAAGGGGGTATTAGCATCTCGAGTGAGTAAAA

>47 ERCC-00160 339 743

TCCTTGGTTTGAAATCAAATTACTATAGATTGCATGCAGAAGTAGGGCTTTACAAGTTTGTTCCTAATTCCGTTGAACAATAGCGAATGTATGATGGTCGGTCATTGCCGCAACCCTGAAGCAGTGAGCGTGGTTGAGTAACCACGTACTAGAAGTCCAAGGATGGTTGCAGTAGCTAGCTCAATTGCCATTGCTGGAATAGTGTCGGTAAACCACGCACCGGGGAGCCGCGTTTCGTTGCGATAACCTCATATAGTCCCAGTCTCGGACGCGAGCACCTGCAGCGTAATTAATAAGGTCAGGCTATGATTCACCACCGTGTAGGAGTTATGCGCCTTATCGCGAAGGTAGTTACTCTTGCCAGTGGGCATGAAATTGCGAGCTTGCCCTAGTAGCGTTAGTACCGTCATAAGCCACTCTAAGAGATCGAAATTTTATACAGTAGTACTAACAGCAGACCTGCAATATCAAAGTTATAAGCGCGTCGACACGCCTCGTCTAAGAAAAACTACTTCCATTAAAGTTCGAGGACAAATGCGGTCTGATTCAGTCCTATAGCGAGGCCATTGCAGTGTGGTCTGCCGTGGTCGGCTCGATAATACCATGATAACTACTGACTGCGCAGTTGTAGCGACCGCAATGGGGAGTGTGTGTTCTTTATTTCAACTGCGTGGCTTATCCTACTTAGGAGTTGTGGATACCAGATTATCTCGCGGTGGAAAA

>48 ERCC-00085 410 844

GATTCAGCCTGACGTACATAGACGAGATAGGGTAAGCCTATTGCAAACCTCCGCTTTGCTACTACAAGGACCTCGGAAGTGTGGAGAATCGAACCTACAATTTTGCCAGGATCACGCCGGTTATCTCTTGCAATTCCTAAGATGGAGGATAGTTTTGGAACCCAATATCCCCAGCCTACGGTTTAGGTGCAAGAATAGAGCGTACCAGGCGTCGCCCGCGGCCTTATGTAGTCTGTATTGTTGAATTAGCCTTCTTCAGAGGGCCTAACGCCAGCACAACATGCTTACCTGGCTAATTGACTAAGTGCTACGGTACACAACGACTACGTACGTAGCACACAGTTGAGGTTATCTCTGAGCGCCCCGTTAGAACTGAGGGCTCAATTATAGTACCTTAACTGCGTTGCGCTGTCATGGGAAGGTATTTTAATACGGATTCATTACCGCTCCACCGGCAGCGTTCCGTTACATCCAACTAGGTGGACAAACCTGCTCATTCAAGGGCGCCTCCATCGCAGGGTTGCAATTTATTTTCTAGCTAGAGGTGTAACCATGCCGGCAGCGAACGTCCGCACTAGCAGGTACACAATCAGGCATCGAACATGTACTCATTAGTTCCGAATTATAAGTCGGATACTTGCCTTAAGTCGCATCTGAATCATAGCCGCTCCATTGCCGAGTAGACGGGATCTGCTGTAGAATGGTAATCAGTCTTACCCGTGGGGTCGTTCTGCCCGAGTGCTGCATTCCGCCTACGCGCGGTATTCAACAAGGGTAATCCCTCCGACAACCCTCAGTGTTATCATCCGCGTCAAGGGGGAAAA

>49 ERCC-00034 501 1019

GGTATTACCCAGCACTCGTATGGCGCCCCATCGTTGTATCAGCAGGAGCATATGTTGCTCTTGATTGTATCACTTCGCGAGAAAGACCCCTTGAATACACGAGCTGCCGTCCGGATAACGATGTAATAGCCTATGGAGGGGAAGTAGTCATGTCTGCGGACTATTGGTAAGAGCACCGCCTCGTCGTACGTGGACACGAAGCTGTTCGGCCGCACGCAAGTACCTCCCACTTAGAAAGCGAATAACCCAACGACCGTGTTCAACCCTGGCCGTCTCTCAACCAGGTATGCAATCAACGACATTGGCCCAGATGTAGGGCCGTCTTGGTGGATTGAAACTCGGATCGATCACGTATGGTCTAGACCTTATACAACACCTGTGCGTGCGTCTGCGTCTGGCGATGGTGGGTAGCGGTCCGGACCACGATCGTACTGTAGGCGGCCCAAATGCGGATCTTTAGGTTGACCGATTGTACATCTCGCCATAGCCCTTTTCGTCACAACCTTTATAAAAGGGGTCAGGCCACTGTGTGAATCAGATGGCAAGCCCGTATCCGTATAGAAACATACGTTTCTCTGGCCACGACCAATAATTATGCACTGTTGGCTCGGAAGCGGGTCTACGGAGAACATTAGATATCGACCTAATATCCTGATAGCTAGCTTTCCCGAGGATAGGCGAGGATGGGCGTGCTAGACACAGGAGCATTGCATCACATGAATAAAGGGCTACGTACGCAGCGGCGTTAAGACAGACTTAGACTGATCACTATGAAATGATAGACACCATGTCGCAATTTGCTCCCTCACTCATATCGTTGTATCATAAAGTTGATCCTAATCACAAACCGAGTAACGAGCCAGACTAATCCGGAACATTCGTAAGCGATAGTGGTTGGCTCCTCCCGGTCGCCGAACTGATATCATAGCTAAGTATGCTGGCCTCGGTTAACCGCGAAGGCATATACAATGGATTCGCTGTCCGCGTAGCAATATAAAA

>50 ERCC-00157 511 1019

GGTCTTTGTCACCTCCGTCAATTTGTATTAGAACCCGTGAAGGCCCAAGTAACAGGCCCAGGGTTAACATGTACGGAACATACTCCTTCCACGGAAGATTGGGGATGAAAGTTGATACCCAAACTTCATTAACACAAAGGCGATGTGGGCCGAGTACTGTGCTTACACCAACAGGGCGGCTCAACTGGGTTGGTAGCCAGCACTAGCTTATTCACAATTAAGGCCGTATGCATTCTACTGCTTATCCGGTGGTGATTGCAGCCAGGGCGGAAGTGAACACGCTTGTACGATGTGTTTGCATAAGCGGTTACCACAGGCGCTACTCTCGTCGATAGCCGACTACTAATATTCAGCCGGCGCCGGTAGATAGCGAGGCTTTGGGGGTAGCTTTAAGTGCGGTCTAGGCTCAGTTGACGATACTTACTTAGGCAGGGTTACAACCCTTATGATGGGGTATGAGGCACGTGGCCATTCATCCGGACCCGATGCTGTCGTGCTTCTCGTTGGCAATAGCGCGGATTAGTACAGGTGACTAGTTCAGCTGTTGTTCGGATTCCAAGTAAGCTCGCATAGAGCTGGACTTCTCGGAACGGTCCTGACGCATTCCTGCATCAATACGCGGCACCGGGGGTCCGATAGCATCTCGCCTTAGATCCGGCGGGGGATACTTGGTCAAAGCTCACTACGGGACTAGAGTGGCTAGTGCAGATGCGCAGCGCAGATATGCTATACGAGATGAGCTTCAAATTCATGGAGTTATGACGATATAACGCTAGGATCTGACGCAGTGACACCGGTCGTGTGACAACTGGGCTTTAAGTGAGGCATCAGAAGTATACTTTTAATGGTGCCGCTCCCAAATCCCCGATCTTGCCACGATTGCCTAAGCCGTCATGTTAGAGGCGGTCACAGCAAACCCTCAGTTTACCGGTTCGATGATTATACGATGCCGGAGCGAACGACTACGCTCGAAGTTTGGTTATCTAGAGCACGTCAAAA

>51 ERCC-00077 90 273

AGTTTTTTCACCTTATGACTTCTCACTCCATACGAAAGACAGATGGTTTGCTCCCAATGAACCCAGAGGGAGTTAAGGTTGATGATTCCCAAGTCCAACATGGAGTTATGCCTATAACGTCATGTTGGACAGACCCCTGTATTGAGCAGACTTAACTTATACTGATATACAGCTAAAATTTGAACAACAAATCTCTTAAATTTATTGTTGAGAATAAAAAGGATACTATCTTATCCAATATTGTATTTCCAAA

>52 ERCC-00067 304 644

GCCGTACTTACACCCTACGTTGCTGATCTCGGCGCTCGTACATTTACACTTGTAGTTGAAGGTCGGGCGATGAGGCGTTCGTTTGGCCAACCTCGTTACATTGCACAAATGCCGGATTTGGCTACTATATATACTCCCTGATCAAGGGTCGAGTTGTCTGTGCATTAGCTCGGAGCTAATGAGCACGTCGGTCACGGACACTGCGAAGCACGACGAAATGGCTCTCATTGCGATTGTGGCCCTTGTTGCAGACAATTGTCAGGCGGGACGGGCCAGGAATGACAATAGAACATTAATCTAATTTACAATTTGACGCCATGACGTATTAAACATTCGGGCATGCAAATCACCGATTTTAAGATCCCCTGTACGGCTGCGTCCACACTAGCATGATGTAAATCGATTTGGCGGTGACCGCTGATCCTTAACAAAAGGGGGTGGTTCAAATGTAAAACAAGTAGGTGCGAGCAAACGTCTGGTGCTCTAATGGCAAACATCTGCAATCAGCGAACCTCGCGGTATGGCAACTCACTAATTGTCCGTGCGACGACAAATTACCGAGGGAGGCACTTATGAGTTATCTTCGCTGTGATATCTCCTTCGACTAGCGCGGTCAGTCCAAAA

>53 ERCC-00039 362 740

GAGCATGGCCTAACTGAATGCGCCTGCAGTATCTTTCTTAGTATATCAAGATCCGTAATATAACGGTTTGCGCGACTACGGTTACCGTCTTTATAAGTGAACAAAACCGGCTACCAGCATGTCGTATTTCGCCACCCATATAAACCCCACTTCGTCCTCAAGGAATCCAAAAGTCGGACGGCGTGGCTTGTCATCTTCGTCAGAAGGTTCGAAACCAGTAAAAAGACGCGATAGATAGGCCTAAGTGGGCCTCCCCTTTGCCAAAATCACGACGAAGTGACTAGTGCAGGCGTCGTATACTTACTCCTCATGTTCAGCCCACGCTGCATTGGTAGGCTGTTAGAAGCCGTGACCGAACAGGGTATGATGACCTCGCCATGGGCACCCTTGTATTCTCGGGGGCGGATGTAGAAATCAAAGCTGTTTACTAACCATGTACTGTCCTAAGAGATTGGCTGTGACCGGTCCCGGACACTGCGTCAACGCAGGTCGCTCACGATCGGCGTGCGCATTTTCGTGAATCATGTATAGTGGTTCTCGTTAGATACACATGTAAGCTGAATGGGGCCCTACCCAACCGGTTGGGTCTATAAGGCAGATGTGCGACGCACCTATTGGTAGGCCAATCTTATGGTTTGCCTCTTGTACTGAGGGTACCGCAGAGGTCCACTGGTTAGCTCACACTATGATCTAGGAGCAGTTGGGCGGTAAACGGCAAAA

>54 ERCC-00150 349 743

AGCTTGATGTGACATCACGTCCCAATCAATTTGGTTTTACTCCCCTCGATTATGCGGAGTTATCAGTAAGAGCGGGTACCGTTCTGCTGAATGCACTATGAGGGCGACCCTTGATCTTCATTCGTTCCATAGATGCAAATACCCGGGTACTAGTTGGCGAAGTTTGGCTAAAGATTGCCCACTTACTAGGTGACTGTTAACTAGAAAGTTGCATGAGGCTGGTAAGTGAGGGGCTCAGTGAAACAGGATACACCGTACCGTGACGATTCCCGTGTGGGCTTGACATTCAGCCAGCCTCCCGCTTGTACAGAGGCAGACTGTCCGATGCCTAGTATGTTCACTATACGATTTCTTGGGCTAGCTAGGTGTCTTGACCCCATATTCCAAAGATCCGAACCTGCTTGTACTTATCAGTACGAGTTGATGCTCTTGGGGTACCTATTTTTTGATCCGATAGTATTGGTTCTGGCGTGTTCTCGTAACCCCGGGCACTAGATTATGGTGAATGGTGTGTACACACTACTTGACGGGGCGTTACCATTTTCAGGTGGGCACAAATAGCTAAAGCCTCGTCCGCATTACGCCCCATGGCTCACGTCTCAGAGGAGGCTAGCTCGAGGTCACCCATAAGGGATTACGGAGTTACACCCATAGTAACAGACGGAAATTGTTGATAACAGATAGTTAATACGCCTTGGCTGAGGAGACCAACGGACTTCAAAA

>55 ERCC-00143 381 784

ATGCAGCGTAGGTATCGACTCTCACTGTGGAGTCGTCTATGATGTCGTGGAGTCCTCTCAGAGTGCTGTAGGTCCTCATAGGTCGTGCTGTCTCTCTACACGCGTGCGTGAGTCTACATTTCTGCGAGTTGGTGCTCTCACTGCGGTGTCAGTGATCTCTCCGCGTGTGACATGAGTCTAGCTTCGCGGTCATGGTCTATCCCAGCGATGGATGAGACTACTCTGTACTAGATGGTCATGCCTGCGAATGAGTCGTCAGTGCCCACAATGTCTCGATAGTGCGCCGAATGTGTCTGTAATGCCTCGAATGTGTAATCGTCAACTCGTATGTGAAGTGCTAGGCTAGTATTGACATCTACGGGCGGCTATTGACGAACTCTCCGGTATATGCTCTACATCTGCAGGGAATTGCCGACCATATATGGGTCTTGCTGATACGCTAGGGTGCTTGCTACTTAGATAGGCGTCTTGGCCGCTATTCGCGGCGTGTCTCAGAATATGCGCGACGTGTCTGGTATATGGCGACTGTGTCCGTCTATACGCATACTGGTCCACATATAGACATACTTCCACGACATGACAAAGCGTGCTCCTACATAGCACGAGCGTCTCCTAAATAGATCCGGTCTTATCGCTGAATGTCTAGGATTCTCGTCAATGATCTACGATCCTCGCTAAGTATTCAGCCACCTCGTATAGTATTCGCGCACCTGAGGATTTATTCACCTGACTCGCGTATAATATGCCGTCACCTAGTCTAAAAA

>56 ERCC-00028 572 1130

AAGAAAGTATTCCATTCCGGCTCATGGTCCCGGCTAGACCTGCAAGATCGAAGGTACTCAATGACACCAGTGACTGAGCGGTCAGCCCGGAAATAGCCAGAAATGTTACCATCCCCGCATGTTACACAAACGTCGGGCTGACGGAACCCATGAGAACCTATGGTGAATAGACAGTAAACGAGCGCAAAGGCGTGCTGCCAAGGGCTCCCACCGAATGTAGAACTGATCTGATTTCCGTGACAGGGAAACGCAATGCGAGGTTCCGCAAGTCCACCTATAATCTGTGACCGCTCGTAGAACATGGTTAGGGCCTGATTCAGTTAAATCAAGCCACCTCTGACAGAACGACGAGTCAGTGGAATCGACTTCACACCTCAGAGTCCACTCACGTGCTGTAAGTCAAAACCCAGTGAACTTCTCAACCGTGTAGCGCTCCTAGAACATTCAGGCGCCACTGAGGGGCATATGGATGAAGCGTGATACGAATTACCTCCAACGAATGCCAGCTGGGAGGAGGATAGGGACTATGTCTTCGTCAATGCTCCCGTCAAATGCCTTTCTAAATACCTTTTCGACCTCTCTGTGTGGAATGGGGTCTAGAACCAAGGACTAAAGCGGTGCACGAGGCCCTTTGGATATGTCCTTCTTGGGAGGGCCCACAGCCAAAGCCACCCATCACAGCTGGAATCTAATCTTGTGACCCAGTATTAACCGTGAGATCTACACAACCGACAAGCGCTAGCTTCCTCCCCGGTCAAGTAGTAATGCCGGGATGACTTTGCGTGTCCTATAGCGATCGAGCCCTCGAATTCACGTCGTCAAAGTGGAATGATCAGATTAAAGGGCTGTCGGGAGGGATGTGTAGGCCACTAACACCCCTCTGCACGAACTATAGATACGTCTTTCATCGGTACGCTCGAAAGCGCAGGTGGCTCCCAAATCGTGGGGAGTTAATCGAGCTGCGGATTGGTCCCCACGCCTTACGGCAGCGAACATAATCCCGCTGATGTGAAGTCGATCTACAAGGTAAACAACGGGGAATATAATTCAGTTGAACCGGTGTGGAGCCTGCACTTGGAACGCTGCATAAGGGACCCAACAGCCCCAAAA

>57 ERCC-00014 865 1957

AAAAGAAGAAGGAAAGTAGAGGAGATCAAGATGTCAAACGAAACAATTAAATTAGTCATTGCGGGACCGCGTGGAAGAATGGGGCAGGAAGCTGTTAAATTGGCAGAACGAACACCACATTTTGACCTTGTAGGGGCCATAGACCATACATACGATCAGCAAAAATTATCTGATGTGATGCCTGTTGAGTCAGATGCTTTCATTTACACAGATATCCTTGCCTGTTTTACAGAAACACAACCGGATGTCTTGATTGATTTAACAACGCCCGAAATCGGAAAAGTACATACAAAAATTGCATTAGAGCACGTAGTCCGTCCAGTTGTCGGAACAACCGGTTTCTCAGAAGCTGATTTAAAAGAGCTCACATCTTTAACAGAAGAAAAAGGGATCGGAGCCATCATCGCGCCAAATTTTGCGCTCGGTGCGATACTGATGATGAAATTTTCAAAAATGGCTGCCAACTATTTTGAGGATGTTGAGATTATTGAGCTTCATCATGACCAGAAGCTTGACGCACCAAGCGGAACTGCGCTTAAAACAGCGGAAATGATTTCAGAAGTCCGTAAAGAAAAGCAGCAAGGACATCCGGATGAAAAAGAAATTCTCCCAGGAGCAAGAGGAGCGGAGCAAAACGGTATTCGCTTGCACAGCGTCCGTCTTCCGGGACTGATCGCGCATCAGGAGGTCATGTTCGGCATGGATGGCCAAACGCTTCAGATACGCCATGATTCTTATAACCGTGCTTCTTTCATGTCAGGCGTTAAACTGTCAGTCGAACAAGTCATGAAGATTGATCAGCTTGTGTATGGTTTAGAAAATATCATTGATTAGACGGGGGGATAAACAATGAAAATTGCTTTGATCGCGCATGACAAGAAAAAACAGGATATGGTTCAATTTACGACTGCCTATCGGGATATTTTAAAGAATCATGATCTATACGCAACCGGAACCACAGGGTTGAAAATTCATGAGGCGACAGGTCTTCAAATTGAACGTTTTCAATCCGGCCCTTTAGGGGGAGACCAGCAAATCGGTGCACTGATCGCTGCCAATGCACTCGATCTTGTCATTTTTTTGCGCGACCCGCTGACCGCGCAGCCGCATGAACCGGATGTCTCGGCATTAATCCGTTTATGTGATGTGTATTCCATTCCGCTCGCCACAAATATGGGTACTGCGGAAATTCTTGTGCGCACACTTGATGAAGGTGTTTTCGAATTCCGTGACCTTCTTCGGGGAGAAGAGCCGAATGTATAATGCTGACGTTCTTGCTTTTGGCGCCCACAGTGATGATGTCGAGATCGGAATGGGCGGCACAATAGCGAAGTTTGTCAAACAGGAAAAAAAAGTAATGATATGCGATTTGACAGAAGCGGAACTCTCTTCTAACGGTACGGTCAGTTTGCGTAAAGAAGAAGCAGCTGAAGCAGCCCGCATATTAGGCGCAGATAAAAGAATTCAGCTAACGCTTCCAGACCGCGGCCTAATAATGAGTGATCAGGCAATTCGGTCAATTGTCACTGTCATCAGAATCTGTCGGCCAAAAGCGGTTTTTATGCCGTATAAAAAGGATCGCCATCCGGATCACGGCAATGCGGCTGCACTGGTGGAAGAAGCGATCTTTTCCGCCGGAATCCATAAATATAAAGACGAAAAAAGCCTTCCGGCGCATAAAGTCAGCAAGGTTTACTATTATATGATAAATGGTTTTCATCAGCCGGATTTTGTTATTGATATCTCGGATACAATAGAGGCAAAGAAACAAAGCCTCAACGCCTACAAAAGCCAGTTTATCCCGTCAAAGGATTCCGTTTCTACTCCTCTGACGAATGGGTATATTGAAATCGTTGAAGCGAGAGAAAAGCTTTACGGTAAAGAAGCGGGCGTGGAGTATGCCGAAGGTTTCTTTTCCAAACGGATGCTGAAGCTTG

>58 ERCC-00134 86 274

AGGCTTTGTTATTGGTATTGACTTACAAACAGTTAAGCCATTTGAATATGATAATGTAGTTGCAATAAAAGGAGATTTCACCTTAGAAGAAAATTTGAACAAAATTAGAGAGCTAATTCCAAATGATGAAAAAAAGGTGGATGTGGTTATAAGTGACGCCTCCCCTAATATAAGCGGTTATTGGGATATAGACCACGCTCGTTCAATAGATTTAGTAACTACTGCCTTACAAATAGCTACTGAGATGCTAAAAA

>59 ERCC-00058 570 1136

GGGAGAGTAGGGCCCACTTGTCGATTCGTTTGACTAAACATGGAGAAAGCATCCGAGGGTGGGCAGATCCGCCTAAGCAAGGTACCATTTAATCCCGGTCCCACGAAAGAGTTAACCTATACGCTCAGATGGCGCCATGGTGCGTTCGGAGCCACTTTCTGCGAATTGTCCACCACTGCCCTCTCGTGTGATGATCAACATACTATCTTCTGCGGAGACCACCACTCACGTGTGTAGGTGAGCCATGTTGAATCCATCAGGCACCCGTCCCGGCGTATGAGCGTCTGGAGGCTCACCAACAAAACTTACATAAGACCGGAACTCGAGAGTCTGGCATCATCGTGAACAGAGGCACGAGAGTGAGCTGTGAGTCCTGACTGAGCATGTCTCGAAAACTGTGATAGATTAAGCCATGGACCAAGGACCAAGAGATCGACGGGCTTGGATATTGCCGCAAGGCTGAAGGTAACACCTTCAGCTTCAAAATGCTTGAATACTCAGCCGTCCATCACTTCGACCGGGGTTAAGCTAAGATCTGGTGGCGGTAGCCCGTCAAACGAATTGGCATCGGACAAGACAAATCAACCAAAGAATCTACGGTTGCCATGCCATGGTCTGCGTAACGACACCCGAGGTCCTTTTTATGGCGCCAGGCATCGACGCTGTAAAACCCTTCCGTTGTCCTGAGCAGGTTATAACGACATCAAGCGACAGCGACAAAGAAGGCGGGAAAACTGTGGACTCCCGGCTATGTCAAATACGCTGAGTCACCCAACAGAAACGTTATCCGCTTGGCAAACGAATACATGGAAAGTGGGGCCGGTCGGATCACCAAGATAAGTGGTGTCACGAATAGCCCTCGCTTGCTCTAATCATGGGATGGTACATCGTCATTGCTATTGGATGCAAGACGAATCCTGGCAACAGCCCTCCGGACTCAGTAGGTCCATGGATTTAACTACAACAGGAAGCCGGTCAACTGTGGAAGACTACCAGTATTCGAGCTGGATAAAGAGGCTCGGCACCATGGTATAGATGTAATAGGAACCATGAAACTCCCATGTCGCAAGCACTAACAGAGCTGTAACCTCCCTCCATTAGCGGTAATCGGGAAAA

>60 ERCC-00069 571 1137

GGGAGAAGTACCAATATCAGACTCCCGAGACATAAGGGGTGTGCACGGCATGGATGTGTCGCTAACCTCCTGGTCCTATAAAGCAACACTCATGGCACCGTGTGCTGAAGGCTCCAAACGGGTCGATACCAGAAGGGTCGATTCTTTCTTCCAATACATTGTCGGTGTCAATGCTGGAGGCCCAGGAGACGAAATCCTCGGTCAGGTTGTTACTTGAAGGGTTCAACACGAGCTCAAATTTGTCCAGGGGTGGTCACCAGCGGGTCTCGAACAGAGGTGAAACGGGATAAATACCCGAAACATTCATAACCCTGCCAGTCATTATAAGCAAGCCGCTGACTTGGACACCACAACGTGTGGAACCTCAGGTAAGCTGGTCCGGGAATCTTGGTCGCGAAGCGACCCCATTGAGTAGGCAATCCAAACCCTGCAAACAATAGGTGGCCATCCGGTCAAATAATGTGGGAGATGCGCACATCATGAGAGCGACGTATTGAACTCAAGGGACGGTGGTCCCAGGAGAGGGATTACCACATGTTGAAGCTCTTTCAAATCGATTATCCCAATCTGCGCACTGTAAGGTAACCAACCGCAATAATCCCCAGAACACTGCCCTGGTATCTATCGATTTGGAAGTCCAGACTCTAGTGATGCCGGCTGGTCGCGACAAGAAAACATAATTCGCAAAGGGCCCCTGTCTCATTTGGCGTCTTGTACCCAAACACCATACTTGGGAGGGTGTTGCGGCCGCATATCAACGACTAGAGGCCGTCAGAATGAGTCTTCAACACATATCGCTGAAGAAATGTCACTCCCATGGTGGCTGATATAGAAACATATCCTCGCGGACTTCATCGCCGGGCACAAGGCTACAAGATGGACTGGAACGCCGTGTCAACCTCGTAACAACTCCCCATGCAATTCACTCACCGAGTAGCCTATAATGGGGAATGTAGACGGGGCCCTATCTCAAATGTGTGCACCTCTATCCCCACCAAGTATAGTCGACTTAAGGGCGGATCAAATGGCGCGCTGCCATACAAATCCGGTTCCAAGCATGACTCATGGGCAGTACGCCGGTTAGTGGGAAATACGGCTGTCAAACGCTGGTCCAAAA

>61 ERCC-00031 544 1138

GGGAGAAAAGCTTCTTGCCTAGTTACAGCACGAAGATTGGGACCCATCGATCAGCGCCTCCGTGCAATGGCGCCTTGGTACACTCTCTAAAATGCAGTCGGTAGCGGGGCATGTAGTTACTGCGGTGATATTAAGCTAGTGAGTAACCTTATATCGAACTGTCTACCAAATTGATGTATCTACATTAGCCGTACGATGAGACAATGAACCGTGTCTACGCTCGGGAGCATTACCCCTATCATGAGCCTACAAGCACCTGACTAGACCAGGAGACAACTTGAGCGAGACACTAGCGAGAGGTGCCGATATAAGGTACATTGTTGCGGATGTCAACATCGCATTTTTATCCCCTGGCGGGATGCAACTAACACCAGTAATGTCGTTCTCCACGACCCCCTACCCGAAAACTTACTCGTATGAGTACTTTCGCGACGACTCGGCTGTGTAGTTTCCAAGCAGGCCCCTGGCTAGACCCATTTGTACCACGCGGAGGATCATCGGAATCCATAATCGGCCACAAACTAGCCGGCTGGAAACCTTGTGATATCATGAACGAGGAGTAAGAGCTGGTTAGTGAATCACCTTGAGGGCGAGGAACACAACAATTCTGCGGGTTAGCAGGAAGGTTAGGGAACTCGCTCATAGCATATTAGTACCGCTATCTCCTTTCCTTGGCGCGACATCTCCCAAAGTTAGGCTGATTCTGCCGCCTGTGATCGCTATCCTTATTTGTGGCAGAATATGTCGATGCAAAAAACCTTAATTCGTGCTTCATACATCATCCCCGAGAACTCCACGGCTCCACTCCCGCTTATCACGCGTGGGTGCTAAATGATACGAAGTGTGGCAGTCTATGTTAACTTGGAGCATCATATTTATTTCTGTTCACGGATGAAGGCCTATATCAATGATCATTAAAGACATTCCGTCATCGGCTACATGCAAAGAGGCTTTTCCACCCTGTTTCGGTGATTCAGGTGTCGAGCGACCATTATCTGTCGGCTCATCACCGAACCGGCCGTGATAGAGTCTTCGCATCAGGTTACACCATGTCGACTGATAGTAACTGTGGTTCGAGTTATGCGTGTTGACACACCGTTGAGGCGCCCTATTTAAAA

>62 ERCC-00033 663 2022

GCACGCCCTATTTAACTGGAAACCTTCCAAGATTCGGAGAGCATATAAGTTGGGCCGAAAGCTCAGCTGTGAGCTTTGCAAACTCTGTCTTAGGAGCTAAGACAAATAGAGAAGGTGGGCCATCAGCATTAGCAGCTGCAATTATTGGAAAAACACCATATTATGGATATCACTTAGATGAAAATAGAAAGACAACACATATCATTGAGTTAGATGGACAATTAATCTCTAACTTTAAATATGGAGAGAGTTTTTATGGAGCTTTAGGTTACTTAGTTGGGAAGATTGTTAAGAATGGCATTCCATATTTTGAAAATCTATATAAATTAAATCCAAATAACGATAATTTAAAATCCTTGGGAGCTGCAATGGCTGCAAGTGGTGGTATCGCCTTATATCACGCAAAAAACTTGACAGCTGAATGCAGAGTTAAAGAAGTTGTTAATGATAAAATTGAAAAGATATCTATTGGAGTTGAGGAGATAAAGGAAGCTTATGAAAAATTAAATACAACAAATGAAGAGCCAGATTTAATTTGTATTGGTTGCCCTCACTGCAGTTTAATGGAAATTAAAAAAATTGCTGAACTTTTAAAAAATAAAAAATTGAATGCTGATTTATGGGTTTGCTGCTCTCTTCATATTAAAGCAATAGCAGATAGAATGGGATATACAAAGATTATTGAAAAAGCTGGTGGAAAGGTAGTTAAAGACACCTGTATGGTTGTTTCTCCAATTGAGGATTTAGGTTGTAAAAGAGTTGCAACAAACTCTGGAAAAGCTGCTGTTTATCTACCAAGCTTTTGTAAGAGTGAAGTAATTTTTGGAGATATTGAGGAATTGTTAAAAGGGAGATAATGCTGAATCCAATAATCTTATTTTTGGCTATTATTTTTGATAGAATCATTGGGGAGTTGCCAGAGAGTATTCATCCAACGGTTTGGATAGGGAAGTTGATAGCTTTTTTAGAGAACATATTTAAATCTACAAATTGCAAAAATAAATATAGAGATTTTTTGTTTGGCTCACTAGCAACGTTTATTACTCTATTAGTTGTGGGAGTTATAGCTTTTTTTGTTGATAAATGCATAATGCTGTTACCATCTCCTTTAAACTATATTATCTATGGTTTTTTGTTATCAACAACTATTGGCTACAAATCATTATTCGAATTCTGCAAAAAGCCGATTGAATATATAAAAAATGGTGATTTAGAGGGAGCAAGGAAAGCTGTTCAGCATATAGTTAGCAGAGATGCCTCAAAGTTGGATAAAGAGCATGTATTATCGGCTGCAGTAGAGAGCTTATCCGAGAACATAACAGACAGTATAATTGGAGCTTTATTCTATGCTATATTTTTTGGTTTGCCTGGAGCCTTTGTTTATAGGGCGATAAATACATTAGATGCAATGATTGGTTATAAAAATGAGAAATATCTATGGTATGGGAAGTTAGCAGCAAGGTTGGATGATATTGCCAATTTTATTCCTTCAAGAATAGCAGGGATTTTGCTAATAATTACTGCCCCATTTTATAAAGGAGATGTTAAAAAGGCAATATATGGGTTTTTAAAAGAAGCTAATAAGGTTCCATCACCAAACTCTGGTTATACAATGGCTACATTGGCAAATGCATTAAATATAACTTTGGAGAAGATAGGATATTATAAACTTGGTAGTGGGAAAATAGATGTTGAAAAATCTTTAAACGCTTTTAAGGCAGTTGATTATACAGTCGTTGTGTTTTTAATTATTTATACCTTAATTTGGTGGATAACATGATAAGTAAAGCTTATTACACTACAGAGATTCCAGAGGATAGATTTGAAGCTCTGAGTTGTATTAAAGATAGTCAAAAACCTCTTAAAATTATATTACTTGGAGGAGTTGATAGTGGTAAAACAACATTAGCTACTTTTTTGGCAAATGAGCTTTTAAACTTAGGATTTAAAGTTGCTATAGTCGATAGTGATGTAGGGCAGAAGAGCATTTTACCTCCAGCAA

>63 ERCC-00109 244 536

TAGCGGAGTGTCAAATTTCGGAAGGGGGCCATAATGTTTCTTTACAATCGCACCAGTTAGCGTGGCGTATACCATGTTGTTAACAGCGCCATAAGTGCCTGATCCGCGGGCAAAACTACGCAACACTGTGACTGGGTGCTAGGTCGACGAACAACTGACGCAACTAGATCATGGGAGTGCCCCAAGTAAAAATTGTGTGTCAGGGCAACAAGTGACGATAGCGCGCGTAAATGGTATCATGATTGTGACCTCGGTATCTCTTGTACAGTTTACGTCGACGCGAAGTCTGATCACGTTTAACTAGCTCAGGGGTATTAAATAACCGAAAGGTTCATGTGGATGTGTGAACTTGCAGACAGAATGACCCATAGTCCTTTACCCAGGTAGCTGAGGGCGACGCACTTGACCATCCGAATCAAACTGAGAGATCGAAATAGCCTCACCCTTGAACCTACAAAGCTCACTTTAGCCCGTTAAGTTGTCGAAATCACTAGATCAGAAGTCTCCCACTCAAAA

>64 ERCC-00120 259 536

GGCGTCTCACCTGGCTTGATTCAGTTAATGGAGCACTTGTGGGAGTGCCGCGACACAACATCCGCTAGCCTCAGTCGAATGACAAGTTAGAACAGGAGTGGGGCCGATCTCTGCAAACTCCTATTGTCAGGGGTGGTGGACGGTATAGGGTTTTGCGCCTACCTGAATGCAAGGGCTTACCTCCAACGGCTTAGATGTGCCTAGAAGGTACGCCCTTCAGTCAAGACCGGCCCGGCGTTAGTTAAAGCAGGCTTGTCACACATCACGTAGTTCCTGCTGCGTTTTAAGTCATTAGCTCCCAGTAACCCATCAACCATTACCGTATAGACTTATCCGAGTGTGATCAAATAACGCTGAGCCTTATGATCCTCGTCGACCCAACAACCGCCGGATATACGTTGGGATATAAACGAAACACGTACCGCGCGAGTGACACGCGTTGACGTTACATGGTAAACTCGTAGCCAACCTTAACATTCCCTGCACTATGTTAGTGCGTACAACATTAAGGGAAAA

>65 ERCC-00137 266 537

CAAAGCAAACCTAATGAAGCCAGACACGAGATCACAGAAAATCGCTGACTATCCCGAGTGCGCGGCGAGCTACAATCCAAGATGATTATTTTTTCACGACCGGGCGCTGGTCAGGTATATCGCTGGTACAGATGGCGGCAAGCATCGTTGTCACCACTTCCTTCACGATTTCCGTTAGAGCCGAGTGGTACAGCACGTACGTTATATATGGGAGTACGGACATGCTTCCCACCTCGTCAGCCAAGATGATAGATACCCGTAGCGTGATGGTCTTATAGCTGCTCTGATGGACTTCGAAAGATCACCGTGCAGCTATTCAAAAAAGCAGCCGGGGAAAAGGTGGTCTCGCCCGAAAGCCATGACCTCCGATCACTCCTGGCCGGTAGTCGGTTTATTGCATATTGTACAGTTGCGCGCGCGGCAGGTCACATCCCCCTGGTTAAATAGAGGCACGAGCGCTCGTTTTTGGGTGATCAATATGGCTTACCCCCGAGAGAGTTGTGAGCTTGCACGAAAA

>66 ERCC-00073 284 603

TGTGATAATTTCGACGAGGCGTTACATATTCTGAGAGGGGTGATTAAGTCTGCTTCGGCCTGGGATGGTCTGTCTACGTGTGCGTAGTTCTGTCATAGCGTCGAGGATTCTGAACCTGTCCATAGTATCCTGTAAGCGTCCAATGTACCTATATCGTGGACCCAAAGTCGATACGTCCGATTAAGCGACGTTGGTCTAGGTAACGAATTATACCCTCGGGTTACGAATTATGGCTGTGCCTAACGAATCTGGGACGTGCCTAAGTAATCTGGTCCGCGACTAAGATGTACGGTGATCGTGGACGCTTGACCGGACTTATGCGTCGCCTTCCGAGTTATTGGATGGCGTTCCGTCCTATTGGATACTATTCCGTGCGTGTGCGACACGTTCCGAGCATATGCTAACAGTTCCGTCACTATGTAACGCTTGACGTAGATTGCTATCAGGTTACGATGACTGCTAAGCCATTACGCGACATTCTGCAAAGTTACGTCGCATTCTCTCACGTTACGGCTGATTCTCTAGGCTTACGCGCATGAGCTCTAGGTTCCGGGTACTATCGAACGTGTCATTGGTACTAAAA

>67 ERCC-00040 392 744

AACTACGATCCCATGAGAACACCTGTAGATACTCAGGTCTCCGCGGACCTACGCCGCGGACGATGATAAAGTCCGAGAAACCACCGGATTGCCCCAACAGACGGCCTAGCCCATCGAACTAGGGAAATGAACTATATCGTAACCAAGCCGGGTAGCTGCGGTGGTGCTAGACTAGATGTTAGCGTTCAGTCGAGCTGTTACGTGTAACGCCATTGAGACCCTTACCCTTTACCGGTCGGCGGATACGTCCAGCTTCGTCACTGCGTTCGAGCCTTCTACACGATCCAAGTTACCAGCGCAGTTTAAGGTACGTCGCTTCGACCAGAACGAGAGTTCGCAGCAAGGGGGAGGAGTTGGATTCTTAGGGAATGAGGCTGAACCTAACTCCTCGCTACATTCCTATTGTTTTCCCGATCGGCTTCATCGGGACGCCGGAGACCGCACCTTTGCCCGTTTAAGCTCGGACGGGATGCCACGGTTCTGTTCCACAACCCGGTCGGAGCACACCCTCTCTATGCTGCGTCTATGCCTTCCGGGTGGTTGAGGTGAGCCATGTGGTCTTAGAATCCGGTTGTATTAGACAGTATTGTGCTTGACGTCGTGGTATCGGGTGGTTGTGAAGGATACAGATATTCTATGGGCAGCGATGGGGCTTCCTCAGTCCGTCTACGGCCCACCAGACAAACAACTCGGGATACAAATTGAGCACCCGCGGACCGGAAAA

>68 ERCC-00013 346 808

ACCGAGCTCAGATGTGAAGGATCTTCTTGGAGGATTAAAAAAATGATTATCTGTAAAACCCCACGTGAACTTGGTATCATGCGGGAAGCAGGGCGAATCGTGGCTTTAACTCATGAAGAGTTAAAAAAGCACATTAAACCAGGAATCTCGACAAAAGAATTGGATCAAATTGCCGAACGTTTTATTAAGAAGCAGGGTGCAATCCCATCTTTTAGGGGGTATAATGGGTTTCGCGGGAGCATTTGCGTATCAGTTAATGAAGAACTCGTTCACGGCATACCTGGCAGCAGGGTGCTGAAGGACGGTGACATCATCAGTATTGATATCGGTGCTAAATTAAATGGTTATCATGGTGACTCTGCATGGACATATCCGGTAGGAAACATCAGCGATGATGACAAAAAACTTCTGGAAGTGACAGAGGAGTCTTTATATAAAGGCTTGCAGGAAGCAAAACCAGGTGAACGTTTGTCGAATATTTCCCACGCAATACAAACGTATGTCGAAAATGAGCAGTTTTCAGTTGTTAGGGAGTATGTCGGACATGGTGTTGGTCAAGACTTGCATGAGGACCCGCAAATTCCTCATTACGGTCCGCCCAACAAAGGACCACGGCTTAAACCTGGCATGGTTCTCGCTATTGAACCTATGGTGAACGCTGGCAGCCGCTACGTGAAAACATTGGCTGATAACTGGACGGTTGTAACGGTAGATGGGAAAAAGTGTGCTCATTTTGAACATACGATTGCGATTACGGAAACGGTTTTTGAATACTGACGAGAGTAAAA

>69 ERCC-00147 373 1023

CCTTGCTTAACCTTAATCTTGCCTTATTACAATATTTTTAGAAAAATAAAAAGTAAAAATAAAAGCAAATCCTTAATCTTTGGTGATATTGATGGAGAGGTATGAAATCCCTAAAGAGATTGGAGAAATAATGTTTGGCTTGTTGTCTCCAGATTACATAAGACAGATGTCAGTTGCTAAGATAGTTACACCAGACACTTATGATGAAGATGGTTATCCAATAGATGGAGGTTTAATGGACACAAGATTGGGAGTTATAGACCCAGGTTTAGTTTGCAAAACATGTGGAGGAAGGATTGGAGAGTGTCCAGGGCATTTTGGGCATATAGAGTTGGCTAAACCAGTAATTCATATAGGATTTGCCAAAACAATATACAAGATATTGAAGGCAGTTTGCCCACACTGTGGAAGAGTAGCAATAAGTGAAACTAAGAGGAAAGAAATTTTGGAAAAGATGGAAAAATTAGAGAGAGATGGAGGAAACAAGTGGGAGGTTTGTGAAGAGGTTTATAAAGAAGCTTCAAAAGTTACAATCTGCCCACACTGTGGAGAGATAAAGTATGATATAAAGTTTGAGAAACCAACAACCTACTACAGAATTGATGGAAATGAGGAAAAAACATTAACTCCATCAGATGTTAGAGAGATTTTAGAGAAGATTCCAGATGAAGATTGTATCTTACTCGGCTTAAACCCAGAGGTTGCAAGGCCAGAGTGGATGGTTCTCACCGTTTTGCCAGTTCCACCAGTAACTGTAAGGCCATCAATTACCTTGGAAACTGGAGAGAGAAGTGAAGACGATTTAACCCACAAGTTAGTTGATATCATCAGAATCAACAATAGATTAGAGGAGAATATAGAAGGAGGAGCACCAAACTTAATTATTGAGGATTTATGGAATCTGTTGCAGTATCACGTAAATACCTACTTCGATAACGAAGCTCCAGGTATTCCACCAGCTAAGCACAGAAGTGGAAGACATTAAAAACCTTAGCTCAGAAAA

>70 ERCC-00156 242 494

CACAAGAATCCCTGCTAGCTGAAGGAGGGTCAAACTATAACACCTTTAGCATTCGTACAGGCAGGCTAAGTGAATACTAACCCACCGGCAGCCCGTTGTAGTAACGTTGACCCCTGGCTCGGAGACATTTGGTGTTGCCTAGTACTAGGTGACTGGTACCGATTCATAGGTTCGCCATTCTCTTATCGAGAGCCCGAGGTAGACTATCTTCCAGATGATGCCATACGTTCACTCAATCGCGCGGCATGCACGGTGGGGCTACGAACTTGCTATCCATAGGCTCTAGATGTGGTAGAAATATGCTGCAGGGGTTCTGTCGAATTTGCTCGGCAACCGTGGCCGTGTATGCTTTCATATCCCGGCGGTGTGATCTAGCCTTCTCGCCATATGAGGGCGCTGAGCATAGACCCAAACCCGACTAGTCGAATCTTAGGGTTGTATGCTAGAACGGCATGGTATAAGCCGTGCTCAAAA

>71 ERCC-00097 189 523

CCTTCATAATACCACCAAAATACCAATTCGAAAGTTTTAGCATATTCAAACAATATAAAATCTTCAGATTGGACATTAATTGGGACTGAAAGTCCCAACTTAATGGACGTGTGGTATCCACACCATAAAGGGGCTACGCCCCTCTTGGGATACTCCCCTAATATTGCTAATTTACACCTCCGAGCATAAGCGAGGAGGTGTTAGGTTTTGATGAACCTTTTACTAAAAGGTTCATACCAATAGGAGGTTTCCCCCTATGGTAGTTAAATGTACATTGGATATTCCTTTCTACTCTTTGACATCATCTTTAAATGTTGCTCTGCCAGTTCTTTAAGTTTGTTTTCAATAGCTTCAGCCTCTTTGATGAGATTTTCAATATTTACATTTAGATTGAACATTTTATTCAAAACCTCTAATAGATTAGCCCCCCCTCTTGGGTCTGGTCTAATTCCAACAGTTTCAGCCAACAAACCAATAGCATCAAACCCATGTCATGGCAAAAA

>72 ERCC-00164 379 1022

AGGAGCTCCAGTAGTTTTCCCCTCAAAAATTCCTGATAAGATTTCAACTTTATCCTCTTCTTTTCTTGGTGTTGAGAAGATGCTCTGCCCTGGTCTTCTCCTGTCAAGCTCTTTTTGGATATCCTCTTCAGATAAAGGCAGATTAGTTGGACATCCATCAACAACTGCTCCAACAGCCTTTCCATGACTTTCTCCAAAAACTGTAACTCTAAACATATCCCCATAGGTGTTCATTAATGTCACCAAAAATTTTTAATTGCTTAGTTTTACATTTAAAATAAAAATTAAAATAGTCAAAAAATAAAAAAGGTTTATCTGTAGAGAACATCCAAGTGTGCTGGTTCCTTAACTTTAACTTTCTTTTTCTCCATAATCTTCTCAACTGCCTTTCTAAAGTCATCCATTGTTACATAGTCCCTTAACTCCCTAATTGCATTCATCCCTGCCTCTGTGCAGATTGCCTTTAACTCAGCCCCTACACATCCTTCAGTCATCTTAGCTATTTCTTCTAAATTGACATCTTCCGCTAAATTCATCTTTCTTGTATGAATCTTCAATATCTCCAATCTACCCTTCTCATCAGGAGCTGGGACTTCTATGATTCTATCAAATCTTCCAGGTCTTAATATTGCAGGGTCTAAAATGTCAGGCCTGTTTGCGGCCCCAATTATCTTAACATCTCCCCTTGCATCGAATCCATCCATCTCTGCCAACAACTGCATTAATGTTCTCTGAACTTCCCTATCTCCACCAGTTAAAGCGTCTGTTCTCTTTGCTGCAATAGCATCAATCTCATCTATGAATATGATTGAAGGAGCTTTTTCTTTAGCCAATTTGAATATATCTTTAACTAACGGAGCCCCCTCTCCAATAAACTTCTTAACCAATTCAGAACCAACAACTCTTATAAAGGTAGCATTTGTTTCTGTAGCAACAGCTTTAGCTAATAATGTCTTTCCAGTTCCTGGTGGCCCGTAAAGAGAATACCTTTTGGTGGTTAAA

>73 ERCC-00168 350 1024

CCAATGAACTCAGCTATTCTTCTTAACAAATAACTTTCTCCAGAAAATTCAAATGTATCATCTTCAGGATTCCACCTAAAAACATCATGTAATATAATATCATCAATTTTTGGGTCGTATTCAACAATCTCAGTTATACTCTCAGTTCTTCTAACAAATCTTCCTTTATAAATCAATCTAACCTGCATACATATGGCATTTAGTTGTTCAAGCATAATCTTTGGAATGTTCATTGGTTCAGCATTCAACCTCCTTATAACTGCCTCTGGGGATTTTGCGTGTATCGTTGATAACGCCAAATGTCCTGTAGTTATTGCTTGAAATAATATCTTCGCCTCCTCACCTCTAACCTCTCCAACAATTAAATAATCTGGTCTTTGCCTTAAAGCCGCTTTTAATAAATCCATCATAGTTATTTCATATTCTTCTCCACCGAATCCACTTCTTGTAGTTCCAGCAATCCAGTTTTCATGATACAACCTAATTTCTGGAGTATCCTCAATAGATACGATTTTCATTTGAGGAAGGATGAAAAGAGAGAATGCATTTAAAAGGGTGGTTTTTCCAGTAGCTACCTCTCCAGCAACCATAATAGAATTTTTATATTCAATGAGTAACCAAAGATATGCAAGCATCTCTGGAGAAATACTCCCATATCTTATTAAATCTGTTGGCAATATAGGAGTGTGTGTGAATTTTCTTATTGTAAATGTTGAACCATATCTTGAGATATCCCTTCCAAGGGTTACATTTAGCCTGCTACCATCTGGGAGAGAACCATCCACTATTGGATTAGCCAATGTTAAAGATTTTCCACACCTTTGGGCTAAGGATATACAAAACGAGTCTAATTCTTCATCAGTTTCAAATTTTATATTTGTCTTTAAATGTTCGTATTTTCTATGAAACACATACACTGGCTTTCCAACACCTGTGCAACTGATATCCTCCAAATTCTCATCTTTCATAAGAGCATCTATTTCCCATATCCAATGAGGTAAAAA

>74 ERCC-00158 352 1027

GAGCTCTTTCAACAGATCATGGAACTACGGGAGAAATAAACATTTGGATAGAAAATGTAACATTTAAAAATGATGCAAAATCATATTCCTTTAATTTAACGAATCTTAATATATGGGCAGTAAATAAATCTGCTTATGAGTTGTATTGGAATCCATTTAACAAATCTATCTGGATAGATGGGAGTAATTACACTATAACTCCAAATATTGACATACCTCCAGGAGAAGTATGGAACTCTAAAACCTACAACTTCACATTTAGTGGAGTTCCAATCGTTTGGGCAAACTGCTCATTTACACTGTCAAAAAAAGATTATATCCTTTTAAATGAAGTAAGTCAAATAGGAAGTTCCTATGTTGTTGTTGAGGAGATTTATGTTGTGGGTAGTTATTTGATTAAGGTGACTAAGCATATTGTTCCGGATGCGGATGGGACTTATGATATTTATATAGTTGTGGAGAATATTGGTAGTGTGAAGACTCCTGAGTATGTGTATGTTTATGATTTGATTCCTAAGAACTTCACCGTCTCAGATGAGTGGGTTAATCAATCAAGTATGTTGATTGCTGAAGGAAATCACACCATTACAACAAATCCAAGGTATAATTTAAGCATGTGGTGGGCGTTACATGCAATATATCCAGGAGCAGATGGGGACGGTAACTGGAACGACACTGCTGAAATACTTGCAAATAAAACAGTAGTCATACACTACAAACTAAACGGAACTGGCGAATTCTACCCAAGCGACGCATTTATCGTTGGTATCGACCCTACAAACTCACTACTACCAACAACCTCACCAAAAATAACAACAGTAGCCGGAACAGTAGAAAACAACTTTGAGATATTTTTAATACTGATAAACGTTATCTTTGGATTAGGTATATTAACAAAGAGGAATATAAGGAATAATAAATAAAGTTGGGGGATGATGATGAAGAGATTTGCATTACTCTTTATTTTCCTTGCATTTATAACACCACTTTTGCAGATGTTGAGGAAA

>75 ERCC-00142 246 493

CACCTGTCACATTTCCAATCGGCTCCAGGAAGAGAGAAGTGACGGCTTGATCCTGTAGTAATCCGGGATCGACTTAAGGGGTGCAGCGACCACGGCGGATCGGGCGTCGCAATAGTCCTCCTGTTAGGAGGGTCCTTCTAATGTTAACGCCCGAATATTAGTCATATTTTGCTAGCGCCTATCAGCGTAAGATATGATTTAAGTTACACCAGGAGAGTAGCGAGATAGAACCACTCGTTGGATCGGTCTTTCTTAATTGACTACTATCAGATCCGGCGCATGGCGCTGAGGTCAAACTACATTACAGGCCCTGGTTTCCATGGGTCAGCGCAAGTACAGGCGAGCAGATACAACCTTCCGGAGACTTCGCCTCCACACACCGGAGACCCTAACCGTACCCAAATGTAACTAGCGCCTCTGGTGTGAGCTTACTAGAAAGTAGGCCGGGCCGGTCGACAGGAGGTTGCGCCAAA

>76 ERCC-00081 260 534

TTTATGCGGGGTGTTGTTGATAAGACTTCCTAGGCACGCGGACCAAGCACGTTAAAGGGAGGTTCGCTCGATCAAGAGATCCATACAGCAGGCTCCTATCAGTCTATCTGACGCCAGTTGCCATTGGACGGGATGTCGAGGGTTTGCGGTGGGGAACGACCATACTTACTGTCATTACGACGTGCGGGGAAATTGAGGTTAAGTTGTTTGCCACCCTACAATAACGAAGAATTTAGCCACCAGTTGGCGTTAGAGTTGTCAAGACGTTGGCGTGATGGGATGAACGTCGCTTAAACGTTGGTTCAGGGCTCCATATATACATCTGCCGTGCCCCCCTTAATCTCCATACAGACCCCCCAGGCGGAATGATAAGTCGATACCAGGACAATGACGGCTTCAACGTTTTCACTTTCCGCAGGTGGCGACCCTCTAGTTGCACGCTGGTACATCAGACACGTACACACGCGCAGTGGGGTGTAATCAAGCAGGACCATAACTGATAGTTCACCAAAAA

>77 ERCC-00024 248 536

ACGGAGGAGCTTTGGCATACTAGGCTAGCGAATCTGCAACTAACGCAAGTTACATCCTAGCTAGCGAAGGGCGTCCCAATTTTCGCTAACCCGACGCGACGCATAAAAAGCGAGAATAACGCCTAAGGGATGTACAATGGATGTTGATTATGCCTTCGGGAATGAGGGATGATTTGCGAAAAACAAGTCAATACCTAACCAAATCCGCTAATGGACACCCGTAATCGTGCCCAAGTTTAACTGGTCGGTAGGTGGCAGGCAAAGCGCTAGTATCCCTAGGCGCGACACTATAAGTTTACAACTGCGAGAATTGACACTATGAGCGCGCATACTGGGGCCAGAATAGGCAATACCATGTGCGTCCCTGTGTGAACAGCTCGCGGCCATCAGAAGTTGGGATTGACGCATGATCTTGATCGAGCATACGGCTTCCACCAACCCATAGTACTTGGTAACTATAGCAATCAAGCACGCGTGAGCACAACGCTATCCAAATTACTACATTAACTGGAAAAA

>78 ERCC-00016 409 844

GTTGTAACGAATGTTAATTTAGGAGGCAAGAGTTTGTGGGCGCGGACTTGCAGCTCGTAACGCTCTAAAAGGGTTATGCCGCTGAGGCGGGACCATAGTCAGGAAGTTTGTCCGATCCGCTCCAGTTGTCAAGAGTAGAGGATTTCGTGTTCGCCGATACTGCCGAAAACGTCATACCGAAGCAATTCTGTCGTCACTCTGTATGTCCGTGCCCCACCTTCGAGTATGAGTTTTAAAGTTCGTGCAGAGACATAGCTCGCGCACTCCCTGTGTGATGCCGGTCGGCCGACACATGCTTCAATGTGCCTTGAACTTGCATTCGAAAGAATGTCCTTATCTTGATCGGCCATTGTAATGCAACGCTCTCCTTTCATTGACACGAGGTTCGTAGATGGCTGTTACTCGCGGACGTTAAATAAAACTATCAGCGTCAGCGGATTAGGAGGCTTACGGGGGAACCTACAATTGTTCGCCGCATGGTCCGAAGGCGCCATGTCCCTCAGCGGAGCGACAACAATTACTACTGGAGCTATTGTAAATACGCAGCAACAAGCGTCCAGACATTTGCCGCTGACCTCCAGTCGCATGGACGGGGGAGAACAGCTGGAGCAATGCATCATTCGCTGAGGGACATCCAATCACGGAATCAAGGAAGAATTACCAATTTTACCTGTAACGAAACCAATTTATTACTGACGCAGAGTGAATCACATCTATAGCGGTTACGACCCTCCAGAGTGATCCGACGGCGATGTGTCTTTGACCCCCGTGTGACTGGTGTTCCTGTCGTATCCGCAACATGTTTACTCATCACTACGTTAAAA

>79 ERCC-00123 364 1022

TTAAGAGAGGAATAATTACTTTTTCATCTTACCCACCAAGTTTTAGTTTTCTTATTCTTTCACTTAATTTTTCGACCTCCTCCTTTGTTAATCTTGTAGCTTTTTTAACCTCCTCCAATTCTTTTTGTAACATTATTACTTTAATCCCCAGTATTATGTTGAGGAGTATGCTAATAGCCACAATTATATACAAAATCATCCTTATCTCCTCCCGAACAATCCTTTAATAAACTTAGATATGAATGATTCTTTCTTCTTCTCAAGTTGTGCTTCATATTTAGCTCCAATTAACTTAGCTGCTATCTCCATGACTGCTTGAGCGGCTGGAGAATCTGGATACATAATAACGAGAGGTGTTCCAAATGCAGCTGCCTTCCTAACATGAGGGTCCTCTGGAACAACACCTATAACAGGAACTTCTAAAATTGTCTCTATAGCTTTAACCCCCAACTCTGTACTCTCATTTGAAACCCTATTAACAATAGCCCCAATGATGTCAGTTCCCAATCTTTTTGTTATAGCGATAATTTTTAATGCATCTGATATTGGGGATATCTCTGGATTTACAACGACAATTAAACCATCTGCTGATGATATTGCTATTAAAGTCTCTTTTCCAATACCTGCTGGACAGTCAATAATTAAAATCTCAACTAAATCATGTATTGCCTTTAAAACTTCCTCAAGTTTTTCTGGTTTAGCTCTTCTGAACTTTTCTAATGAAACACCTGCTGGAATAACTAAAACTCCTTCAGGACCTTCATAAATTGCGTCCTTTATATCTGCTTTACCAGCCAACACATCGTTTAAGGTTACTGGCTTTCCTTCTAACCCCATGATAGGCTCTAAGTTTGCCATTGCTATATCAGCGTCCAAAACAGCCACTTTTTTTCCAAATTTTGCAAGAGCCACAGCAAGATTTGCAGATATCGTTGTCTTTCCAGTACCTCCTTTTCCAGATGCTATCGCGATAGCTATTCCATTAATGTCACCATTCTAAAA

>80 ERCC-00041 509 1122

CCGAGCTCAAGATGCGTTAATTATGTGGGTGACGATAAATGAGTGAGCAAAAAGACATGTACGTATTAGGAATTGAAACAAGCTGTGATGAGACTGCTGCAGCTATTGTGAAAAGCGGGAAAGAGATCATTTCAAACGTAGTAGCCTCTCAAATTGAAAGCCATAAGCGCTTCGGAGGCGTTGTTCCGGAAATTGCTTCAAGACATCATGTTGAACAAATCACTTTGGTTATAGAAGAGGCGTTTCGCAAAGCTGGCATGACGTATAGTGATATTGATGCGATTGCAGTAACAGAAGGTCCGGGACTGGTGGGAGCGCTTCTTATCGGAGTGAATGCCGCTAAAGCATTGAGCTTTGCATATAACATTCCGTTAGTAGGCGTTCATCATATAGCCGGTCATATATACGCGAACCGTCTTGTAGAAGACATCGTGTTCCCGGCACTGGCATTGGTCGTTTCAGGAGGCCATACAGAACTGGTTTATATGAAGGAACATGGATCATTTGAAGTCATTGGGGAAACCCTTGATGATGCGGCAGGAGAAGCCTACGACAAAGTGGCGCGGACGATGGGATTGCCATATCCGGGTGGACCGCAAATTGACAAGCTAGCTGAAAAAGGGAATGACAATATTCCGCTTCCTCGCGCATGGCTTGAAGAAGGCTCTTACAACTTCAGCTTTAGCGGATTGAAGTCTGCGGTGATCAATACGCTTCATAATGCATCCCAAAAAGGGCAAGAGATTGCTCCGGAAGATTTGTCTGCCAGTTTCCAAAATAGTGTGATCGATGTCTTGGTAACCAAAACGGCGCGCGCGGCAAAGGAATATGATGTCAAACAGGTCCTTTTAGCCGGAGGAGTAGCTGCAAACAGAGGCCTCAGAGCTGCATTAGAAAAGGAATTTGCCCAGCATGAAGGGATTACGCTTGTCATTCCTCCATTAGCTTTATGCACGGATAATGCTGCGATGATTGCTGCTGCTGGTACAATTGCTTTTGAAAAAGGAATTCGCGGTGCATATGATATGAATGGCCAGCCCGGCCTTGAATTGACTTCTTATCAAAGTCTCACGAGATAATAGCGTGAGACTCCCGGGTACAA

>81 ERCC-00104 662 2022

TGCAATCTCTTCAACAGCCTCTGCTAAGTATTCTGCAGCTGCTCTGCTAACTCTCTCAGCACCAGCCTTTTTCAATATTCTCTCAAATGGTGCAACTGGAAGCTCAGCCATAATACCACCTCACAATAGATTTCCAATAAATACTGTTATAAAATCCTTTATTTAAACTTTTCGGTCATTTTCATTTTTTGTGAAAGTCTTTGGAAAATTTTCCCACACATAAAGAAGGTATTAAAAAGTGTGACACTAAAATTATAAAAAACACTATTTATAATGTATGTCACAAATTTAAAATATAATTTTATGAAAGATAGATAAACATAAATTGGTAGAGTTTAATTGTGATATTTATGATAATTACTATAGCTTCGGGTAAAGGAGGGGTTGGAAAAACTACAACATCAGCATCTTTAGCAGTAGCACTTGCTAAATTGGGAAAAAAGGTTTTAGCTATTGATGGAGACATATCAATGGCTAATTTAGGGATTCTATTCAATATGGAAAAGAAAAAACCCTCTTTACATGAAGTTTTGAGTGAAGAGGCAGATGTTAGGGATGCAATTTACAAACATAAAACTGGAGTTTATGTATTGCCAACGAGTTTGTCTTTAGAAGGTTATAAGAAATCAGATATTGATTTACTTCCAGATGTGGTTAATGAGGTAGCTGATGATTTTGATTATGTAATTATAGATGCTCCAGCTGGGTTAAATAGAGAAATGGCTACTCATTTAGCTATTGCTGATAAACTTTTACTTGTTGTCACCCCAGAGATGTTCTCAATTATTGACGCTGTTAGATTAAAAGAAAGTGCTGAAATGGCTGGAACACCTTTAATGGGTGTTGTGTTAAATAGGGTTGGTAGAGATTTTGGTGAAATGGGTAGAGATGAGATTGAAATGTTAATAAAAGGTAAAGTTTTAGTTGAAGTCCCTGAAGATGAAAATGTTAGGTCAGCAGCTTTAAAAAAGATGAGTGTTATTGAATATAGAAAGAATTCTCCAGCTTCTCAAGCTTATATGAAGTTAGCTTCAATAATAGCAGGAGTTCCTATTTACATTGAAGATGAAATTAAAATAATAAGGAAAGAAAGCTTTATAGATAAAATTAAGAGATTATTTAGGATGTATTAATTATCTTGATTTAAAAATTTTAATTATCATCCTTTTCCAAATAAACCGTATATGTTGGGAACGCCCTCTCAATGAACCTTTCAGTAAAAGCTTCACCAAAAATGGATGCATCATCTCGCTTTGCTCGATGATGCCTCTTAGTTATCTTTCTCCAAATAAACAGTATATGTTGGGAATGCCATCTCTATCCCTTCTTTTTCAAATTCCTCTTTTATCTTCAAATTTATTTCATCAACGGCATTTAAATAGTAATCAAATCCCATGTTTCTAACAAAGTATTCTACCCTCAAATTTAAACTCCAATCTCCATATTCCCTAAAATGCACTCTATATGGAGGGAGAGTAGCTGGATGATTTTCAACAATCTCTTTTATTATCTCCTTAGCCCTCTTAATTTTCTCTACCGGTGTGTTATAAGTTAAACCGATAGTCATTAAAACCCTTCTTCTATCTCTAACTGTTAAGTTTTCAATGGCTGAATCCAACAATTCTGAGTTTGGGATAGTTATTAAAGTGTAATCAAAAGTTCTAATTCGTGTGCTTCTTATTCCAATCTCCTCTACAATCCCTTCAGCCCCTTTAACTTTAACCCAATGGCCTAAACTAAAGGGTTTGTCAATCAATATTAAAATCCCAGCAATGAAGTTTTTTATGGTGTCTTGCATAGCCAAAGCTAAAGCTAAACCCCCTACTCCTAAACCAGCCAATAAAGCAGTGATATCATAACCAACAGAGCTTAAAGCCGTTAATATACCAAGAAGTATTGTTAATATCTTTACAACTTTTTTCAATGGCTTTATTATGTGTTCGTCCAACTCTGTTTCTGTCTTTTCGGTCAATGGAATTAGGTAGTGTTCAAATATCCCAAG

>82 ERCC-00012 506 994

CGAGAGATGTTTGTAGGTGCGGAATGTGTGCGGTCTACCTTAGCTGTAGTGTGCGATGAACCTACACACAACGTGGTATAGTGGCCGATCTTAGAGTGATCCTATCACTCCTTACGCACCAGAAGGGATCTGCATACCAGGCGGAGAACTTGGAAGGCGGCTAGATCACTGAATTGCGGGAATCGGCATTTCGCATTCTTAGGATCTAAACCTTAGACCTCCGCGTGCGATTGCACCTGCTTGGTACAGAGTTACAAGCCCCCCGCACTTTCTTTGCGGTCGTTAAGAGGGAAATCGCCCAATTAGCAGAGTGTCAGGTGTTACGCGCGATTGAGCCGTCAGAAGAATCGATAGAGCCGCGTCGGGACCTTGATGGTATCTCTGCCTCAGCTAACCTGCTAGGTCCGTCCCCTGGGGATGATCAGGACTGCGGATAGTAAATTGCGGGTTTGAAGCCGGACTTGCCGCCTAGGCAAAGCACAAAAACATCGGACATGTAGAAGTCTCATCGAACTCCTTTCCCGTTCATGCAGATACTTCAACTGTGACTAGTGGGGTTCGGGAGCACCCGCACTACTTCATTCTTGGCGGTGGGCCACTTTATGTGACTGTACATGGGACTTCTACTCATACCAATGTAAAGTATAGTTAACGCCCTGTCCACTCTACTCAGGCGTAATCATCGCGGAAGGCTATCCACAGCCCATCAGCGGTCTACATGTCCCAGCAGATTCACCTGTCCTGCGGGTCCGCGTCACAGCCTATTCTGAGGCTCTAAAGACTATGCGAACCAGGTGTCCCAGTCGATCAGACGACGAAGTCGGGAAGGAAGCATGGATACCAAAAAGGCTTTATATACTGGGTTATCCTAGGGGATGTTTTTACCGGACTGGTCAGCCTCGGTGCGCTCGGCCTAGGCGCTTACTGCATGGGGGCTGTGGGCAATTTGGTATTTCTCAGGACTATGGACAAAA

>83 ERCC-00086 329 1020

TTTCTCTTGACTTTTTCCATAACTTCCCTAATTGTTCTATAATTCTCTTTCAAAGAGTCCTTATCATAACCAAATACATAAACTCTAACTCTATTTCCTTTTGATTCTATTGTGCAATCAATGTCCATCCTTGATAATCTCTCACAAAGCTCCAAAAGCTCTTCATCACAACTCACTTTTGATGAAATAATCTTTCTCATAGTATCGCCAAAATAATAAAGTAAATTTACAAATTACCATAGCTTATATAATAAAGTTTTGCATGAACAAAAATGTTGTGGTGATATATCATGGACGAATTTGAAATGATAAAGAGAAACACATCTGAAATTATCAGCGAGGAAGAGTTAAGAGAGGTTTTAAAAAAAGATGAAAAATCTGCTTACATAGGTTTTGAACCAAGTGGTAAAATACATTTAGGGCATTATCTCCAAATAAAAAAGATGATTGATTTACAAAATGCTGGATTTGATATAATTATATTGTTGGCTGATTTACACGCCTATTTAAACCAGAAAGGAGAGTTGGATGAGATTAGAAAAATAGGAGATTATAACAAAAAAGTTTTTGAAGCAATGGGGTTAAAGGCAAAATATGTTTATGGAAGTGAATTCCAGCTTGATAAGGATTATACACTGAATGTCTATAGATTGGCTTTAAAAACTACCTTAAAAAGAGCAAGAAGGAGTATGGAACTTATAGCAAGAGAGGATGAAAATCCAAAGGTTGCTGAAGTTATCTATCCAATAATGCAGGTTAATGATATTCATTATTTAGGCGTTGATGTTGCAGTTGGAGGGATGGAGCAGAGAAAAATACACATGTTAGCAAGGGAGCTTTTACCAAAAAAGGTTGTTTGTATTCACAACCCTGTCTTAACGGGTTTGGATGGAGAAGGAAAGATGAGTTCTTCAAAAGGGAATTTTATAGCTGTTGATGACTCTCCAGAAGAGATTAGGGCTAAGATAAAGAAAGCATACGCCCAGCTGGAGTTGTTGAA

>84 ERCC-00138 338 1024

CCCTGCTAAAAGAGGTTGGTTGTATAAGATTTGGAGAATTTATCTTAGCCTCTGGTAAAAAAAGTAACTACTACATAGACATAAAAAAAGCCACCACAAACCCAGAAATTTTAAAGTTAGTTGGAGAAATTATTGCTGAGCAAATAAAGGATGAAGATGTAAAAGTTGCTGGAGTAGAGCTTGGTTCTGTCCCTATAGCTACAGCTGTCTCAATTATTGCTCAAAAACCACTATTAATTGTTAGAAAGAAACCTAAGGATTACGGAACTAAAAATAAGATAGAAGGAGAGCTAAAAGAAGGAGATAAGGTTGTTATTGTGGAGGATGTTACTACAACTGGAGGAAGTGTGCTAAAGGCAGTTAAAGAGATTAGGGAAAATGGTGGAATTGTTGATAAAGTTTTYGTTGTTGTTGATAGGTTAGAAGGAGCTAAAGAAAACCTACAAAAAGAGAATGTTGAATTAATCCCATTAGTTACTGTTAAGGAGCTACAATCCACTCAATAAATCTAAAAACCTCTTAGTCCATAGGGGAAACCCCTATTGGGATACTCCCCGTCCATTAAGTTGCTCCTTTCAGGAGCAATTAATGTCCATTTTAAGCTTATAATCCACTCAATAAATCTAAAAACTTCTTAGTTTTCTCTTTTTTAACCTTACCAAATTTCTCTAATTTGCTAACATCTACATTTTTATCCTTTAAAATTTCTTTTATTGGTTCTATGAGTGCAATTTTTTTAAGTAAGTCTTCAATATCCTTCTTTTTGAAGTTAAATCTTTCCACAGCATCCCAGTCGTGAATTAAGTATAAAGCAACTCCACCAATGTTCTTTAAAGGAATCTGATATTTTGGCAACTCCTCCTCCAATTTAAATTTTAAATCTAAAACATCCACACAATTAATCCCATCAACACTCATCCAAGATAAAAATCCTCTCTGTGGAGTAATCATAATAGATGGGAACAAATTACTCCTTGCCCTTCATCTGGTGTTTTGTAGAAAAA

>85 ERCC-00017 584 1136

GAGAACTGAAAGTGAGTCCCAACGAGAGAGGTGCATCTGTCCAGTGAGAGCTGACTGTCTGCGACAACACTAGTCGGTCCAGGCATGGATTTCGCGACCTCACAACTTAAGGAGGCGGTAAATCAGATGACAGCGCGACCCTGTAATGGGTGACCTGCTAGTGGAGGTGGCGCGGTGTCCCAGATACAAGGATCTCGATGTAGTACCCTCACATAACTTTGCTCCCTGAAATAACATTCGATCACTCTAATGAATCCCTTAAGCCAGGAGCGTTAGTGTCAAACGCAACGCCCCGGGTTCATGATCCTGGATGGCTGGTCGAACCAGGGAGATGTCACTCTAATAGGTGCCAAATGTACCGCAGAACCTCGTAGGCGTTCGCCCAATTGGACCCGAGGTATAATGTAGACGGGCACGCTGACTGGGCAAAAGATTACAATCCCAGTTACCATACAGTCGCCCGGTCAGGATCGGGGCATGAAGGCAATATGTTGGCGCATCCCAGTCTTTCCGTAGAAACAGTGGCTAACGACGGAGATACTGCCGGGCAAGAAACCTTGACCAAGTATGCGCGCCTTGTGAGTCTCCATGGACTTGCTGCACCTACAAATCCGGAAGGGCGCTTATAGTGCTTGCTAGCACTCCCTGGAATATCTTAATCCCGCCAGCTCATGGGACGGGAGGAATGTGTTAGACCATAAACAGAGGGCTGGCCAACAATCAGAGGGAAGTAAGCCCCGCAAAAGGATTCTGCGGGAACCGAATTACACAACGTAAGGACGTACCTGCTCCTACCCCCGAACCACTGTCAATACGATAATGCGCCCAAAACGAGGGATCGAGACGGTCGGAGTGGCAGTCCAGCTTAAACAGGTGCTCGCCGAAACTAGCTGGCCAGGGTGAGGCATGGATTCAAAGCCAATGACCGAAGAAAGTTCCGACATACAATTACTCGGCTTTTGGCAATACCCAGGCGAGAGGTAAGCCCAAGCCATACCGGGAATGACCTGAGATCCACTTAGTAAGTCTTACGAGATGATCCCGACCCAGGACTGGAGCTAGGCGGTTGCGCAAGTAACTTCATCATGTATCGCTGGGGAATAATGTTCCTGGAAA

>86 ERCC-00061 569 1136

GGAGAAGCTTGGCAAACACTACCGGGCATAGATAAAGACGCGGGGCCAAGCATGCCGACATTGGGATACTTCCATGTTACGGGGCATGGAGGCGCAGCTATCCCACCCTCGTGCGCAAGATGGACAACCGAGCGCGGTAGTAATCGCGAACTGCCAGGCACTTATTGTAGAGGTGAATCGCTAACTAACTCGGTGTAAGTTTCCGTCGATGAACAGGCATCGGAGAGGGCTCTACGGGCCACCCAGTAGTGACGTCAGGAGTGCATAAGGAAGCAGATTGACCGTGGCGCAGACTCGCGATATGAATAAGACTCTAGGGGGTGTACAATTGATCGTTGCGTACGGAATCGTGCGACCTCAAAAGTTAAGCGCGAGGGTCACCTTGCTCCGCGCGAGCAGCCTTTGTCCAGGCCTAGTCCTTGCATTCGACCTATCGCACCGAGCGTCTCACCCAGCTAGTACGAAATTAACTGATAGAGATGGTTAACCCCCTCAGAGCGACTGGTTTAAGTCTAGGACCGGACAAACGGTACGATTACGTCTTAGCATCAAGTGGTGCCTGATCTCGTGATAGACAACGTGAGAGTATTTGGATGTATGGCTAGTTACGCAGAGCATGTGGCATTATTCTCATGTTTTGCGGCGGAGCGACTCAATTTATCCGAACCATGGGAGATCGTCATTCTTGTGGTGCAAAAAAATCACGGGCCCTGATACTGGTCGAAGTTGCGCCCTATGCTTACACGGCGCACGCCGCGGTGGAGCGATCGAGACCTCTCGGATCTGAAATAAACCCGCAACGTGAGGTAGTGTTACGCATACATTCGCCCACGCTAACTGATGCGTTGTATTTCTCGGAGTCTTTACCATGAGAATTGGCATATGGAAATCCTGTCATACCACGGGTCGATACTTGTTCTCCGTCTGAAGCGAACAACGAAGCATGTTGACCGTCTAAGATGATTTTTCCGACAGAGTGACGCATATAACACCTCTGGTCTTACAATGATTCGAAGACACGTGAGACGCACTAGAGGCTTAACCTGGCAGATTGTGATCTCCGATGGTAATGAAGTCGCCGTACTATCTCTTACTTGCATGACGCGACTACCAAAA

>87 ERCC-00117 581 1136

AGATACTATCAGGCGCCTATGGGCAAATCTGGCCTCCAAACTCCGAGTGTCACCGAACCGTAGGAAATTCCTTCCCCAAGCGTGTGCAATACTCGTAGCACACCGGGATAAATAGGAAGGAAACATCCGCGGGTCTTACGGTGAAGCTGTGGGACCGCTTCTGTACACGGCGTCCAATCAAGGGGCTTGGCATTTGGCCCAGATCGAAAGTGTTGAACCACAAACGACTCTATCGGTGACGCCTCCAAGCGCGAAACTCCAGGCAATGGGGAACCGCAGAAAGATGTTCCGCTAGACCGCATTAATACCACATCTGGGCTTAGAAGCCCCTCGGTGCTCCAGGGAGGACGGATCGGAATTAAACGTGATATGGAACTTTAACTGATCAGGCCTTGCCCTTACTAATGGCGCGTTGTAACGGGCCTTGAGGGAATGTCACTATTGAGGCACCCGTTCGACCCTCAGAGATATACCATTCCGCCTATTGTAGCTCTCCTCGTACCACAGTCTTGCAAATACTGTCATAGCTATGGAACCGCCCCGACGCCGGGATTATGGCCTCTCATGGACTCAGTGTGATCAGAACCTGCTCGAGTGGGGACTGGATGCAGAGCTGATCTCTGTAGTGTGTTGTGTCGCGGGAGCACCACCGGATGACAACCGCCATATTCCAGTGCCCAAATACTCACTGGTAACGGCTTGAACTCATGCTAATTCCATTATAGTTCTTTAAACATAAGCTTTGCCCTCGGGGCCCATCCCCTGAACCATGCGGTGAGTCACGTACGCAGACCTGAAAATTAAAGATCCGGACAGGCCCGACCTTAGCCAAGGGTAAGAACCGCTTCACTTAGTGAACATCCTATCCAGTCCGTGCAGCATCGTCTACGGTCCGGGCTTCTGCCGAAAGGTCCATTAAACAGACAGAAGGATAAATGGCTCCAGCGGATACGCGCATATTCGGTTATCGCAGTTACCGTGCAAAACTGCATCCCCGCTGGGATAAGCACATGAGATGGACAAGGCTTCCTATGAGTGATTCCAAAGAACATTGCCCGACGACCGAGCCTTCAGGTAATCCACGAGACATATGCTAACCAGTGGTGCCATGGAAAA

>88 ERCC-00098 588 1143

GGAGGTCGCAATTACATCGGTTCCTGTCCCGTAGAGGCTGGAAGGGGCATAAGAAGCAGTGATACCAACGCTCTCCCCCGCGCGTCTCGTGAGAGCAGACCATGAACATCGCAGAGGAGAATCCTGCATGACTGAATGCGCAGAGCAACTGTCACCACGTGGTTAATGAGAAGGCAGAACTCAACAGACAGCTCTGGATCTGCTGCATCCCAGGGCAAGAATCAGGAAAGCAGATGCAGTAACCATAGGCACGTGAAAATGCTCCCGGCCACACTTTGGAGCTATTACCATGGTCGGGCCCAAACATAAGTGGACAGCTAGAACGATTTCTCAAGCCTGGCAACGTGGGTTAGAACTCCAACCCCTCGCACGTAGTATGGCGCTGGAGTAAAGAGCGCTCTGTTAGCAAGCGACCAGTCTCCCGAAAGTACAGGATGTGCATGTTCTAACCAAAGGGTCGCAGGGACGATGATTGCTAGTAGCTTGACAAGGCTATCCTAGTCATCCTGAATCCGGCCTATCAAAGGAATGCGTGGCAGGTCAAGTGACGAGAGTGGAAGAGCTTCCCGTTGACAAGCGGCAAGTAGACTGTGCCTACCCGGGCTTTCCAGACCTAAGATATCTGCATTCAGCAGAGTGTTGTGTTCGGGGCAGCAGTGTGCCTTCATTCGTCAACTGGAGCCTAAGGCCCCAAACTCGATCATTGATGACTACTCGACAAAAGAGGGTGGTATACAGAAAGAGGCTTGTGTCGCCGGAAACGCTTATCCGCACAGTAAAACCTCCCCAGATGACCTTCTCCCTCATAATCACTTAATCTGAGCGCAGGAGGCAGGCTGTATTAATTCCGGCCTCCAACCGGACCGTGGAACGACGCGACCAAGTGGTCGACGGGACATGCCCAGTATTTGGCCGTTCTGCCGATTCTCAGCTAGCAAACCAAGATCGTACTACGTACGCGCCTGGATAGATCGACGGCTGTTTAATAAGAGTCACTCCAGGCCTGTGCTAGGATCAGGGCGACCATGCCAAATATCAACTCAAGGACAAGTTGACGCCTGCCTTCTGGGGTATGGATCAAAAGCCCACGTTACCATGTAAGACCGTGTGGATTTTCGAAAAA

>89 ERCC-00083 353 1022

GAGTTTATAGTCTTTTTTTGATTGCCTCAACTAATGCCTCCTTTGTAGGAGCTCCAATAAACTCAACATCCCCATTTATTACAATTGTTGGAACTGCCATTATCCCATATTCCATTGCCTTTTGAGGATTCTCCATAACGTTTATGTATTCTACTTCAACAGCATCCGGCATTTCATTTGCTACCTCTTCAACAACTCTTTTAGCTGCAGGACAGTGAGGACACATTGGTGATGTAAAAAGCTCTATCTTTACCTTTGACATACTAACACCTTAAAAACCTCTTAAGTTTATAATAAAAATTTTATCCAAATTTTTATAAATAGTTTTCCAAAAATAGAATATTAAATTATAGGCTAAAAATTAATGACCACATGTCCAAACTTGTCTGAAGTGTTCAATTGCTTCAACAATATCCTTCAATTTCTCTGGTGGGAAAGCTACAACAACTTCTTCTGGCTTAATTCCAGCGTATTTTCTTGAACCGTTACAACCTAAAGTCATGTTAGGAGCTTTTCTTGTATAAACTGCCGCTACAGCATCAGCACACAATGACTGAATTCCTGAGAAATCTGCCTGGAATCTTCCACCTTTATGGTAGAGTATTGCTTGAACTAACCTCAACGCATATAATGGCTCTCCAATAAATACAATTGAGTCTGGAATGAAGTCGGTTTCATCTAATGGAGCATAGACTGTTGCATAAATTTCCTCTTCAACTTTTGGTATTGCATCAACTGTTTTTTTAGCTGCCTCTTCATCTTTAAAGTTTCCTAATTTGACATATAATTTTCCTGTTGCTAATGGTTCTGGTGGGTTTCTAAAGACCCCCATTGCATAAGCTCCTCCCTTACAGAGGTGTTTATCAACTGTTGCATATAATTTTTTTCTTTCTAATCTTGCCATTTAAATCATTTCACAGTGTCTTTTTTCTTCGTCTAATGTTTCATAGCCTTCTGGAATTTCTTCCTTTGATTTTGCCATTTTACAGCGACAAATGGAAA

>90 ERCC-00048 473 992

TCTGTAAATCCCGTAAACGAGTAGTACGAATCCGGACTTGAATACACGCGTCAATCCCTTTTATATCCTAGAATGGACCGTGTGGACGGCAACTCAGAGATAACGCATATCTATGTGCTCGCTTGCCCATCAAAGAAGAGACGGCGACCAAACGGACGACATATAGTGACATGGTCAACCCGTACGCCTGCTTCGTAAGCCGACGGTCCTTTGAAGAGGCTGGCGAATCATGTCGTTTGTGCTTACTATTACATGCTAGCTTGGTTGGGGCATCTCGGGACAACGTCTATGTACAATAAACACAAAGCCGCGTAGTTATCTTCCGCGAGTTCCGCCCAATACATTGGCGGTGACTTGAGACCGCTAAAATGCACATAGAAGCCTCAAACATGGTAAGACTATAGATAAGCGGCGCGAAAACACGGCATTTGGAATGATGTGTACTGGGAATAAGACGACGTCGCTATGGCCTCTCCGGAAGGCGGTGTATGTGCCAAGCGATGTTTCATTAATGTAACGGACAGGTCGCTGAGGTGGCTTTCGTTGGGGGCGCCGTCTTTGGGGGAGATTGCGTCAATTTTGACTGTCAGATCAGCGACTAGATTTTAGGCAGATTAGTGTGCCACCTGAATCAATAGAACAATATCAGTTATGGCGGTGCAGTATACTATACAATGGGTTGGGCGCATCTGCATGTCTCATGCTGTCATGGCAATCGACCTCTAGTCTGGGGTGATCCGAGGCGCTTCTCTTATTAGGAATAGTGCAGGACCCGAAACCGCCATAGGGAAAGGGTGAGCGAGGTAGCAGCGTAATAATTCGCGGTGGGCAGGAAATGCTTAGTGTTCTGTCTCAAGACCTAAGCGACAGCGTGACCTTGTTTCACTTACCTCTGAAGCTCTTCGACGTTATAGATATTGGCATCCCTAAACAACGAGTACCTTGTGCTACGACAGAAAAGTGACCTGAAAA

>91 ERCC-00057 508 1021

CTAGTGCATCCTCGTGGCATCATGCGTCTCCTCAGTAGGTCTGCGACTGATCCTAGTGCAATGCGTCTGAGCCTGAGCTACAGCGATATAGCCTGGATTGTGAGCGTATTTGCTGTCAGAACCTCAGCTCATCATGTATGATGCTGTACCATCCTGCGATACTGAAGATGCACCGCTATAATGCGAGGCTCTCCGCTAAAGTGGAAGCTGCTCGTTCTCAATGCGAGCGAGTCGAATCCAATGCCGTAGCTGCGATAACGATGCCGCTGACTCTACGGTAATGCACGATCCTCTACATTGATAGCAGATAGTCTAACGGGATAGCATAAGTGCAAGGCTCCTAGCATGTAGTCACAGGTGCTCAGATATAGTCATCGCTGCAATCAGCTAGTCATCTTGTCAGGATGCTACTCACTGCGTGCAGAAGATTCGCACGACTTCAGAGGATGGCACTCGTCATTAGAGTGATGTTCTCGGATCGACACTGCTGGTCTGCGAATGACTCGCATTCACTAACATGGAGCATCGTTATCTAAAGGGGATGCACGTTATCGTCGAGTGGCCGTCATGTCTATGCAGTGCGGCCTATGTCTCATTAGCGAGTCGTATGTATCATGTCGGGCTCGAATGTTGCACACGTCTGCGTAATGGTGACCGCTAGTCCCACATGGTGCTTCGTAGCCACAAATGTCGTTAGGTAGACCGACGTTATCGCGCTATACCCGATGTCAACGCGAGTTAGACCGTATCGTCCCCAGTGCCCTAAGATGGTCAAGCGTGCTCCTACGTTAGTATCAGTTTCCCTATTGGTACGTCTGGCGTACTTCTGAAACGTGATGGGCGGCTGGTTACCCGTATATGGGCTCGGTTGACCTCTATTGGGCGTTGTTGACCCGAATTCGGTATCCTCGTCGTTAAATGGCGAACGTCGTCTGCTATAGGCAAACGTCTGTCGGTCATGGCAAATGTTACTCGTGTGTGCAAGAAATTACTCGCTGTCA

>92 ERCC-00075 371 1023

TCCTACACGTTCCCTCATCTGAAACCATACAACTACCAACTGGATTTAATGGAGTGCAAACCGTTCCAAACAATGGGCAGTCAGTTGGCAGTTTCTCTCCTCTCAAAATCTTATCACATATACAACCTTTAGGAATTTTCTCTTTAATCTCTGGAATATCCTCATGCTCATAGATGTCAAATTTCTTATACTTCTCCCTCAATCCAAAACCACCATTTTTAACAACTGGGAAACCTCTCCAAGGAACATCTATGCTTTCAAAAACTTCATTTATTATTTTTTGAGCTAAAACATTACCTTCTGGCTTAACTGCTCTAATATATTCATTTTCAACCTTTGCCTCTCCACTGATGACTTGCTTTAAAATCATTATTATAGCCATTAACACATCTATTGGCTCAAAGCCAGCAACAACCATTGGAGCTTTGTATTTTTCACACAACCCATAATAAGGCTTTAATCCGGTGATTGTTGAAACATGTCCTGGGCATATAAATGCATCTAAATAAACTCCCTCATTTAACAAGAACTCCATAACTGGAGGAGTCTGCCTGTGGCAATTTAGGATAAAGAAGTTATTAACATCTTTATTTTTTAAACTTATTAGTTCAGCCCCAGTAGTTGGAGCAGTGGTTTCAAGACCTATTGCCACAAAAACAAACTTCTTATCTCTCTCCTTCTTAGCCATCTTTACTGCTTCACTTATACTATAGACAATTCTAACATCACAACCCTCAGATTGCTTTTCCATCAAAGATTTTTCACTTCCCGGCACTCTATACATATCTCCAAGAGTGGTTATTACATATCCATTGTCAGCTAAATATATGGCTGTATCTATCTCTTTTTGAGTTGTTACACAAACTGGACAACCCGGCCCTGGAACAACGGTTATATTCTCTGGCAGAACATCCCTAATCCCATACTTACAGATCGTGTGCTCATGACTTCCACAGACGTGCATAATCTTTAATTTATCTTCTTCTCAGCAAGTTTGTTAAAA
